# The impact of interfacial quality and nanoscale performance disorder on the stability of alloyed perovskite solar cells

**DOI:** 10.1038/s41560-024-01660-1

**Published:** 2024-10-30

**Authors:** Kyle Frohna, Cullen Chosy, Amran Al-Ashouri, Florian Scheler, Yu-Hsien Chiang, Milos Dubajic, Julia E. Parker, Jessica M. Walker, Lea Zimmermann, Thomas A. Selby, Yang Lu, Bart Roose, Steve Albrecht, Miguel Anaya, Samuel D. Stranks

**Affiliations:** 1 Department of Chemical Engineering and Biotechnology, University of Cambridge, Cambridge, UK; 2 Cavendish Laboratory, University of Cambridge, Cambridge, UK; 3 Division Solar Energy, Helmholtz-Zentrum Berlin für Materialien und Energie GmbH, Berlin, Germany; 4 Diamond Light Source, Harwell Science and Innovation Campus, Didcot, UK; 5 Institute of Materials Science of Seville, Spanish National Research Council−University of Seville, Seville, Spain

**Keywords:** Devices for energy harvesting, Solar cells, Wide-field fluorescence microscopy

## Abstract

Microscopy provides a proxy for assessing the operation of perovskite solar cells, yet most works in the literature have focused on bare perovskite thin films, missing charge transport and recombination losses present in full devices. Here we demonstrate a multimodal operando microscopy toolkit to measure and spatially correlate nanoscale charge transport losses, recombination losses and chemical composition. By applying this toolkit to the same scan areas of state-of-the-art, alloyed perovskite cells before and after extended operation, we show that devices with the highest macroscopic performance have the lowest initial performance spatial heterogeneity—a crucial link that is missed in conventional microscopy. We show that engineering stable interfaces is critical to achieving robust devices. Once the interfaces are stabilized, we show that compositional engineering to homogenize charge extraction and to minimize variations in local power conversion efficiency is critical to improve performance and stability. We find that in our device space, perovskites can tolerate spatial disorder in chemistry, but not charge extraction.

## Abstract (web summary)

Conventional microscopy is unable to correlate nanoscale properties of metal halide perovskites with the performance of solar cells. Frohna et al. present a multimodal operando microscopy toolkit as a diagnostic tool to address the issue.

## Main

Halide perovskites exhibit optoelectronic properties dominated by nanoscale variations in their structure^1,2^, composition^3,4^ and photophysics^5,6^. Compositional engineering^7–9^, contact engineering^10–12^ and surface passivation^13–15^ are established strategies to increase the performance of halide perovskite solar cells. However, the specific effects of bulk and interface modulation across different length scales on perovskite solar cell performance and stability remain poorly understood. This is in large part because most past microscopy work focused on perovskite thin films on insulating substrates and are therefore blind to charge extraction losses and additional recombination losses introduced by transport layers^12,16,17^.

To gain a complete, nanoscale understanding of device performance and degradation of next-generation optoelectronic technologies including halide perovskites, it is crucial to develop microscopy techniques capable of measuring complete devices under operational conditions. The current–voltage (JV) curve is an essential macroscopic measure of both the recombination and transport losses in a solar cell. Measuring full device stacks under operation at different points on the JV curve is essential to reveal information about charge transport and extraction in addition to non-radiative power loss channels. Several techniques have been demonstrated to probe particular points on the JV curve microscopically^18^: the short circuit current (*J*_SC_)^4,19,20^, open circuit voltage^3,16,21^ (*V*_OC_) or by fitting a pre-determined diode model, the entire JV curve^22^.

Here we rapidly extract local, microscopic JV curves (without a preconceived diode model^23–25^) on operating solar cells by employing voltage-dependent photoluminescence (PL) microscopy. We combine this voltage-dependent PL with absolutely calibrated hyperspectral PL^3,21^ and synchrotron X-ray nanoprobe fluorescence^26,27^ (nXRF) to map the optoelectronic properties and chemical composition of the halide perovskite absorber layer on the same scan area. We apply this powerful multimodal microscopy suite to an array of state-of-the-art, alloyed halide perovskite absorber layers fabricated into device stacks relevant for tandem solar cells^10,28,29^ before and after accelerated operational stress with industry-standard protocols (Methods)^30^ to reveal how the microscale distributions of composition, recombination and charge transport play critical roles in dictating both device performance and stability. We identify a local metric of performance, which takes into account local variations in both charge transport and recombination losses—power conversion efficiency (PCE) disorder. We find that devices with lower PCE disorder correlate with higher initial performance and are also more stable under operational stress. By contrast, more disordered devices tend to be less stable and exhibit more severe phase segregation during stress. We demonstrate that while PCE disorder can also be reduced (and initial performance boosted) with surface passivation or engineering, this treatment can be a double-edged sword as a poorly stabilized interface, even if passivated, can cause catastrophic device degradation. Our measurements reveal the complex interplay between local chemistry, transport and recombination in state-of-the-art perovskite solar cells.

## Multimodal operando microscopy toolkit

**Fig. 1:**
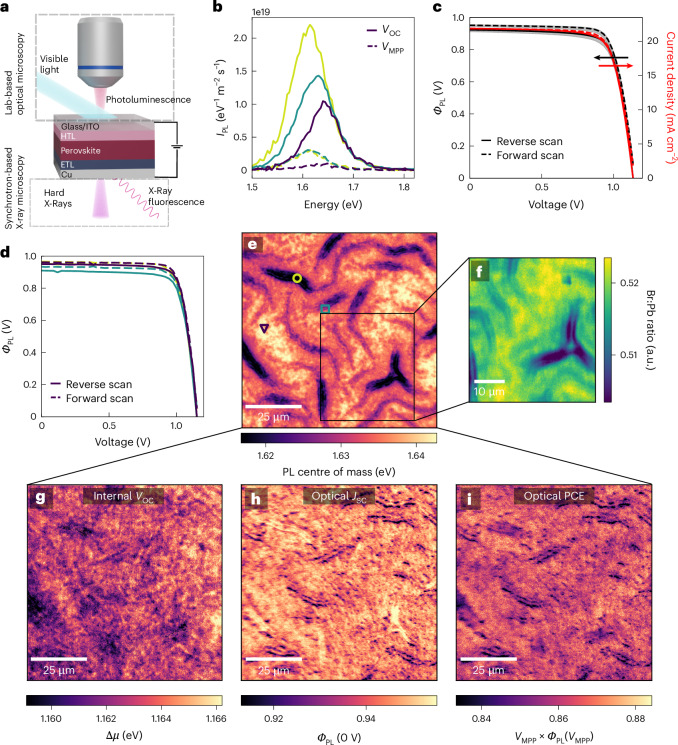
Device operando microscopy reveals DCDH solar cell performance is tolerant to even dramatic spatial optoelectronic and chemical heterogeneity. **a**, Schematic of perovskite solar cell under bias being illuminated either by a white light-emitting diode (LED) array for the luminescence measurements or monochromatic hard X-rays for the nXRF measurements. Note that the optical and X-ray measurements are not simultaneously acquired. **b**, Hyperspectral PL spectra (at *V*_OC_ and *V*_MPP_) of regions marked in **e**. **c**, Comparison of electrical JV curve (red, red arrow points to corresponding *y* axis) and area-averaged optical JV curve (black, black arrow points to corresponding *y* axis) of DCDH solar cell. Grey shaded areas show the distribution of JV curves across the map. **d**, Optical JV curves of the marked regions in **e**. **e**, PL centre of mass (COM) energy plot of a region of a DCDH solar cell at *V*_OC_. **f**, Br:Pb map from the marked region in **b** extracted by nXRF. **g**, Internal *V*_OC_ (Δ*µ*). **h**,**i**, Optical short circuit current extraction efficiency (*Φ*_PL_(0 V)) (**h**) and optical PCE (*V*_MPP_ ✕ *Φ*_PL_(*V*_MPP_)) of the same region as shown in **e** (**i**).

We have developed a platform to measure local, spectrally resolved PL on devices under bias, allowing extraction of device performance parameters combined with local chemical composition from synchrotron nXRF mapping on the same scan area (Fig. 1a). We use this to first study perovskite solar cell devices fabricated on [2-(9H-carbazol-9-yl)ethyl]phosphonic acid (2PACz), a state-of-the-art, self-assembled monolayer (SAM) hole transporting layer (HTL), with the complete device stack consisting of glass/indium tin oxide (ITO)/SAM/perovskite/C_60_/SnO_2_/Cu (Fig. 1a, Methods and Supplementary Fig. 37 for half device stacks). We employ a double-cation double-halide (DCDH) FA_0.83_Cs_0.17_Pb(I_0.83_Br_0.17_)_3_ perovskite composition that has previously been incorporated into high-efficiency single-junction and tandem solar cells^31^ and reproducibly demonstrates high performance (Supplementary Fig. 38).

X-ray diffraction (XRD) measured of the DCDH devices shows the expected pseudocubic perovskite pattern (Supplementary Fig. 39). PL centre of mass energy (COM) maps, showing the spectrally weighted average emission energy, are extracted from the local PL spectra at each point (Fig. 1b,e). The COM map shows the presence of a distinct wrinkled morphology that imprints itself onto the emission energy of the perovskite (Supplementary Note 6). Interestingly, wrinkled areas exhibit red-shifted emission that correlates with a reduction in the relative Br content as revealed by nXRF on the same region (Fig. 1b,f), although we note that the red-shifted emission may be partially explained by photon re-absorption in the thicker wrinkles^32,33^. While these measurements were performed with the device held at open circuit voltage (*V*_OC_), the quenching of PL as a function of bias gives information about charge carrier extraction. By sweeping the voltage and comparing the broadband PL intensity (*I*_PL_) at each point to its value at open circuit, the current extraction efficiency—the fraction of carriers extracted by the contacts, *Φ*_PL_(V)—and corresponding optical JV (*J*(*V*)) curves normalized by the generation current (*J*_gen_) at each point can be extracted^24^ (Methods, Supplementary Note 2 and Supplementary Figs. 1–7):

$${\it{\Phi }}_{\mathrm{PL}}\left(V\,\right)=\frac{{I}_{\mathrm{PL}}\left({V}_{\mathrm{OC}}\right)-{I}_{\mathrm{PL}}\left(V\,\right)}{{I}_{\mathrm{PL}}({V}_{\mathrm{OC}})}\approx \frac{J\left(V\,\right)}{{J}_{\mathrm{gen}}}$$

The spatially averaged optical JV and electrical JV measurements show excellent agreement (Fig. 1c; also Supplementary Note 2 provides further analysis and drift-diffusion simulations). We note that as the solar cell deviates substantially from typical diode behaviour, particularly after substantial degradation, deviations emerge between the optical and electrical measurements, which can be taken into consideration (Supplementary Note 3). From the hyperspectral PL and optical JV curves extracted from each point, we extract local device figures of merit. Fitting PL spectra with the generalized Planck’s law enables the extraction of the quasi-Fermi level splitting (Δ*µ*) and the bandgap (*E*_g_)^34^. Δ*µ* is a measure of the internal voltage of a solar cell, and in the absence of energetic offsets and recombination at the contacts^12^, should approximate the electrically measured external *V*_OC_. In Fig. 1f, we show a map of Δ*µ* for the DCDH solar cell at open circuit. Comparing these values (mean of ~1.15 eV) to the electrically measured *V*_OC_ (1.15 V), we determine that there is a negligible energetic losses between the perovskite and the contacts^12,35^. Maps of equivalent half stacks in Supplementary Fig. 37 show similar spatial variation and show that the C_60_ interface is an active site for non-radiative recombination. This interface reduces the Δ*µ* by ~80 meV, corresponding to more than an order of magnitude loss in luminescence efficiency, as has been previously observed^16,36^.

Strikingly, the wrinkled areas do not appear to negatively affect the Δ*µ* values (Supplementary Note 6). PL spectra from this region (Fig. 1b) reveal these wrinkled regions have sufficiently increased PL intensity to almost exactly counteract the expected Δµ loss caused by their reduced bandgap compared to surrounding regions and are therefore benign to local voltage losses (Supplementary Notes 5 and 6). Figure 1h shows that the optical current extraction efficiency *Φ*_PL_(0 V) is also spatially homogeneous, although a subset of the wrinkles exhibits slightly worse charge extraction. In Fig. 1i, we define the optical power conversion efficiency (PCE) as the product of the maximum power point voltage and the current extraction efficiency at this voltage (*Φ*_PL_(*V*_MPP_) × *V*_MPP_, units of V). We find a tight optical PCE spatial distribution of less than ±5% relative, particularly striking given the morphological and optoelectronic variations of the perovskite itself. Representative JV curves extracted from pristine and wrinkled areas are shown in Fig. 1d, highlighting the spatial PCE homogeneity and the relatively small impact wrinkles have on PCE; such a conclusion is in contrast to previous works suggesting that these wrinkles may be detrimental to device performance and longevity^37,38^. This overall good spatial homogeneity is a hallmark of highly efficient, stable devices as we will discuss further below. The correlation between Br:Pb and several device figures of merit summarizes our findings of the relationship between local chemistry and performance: whereas the local Br:Pb ratio does modulate the bandgap and the non-radiative voltage loss, it has little effect on either Δ*µ* or PCE (Supplementary Note 5), showing that this device stack is very tolerant to chemical disorder.

## Microscopic effects of device operation and degradation

We now subject the devices to extended operational stress to probe the interplay between microscopic changes to solar cell performance and local composition/optoelectronic properties under operational stress. Specifically, the unencapsulated cells are held at open circuit voltage under continuous 1 sun illumination at 65 °C and continuous nitrogen flow for 100 h following the standardized stability test ISOS-L-2I (ref. ^30^) (Methods). Holding cells at open circuit under illumination and added external heat is a considerably greater stability challenge compared to a maximum power point track so is ideal for an accelerated operational stress test^39,40^.

**Fig. 2:**
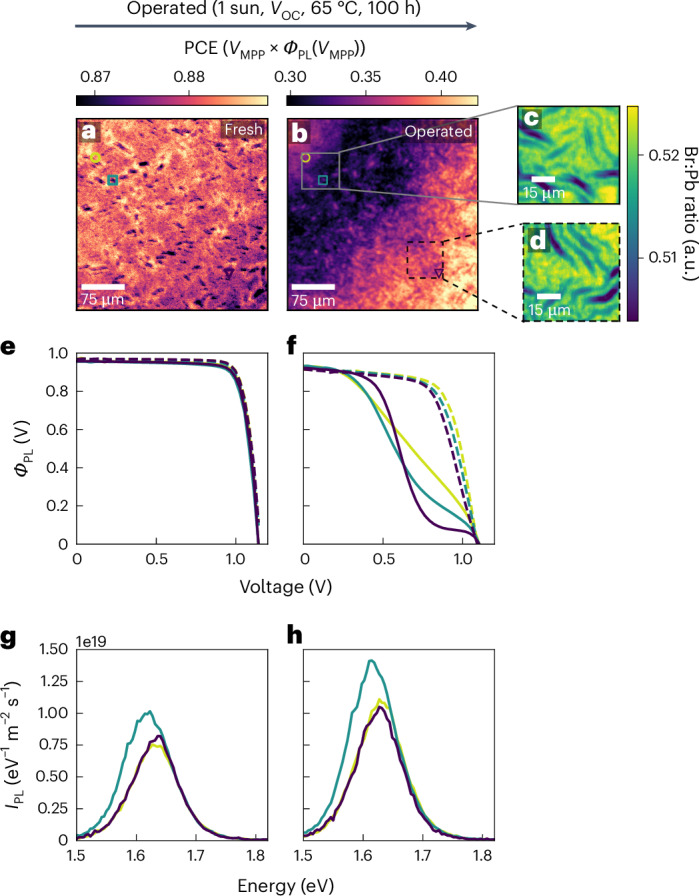
Local reductions in performance are evident in microscopic JV curves of DCDH perovskite solar cells after extended operation. **a**,**b**, Optical PCE maps of the same area of a fresh (**a**) and operated (**b**) DCDH solar cell after 100 h at *V*_OC_, 65 °C and 1 sun illumination. **c**,**d**, show Br:Pb ratio maps extracted from nXRF from the two regions marked in **b** after the 100 h of operation. **e**,**f**, Optical JV curves before (**e**) and after (**f**) ageing from the points marked in **a** and **b**. Solid lines are reverse scans, dashed lines are forward scans. **g**,**h**, PL spectra from the same marked areas before (**g**) and after (**h**) ageing.

Figure 2a shows that the initial spatial variation in optical PCE is extremely small (±2% relative). However, upon remeasuring the same area after the operational stress test (Fig. 2b), a large global drop in optical PCE is observed along with a striking increase in spatial PCE heterogeneity characterized by a diagonal progression across the sample. The local optical JV curves extracted from the regions marked in the PCE maps reveal that the operation-stressed samples exhibit spatially varying hysteresis behaviour between the forward and reverse voltage scans (Fig. 2e), which was absent before operation (Fig. 2f). Some regions such as the one marked in the bottom right corner of Fig. 2b display a large S-kink in the JV curve where effectively no charge extraction is observed up to 0.3 V below *V*_OC_. Other regions (square and circle markers) display substantial, spatially varying degrees of apparent series resistance, a key parameter to which our technique is sensitive (Supplementary Note 2). This extraction issue is mainly present in the reverse scan whereas much more spatially homogeneous, higher optical PCE is observed in the forward scan (Supplementary Note 7 and Supplementary Fig. 40). However, we see no shifting in the local PL peak position of the perovskite before and after operation and a global increase in PL intensity at *V*_OC_ is apparent (Fig. 2g,h and Supplementary Fig. 41); these results suggest that the bulk of the perovskite absorber layer itself has not been degraded after the extended operational stress and that the issues lies at the interfaces with the contacts, as we will discuss further in a later section.

The observation that the perovskite bulk has not degraded is further supported by compositional maps in Fig. 2c,d displaying regions of the scan area that exhibit starkly different optical PCE distributions but very similar chemical distributions, with spatial chemical distributions (Br, Pb, I, Cs and ratios thereof) of the perovskite in fresh and operated devices showing no appreciable change (Supplementary Figs. 42 and 43). Intriguingly, the wrinkles also appear to be enriched with Cs as has been proposed previously^41,42^. It should be noted that the low-energy Cs and I nXRF lines are more prone to errors due to self-absorption effects^26,43^, particularly in the thicker wrinkles. These same results are seen in multiple measurements across different scan areas, devices and batches (Supplementary Figs. 44–50). Results such as these demonstrate the power of our operando microscopy technique over conventional microscopy at open circuit, which would be blind to such degradation; further, PL at open circuit cannot be used alone as a performance metric.

The stark spatial variation of the voltage-dependent PL shows a degradation front extending from the edge of the active area defined by the overlap of the metal and ITO electrodes. Recent reports have shown that mobile ionic species in the perovskite are particularly problematic at the edges of active device areas and could cause degradation consistent with what we observe in Fig. 2b,d (ref. ^44^). Large-area scans of whole device pixels highlight that this degradation front appears from the edges and can even appear in pristine, as-made devices if there are issues with fabrication (Supplementary Figs. 51 and 52). Macroscopic hysteresis in the JV characteristics of perovskite solar cells has been attributed to the presence of mobile ions that can screen electric fields^45,46^. Given that the observed difference between local forward and reverse optical JV scans is large, we attribute this difference to spatially varying concentrations of mobile ions rather than a resistive effect. Looking further away from the edge of the contacts, the spatial variations in optical PCE and hysteresis are reduced (Supplementary Figs. 53–55). The macroscopic electrical JV curve in Supplementary Fig. 56 shows a hysteresis in between these two extremes, highlighting that the macroscopic hysteresis is an ensemble average of many locally varying mobile ionic concentrations. These observations suggest that cell and module designs preventing edge effects will also play an important role in boosting the stability of perovskite solar cells.

## Stress-induced phase segregation in triple-halide devices

To investigate how compositional tuning affects microscopic device performance and stability, we now consider the same device architecture but with the double-cation triple-halide (DCTH) FA_0.83_Cs_0.17_Pb(I_0.81_Br_0.16_Cl_0.03_)_3_ composition in which Cl is present in the system introduced through PbCl_2_ in the precursor solution. The addition of PbCl_2_ to the precursor solution increases the bandgap of the final perovskite (Supplementary Fig. 57), suggesting at least some Cl incorporates into the structure, consistent with recent reports using these compositions for top cells in high-performance multijunction devices^29,47^. Macroscopic electrical JV measurements show an increase in *V*_OC_ and corresponding decrease in *J*_SC_, compared to the DCDH analogue, as expected with the increased bandgap. However, there is also a reduction in fill factor, suggesting that the additional compositional alloying has had a small negative effect on carrier transport and extraction (Supplementary Fig. 38).

**Fig. 3:**
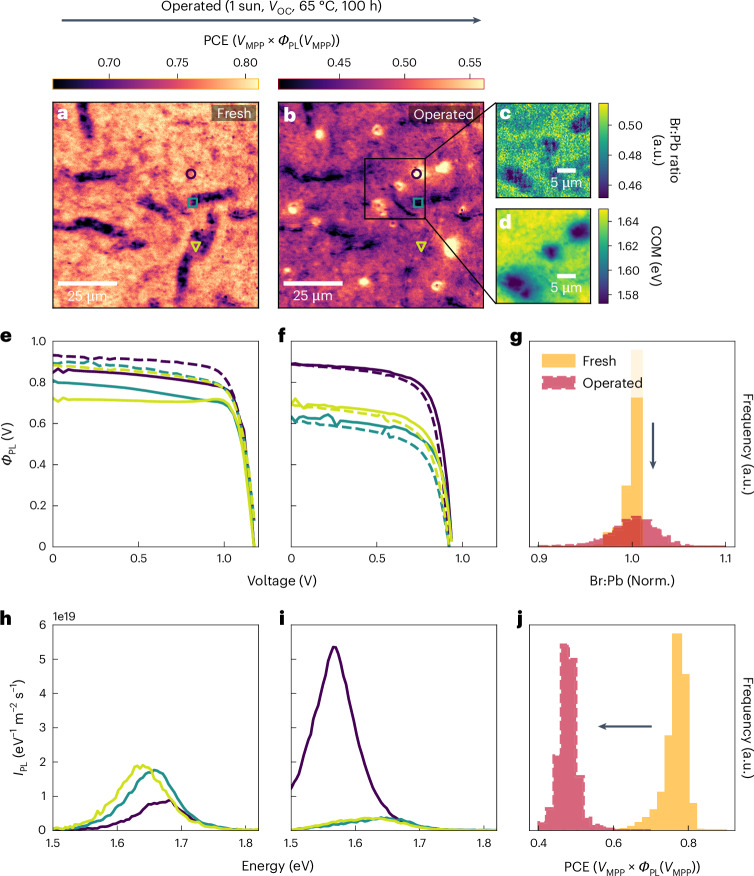
Multimodal microscopy on DCTH perovskite solar cells reveals reduced device stability and increased microscopic phase segregation compared to DCDH analogues. **a**,**b**, Optical PCE maps of the same area of a fresh (**a**) and operated (**b**) DCTH solar cell after 100 h at *V*_OC_, 65 °C and 1 sun illumination. **c**,**d**, Br:Pb ratio (**c**) and PL centre of mass energy maps (**d**) extracted from the region marked in **b** after the 100 h of operation. **e**,**f**, Optical JV curves (**e**) before and after (**f**) operational stress from the points marked in **a** and **b**. Solid lines are reverse scans, dashed lines are forward scans. **g**, Normalized Br:Pb ratio histograms for a pristine sample (orange) and the marked region of the operated (red) sample. **h**,**i**, PL spectra extracted from the same marked areas before (**h**) and after (**i**) ageing. **j**, Optical PCE histogram for this DCTH solar cell before (orange) and after (red) operation. Arrows in **g** and **j** are guides to the eye from fresh to operated.

A wrinkled morphology with similar surface area coverage and size to the DCDH solar cells is also observed in the DCTH devices (Supplementary Note 6). The optoelectronic behaviour of the wrinkled areas is more varied as some wrinkles now show increased Δ*µ* compared to their surroundings, whereas other regions are reduced (Supplementary Fig. 58). However, interestingly, the wrinkles have a more substantial impact on the local PCE with relative reductions of up to 20% compared to pristine areas (Fig. 3a). This is emphasized by the much greater spatial variance in pseudo-JV curves extracted from across the pristine device (Fig. 3e). There is therefore a much greater spatial PCE heterogeneity in the DCTH sample (Fig. 3j) compared with the tight PCE distribution of the relatively homogeneous DCDH (Supplementary Fig. 59). The fill factor values extracted from the optical JV curves in Fig. 3c are also markedly reduced, indicating that the chloride introduces additional nanoscale disorder that hampers charge extraction while increasing non-radiative recombination, suggesting that alloying needs to be carefully managed in halide perovskites on both macro and nanoscales.

After the same extended operational stability test, we see substantially greater degradation in these solar cells than in the DCDH analogues. Whereas the DCTH cells generally lost over 40% of their initial PCE (Fig. 3j), we observed the emergence of ~5 μm regions with reduced PCE losses and a PL peak that red-shifted by 0.1 eV and increased in intensity by a factor of 5 (Fig. 3b,h,i). Combining nXRF and hyperspectral PL, we find that these areas show a large local reduction in Br content and assert that this is due to exaggerated local halide segregation^3,48^ driven by the combination of light and heat stressors in the operational test. In most regions, there is a reduction in both optical short circuit current and *V*_OC_ (Fig. 3f), meaning an increase in losses caused by both charge extraction and non-radiative recombination. By contrast, the phase-segregated regions display a higher charge extraction efficiency and PCE than their surroundings, suggesting a mitigation of degradation pathways related to charge extraction.

The relative increase in PL intensity in the phase-segregated areas is not sufficient to compensate for the voltage loss caused by the dramatic red shifting of the PL peak and so these regions exhibit a lower Δ*µ* compared to their surroundings (Supplementary Figs. 58, 60 and 61). The segregation persists even after extended storage in the dark, as nXRF maps show clear compositional segregation and increased spatial variance in the Br:Pb signal when measured several days after the operational stability test (Fig. 3c,d). Here the microscopy suite has revealed the microscopic impact of phase segregation on device stability and performance—an ongoing issue for attaining stable tandems^49^. Unlike the DCDH composition, the DCTH devices also show a transient increase in PL intensity during voltage sweeps, causing *I*_PL_(*V*_OC_) to differ in the reverse and forward scans (a vertical offset close to 0 V) and affecting the observed optical JV curves. We refer to this phenomenon as ‘optical hysteresis’, and it is clearly observable in optical JV curves extracted from the DCTH sample as shown in Fig. 3e (Supplementary Note 7 provides discussion of hysteresis). Taken together, our measurements on the DCTH samples show that the addition of the PbCl_2_ into the otherwise unchanged precursor solution meaningfully increases the bandgap of the resulting perovskite, but at the cost of increased spatial PCE disorder and hysteresis, ultimately hampering charge carrier extraction, phase and device stability relative to the control DCDH devices.

As a further compositional tuning step, we added MACl to the DCTH solution to produce triple-cation triple-halide (TCTH, MA_0.03_FA_0.81_Cs_0.16_Pb(I_0.81_Br_0.16_Cl_0.03_)_3_) solar cells—the composition used in many of the highest performing perovskite tandems including our own^13,29,47^. The additional compositional disorder in the precursor solution corresponds to slightly increased spatial disorder in the device (Supplementary Fig. 63) and a corresponding slight reduction in initial device performance (Supplementary Fig. 38). However, surprisingly, the incorporation of MA results in a large increase in both phase and device stability. After operational stress, there is little to no loss in either open circuit PL intensity or *J*_SC_, and phase segregation is effectively suppressed (very minor effect on Δ*µ*), although not eliminated entirely (Supplementary Figs. 64–66)—a marked improvement over the DCTH in all aspects. Overall, we have revealed that even minor compositional variations in the precursor solution can have a substantial impact on the microscopic and thus macroscopic behaviour of wide-bandgap perovskite solar cells under operational stress.

## Stability effects of interfacial engineering and passivation

**Fig. 4:**
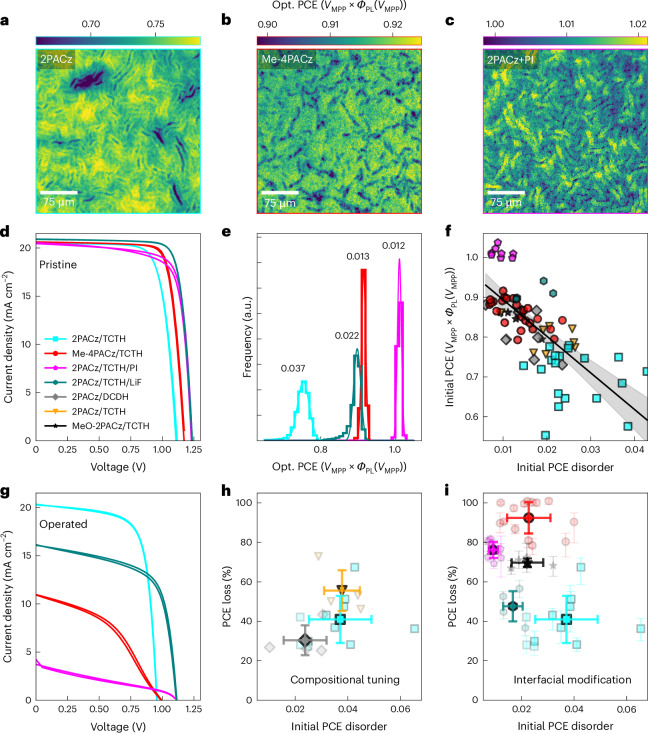
Interfacial chemistry and spatial PCE disorder predict performance and stability of mixed-cation, mixed-halide perovskite solar cells. **a**–**c**, Optical PCE maps of pristine control 2PACz/TCTH (**a**), Me-4PACz/TCTH (**b**) and 2PACz/TCTH + PI (**c**) passivation devices. **d**, Representative JV curves of the pristine interface-modified devices mapped in panels **a**–**c**. **e**, Optical PCE distributions and corresponding Gaussian fits of the interface-modified devices. The numbers over the distributions represent the FWHM of the distribution. **f**, Initial optical PCE disorder (FWHM of PCE distribution) versus initial PCE (mean of PCE distribution) for a range of perovskite devices, where each point is an individual device. Linear regression shows a Pearson’s *r* value of −0.71, Spearman’s *r* value of −0.77 and a *P* value of «0.01 (two-sided tests). Shaded regions represent the 95% confidence interval of the linear fit from a Student’s *t* distribution percent points function (*n* = 75 devices). **g**, JV curves after operational stress of the representative devices shown in **d**. **h**, Scatter plot of initial PCE disorder versus PCE loss (%) during operation for the perovskite composition series on 2PACz. (Device numbers for comparison are DCDH = 4, DCTH = 4, TCTH = 8). **i**, Scatter plot of initial PCE disorder versus PCE loss (%) during operation for the interfacial modification series. (Device numbers for comparison are 2PACz/TCTH = 11, MeO-2PACz = 4, Me-4PACz/TCTH = 14, 2PACz/TCTH + PI = 8, 2PACz/TCTH+LiF=4). Solid markers are the mean of a given device type, semi-transparent markers are individual devices. Error bars in **h** and **i** are standard deviations.

To further understand performance issues and instabilities in high-efficiency devices, we now explore contact engineering and surface passivation. Here we fix the TCTH perovskite composition due to its stability and utility in tandems and study the impacts of modulating both surfaces independently. We vary the HTL from 2PACz to MeO-2PACz and Me-4PACz, two other SAM HTLs that have been used in high-efficiency solar cells^13,28^ and have been shown to affect charge extraction, interfacial recombination, energetic alignment and substrate hydrophobicity^3,12,28,50^. We additionally fix the 2PACz HTL while passivating the perovskite/C_60_ interface with either a thin LiF interlayer or the ionic liquid piperazinium iodide (PI), both of which have been shown to substantially boost *V*_OC_, adjust surface charge and modulate charge extraction^13,28,36,51,52^. We show optical PCE maps of the 2PACz control, Me-4PACz and 2PACz+PI passivation TCTH devices in Fig. 4a–c (Supplementary Figs. 67–73 for MeO-2PACz, Me-4PACz and 2PACz+LiF passivation). Whereas the samples display similar morphology, the optical PCE maps of the Me-4PACz and 2PACz+PI/LiF-passivated devices show higher mean values than the 2PACz control and exhibit lower spatial variation in performance (Supplementary Figs. 74–76 for comparisons). This is reflected in the electrical JV curves of the devices (Fig. 4d) where both passivation strategies produce a *V*_OC_ of ~1.25 V, a boost of 0.1 V over the control and the Me-4PACz device shows a more modest spatial homogenization of optical PCE and boost in *V*_OC_. We note that the optical PCE of the PI-passivated devices is partially overestimated due to shunting (Supplementary Note 2), whereas this is not the case for the LiF devices.

To quantify the spatial heterogeneity in the optical PCE distributions for each sample, we fit optical PCE histograms with a Gaussian function and define the ‘Initial PCE Disorder’ as the full width at half maximum (FWHM) of the fitted Gaussian peak. Histograms and corresponding fits are shown in Fig. 4e. These data lead to a key general observation that lower optical PCE disorder correlates with higher-performing devices. We perform this analysis across our entire device range consisting of three perovskite compositions, three transport layers and two surface passivation treatments and find that this observation holds across this wide device space covering state-of-the-art perovskite developments for tandem solar cells (Fig. 4f).

We then expose the interface-modified devices (TCTH devices with varying HTL and/or surface passivation) to the same operational stress test as the compositionally engineered samples (Supplementary Figs. 67–73 for maps). JV curves after stress are shown in Fig. 4g. We find that the control device almost fully retains its original *J*_SC_ but has lost voltage without any loss in PL intensity—suggesting some losses at the contacts whereas the bulk perovskite remains relatively pristine. By contrast, the device with the adjusted HTL Me-4PACz has substantially degraded electronically (from JV scan) together with a loss in PL intensity (although on its own not sufficient to explain the drop in *V*_OC_, Supplementary Figs. 77 and 78), suggesting the interface is to blame for these losses. The PI- and LiF-passivated devices show an equivalent *V*_OC_ loss (and a corresponding loss in PL intensity), whereas the LiF device retains substantially more *J*_SC_. We note that neither the controls nor the passivated devices show any substantial changes in the XRD pattern of the absorber layers^53^, consistent with the issues being localized to interfaces rather than any bulk structural changes (Supplementary Fig. 79).

To summarize our stability findings, we find that all device types containing Cl exhibited increased transient electronic behaviour after the operational stress test (Supplementary Fig. 80) and a larger loss in *V*_OC_ compared to the loss of internal voltage Δ*µ* (or lack thereof in the 2PACz/TCTH case; Supplementary Fig. 81). The transient behaviour and mismatch between internal and external voltages suggest that mobile ions in our Cl-containing devices play a larger role after operational stress compared to double-halide equivalents. In the perovskite compositional tuning series with fixed, relatively stable interfaces, initial optical PCE disorder is a good predictor of overall device stability (Fig. 4h), and one could therefore screen and predict for stable devices by looking at the optical PCE distributions in the as-made devices. However, the same is not necessarily true for interfacial modification in the case of unstable interfaces. The array of interfacial modification methods we tested resulted in improved initial electrical performance and reduced optical PCE disorder, but some also caused dramatically poorer stability (Fig. 4i). Modifying either interface while keeping both the perovskite composition and the rest of the device stack constant can drastically change the stability of the device. Such a conclusion is consistent with recent observations about a trade-off between efficiency and stability in perovskite LEDs^54^.

## Discussion

Our microscopic operando studies of a wide device space now allow us to propose general design rules towards practical realization of efficient and stable perovskite solar cells. The combination of local chemistry, luminescence properties and optoelectronic performance allows us to discern which features are causing degradation and which are most important to address microscopically—providing critical impact on overall performance and stability. The first is that interfacial optimization must be the top priority for understanding and stabilizing the interfaces that impact the long-term stability of the device. This includes engineering both the quality of the perovskite near the contact and the interface with the contact itself. A growing body of literature suggests that without care, interfaces prove to be the limiting factor for long-term stability^55–58^. Our systematic approach has enabled us to decouple the losses and degradation in the bulk and at interfaces.

We have shown that initial PCE can be rapidly lost if the interfacial stability is compromised, regardless of initial performance metrics or PCE homogeneity. This highlights an ongoing challenge in the field to find an appropriate passivation for wide-bandgap perovskite top cells that reproducibly deliver both high efficiency and stability. Techniques for depth-sensitive mapping of chemical composition in complete devices would further increase the understanding of these interfaces^59^. If stable interfaces are achieved, the second crucial factor affecting performance and stability is the local optical PCE disorder, which can be tuned via a combination of perovskite composition and interfacial chemistry. In the most severe cases, increasing initial compositional disorder can seed the formation of micron-scale, extremely phase-segregated domains exhibiting unexpected charge extraction behaviour. This multimodal operando methodology has revealed that variations in composition, morphology and bandgap can be tolerated in efficient devices with stable interfaces, unlike more damaging local losses in charge extraction, a conclusion that could not be demonstrated through macroscopic device measurements and conventional PL microscopy alone.

Operando microscopy of devices can therefore be a powerful predictive screening tool and time saver for engineering long-term operational stability. The robustness of our systems of study to compositional disorder is due to the presence of charge carrier funnelling into high optoelectronic quality, low-bandgap regions, which maintains a higher Δ*µ* as we have previously reported in neat films^3^. Alternative perovskite compositions that do not exhibit efficient funnelling or have more defective low-bandgap sites may not benefit from this tolerance to chemical disorder. Our multimodal toolkit additionally enables detailed ‘post-mortem’ examinations of devices after extended stress testing—revealing detailed information on the chemical, optical and electronic properties of the entire device stack and what has degraded. This then enables us to predict instability in as-made devices and could be a useful quality control tool during manufacturing to guide process optimization.

Taken together, our measurements show that subtle changes to perovskite composition and interfacial chemistry have serious consequences for the degradation mechanisms at play in these devices, and we pinpoint the location and cause of the degradation. Changes in the defect density at an interface may render a kinetically slow process suddenly favourable, or changes in the hydrophobicity of the contact layer may change the crystallization kinetics of the active layer sufficiently to cause large-scale heterogeneities that can seed degradation^60^. These findings emphasize the need to optimize devices simultaneously for both performance and robust, reproducible stability. It also urges caution for the generality of proposed degradation mechanisms because minor variations may alter the ‘weak link’ of stability in the devices of interest. These measurements show that the nanoscale device performance landscape is remarkably complex in perovskite solar cells. Microscopic platforms such as that demonstrated here that can measure local optoelectronic and charge extraction properties are crucial to understanding the degradation pathways in disordered semiconductor devices as they generate powerful insights^19^ and unveil mechanistic pathways which need to be addressed for the success of these devices. Metal halide perovskites were used as a case study in this work, but the techniques developed for the multimodal, microscopic toolbox are generalizable to a broader class of light harvesting and emitting devices based on disordered materials, and exciting insights may be gained in areas such as organic and copper–indium–gallium selenide photovoltaics and InGaN light-emitting diodes. A scaled version of the operando optical component of the toolkit could be used as a quality control screening methodology for large-scale photovoltaic manufacturing.

## Methods

### Materials

Lead salts were purchased from TCI Chemicals (>99.99% purity trace metals basis), FAI and MABr from Dyenamo (>99% purity), CsI from abcr GmbH (>99% purity) and MACl (>99% purity) from Sigma Aldrich. The salts were used as received without further purification. Solvents were purchased from Merck (>99.8% purity, anhydrous) and were used as received without further drying or purification. Piperazinium iodide was synthesized in house as described in ref. ^13^. Briefly, one equivalent of piperazine was dissolved in ethanol (15 ml g^−1^) and the vessel immersed in an ice-water bath. Two equivalents of a 57% hydroiodic acid in water solution were added dropwise into the cooled vessel and the mixture was stirred for 30 min. Upon completion, the solvent was evaporated and the solid rinsed with ethyl acetate.

### Film and device fabrication

Solar cells were fabricated on ITO substrates, which were subsequently cleaned in a Mucasol solution (2% in deionized water), deionized water, acetone and 2-propanol, each for 15 min in an ultrasonic bath. Afterwards, the surface was ‘activated’ for 10–15 min in an UV-O3 cleaner (FHR UVOH 150 Lab), which is a crucial step before SAM deposition. The SAM solutions (1 mM l^−1^ in ethanol) was spin coated at 3,000 rpm for 10 s, after which the substrate was annealed at 100 °C for 3–10 min. All spin-coating layer deposition steps were conducted in a nitrogen atmosphere.

The cell configuration is ITO/SAM/Perovskite/C60(20 nm)/SnO2(20 nm)/Cu(100 nm), where the C_60_ and Cu were deposited by thermal evaporation and the SnO_2_ layer was deposited by atomic layer deposition in an Arradiance GEMStar reactor. There was no air exposure between any of the layer deposition processes. Tetrakis(dimethylamino)tin(IV) (TDMASn) was used as the Sn precursor and was held at 60 °C in a stainless-steel container. Water was used as the oxidant from a stainless-steel container without active heating, whereas the precursor delivery manifold was heated to 115 °C. For the deposition at 80 °C, the TDMASn/purge1/H_2_O/purge2 times were 1 s/10 s/0.2 s/15 s with corresponding nitrogen flows of 30 sccm/90 sccm/90 sccm/90 sccm. With this, 140 cycles lead to 20 nm of SnO_2_.

Perovskite layers: first, a 1.4 M ‘FACs’ solution (FA, Cs, PbI2, PbBr2; 22% Cs & 15% Br) in 3:1 DMF:DMSO was shaken at room temperature overnight (solution for the DCDH perovskite). For the DCTH composition, this solution was transferred into another vial that contained PbCl_2_ powder and shaken for 1 h at 60 °C before perovskite layer deposition, with a nominal molar Cl percentage of 5%. For the TCTH composition, this second vial contained both PbCl_2_ and MACl. Exemplary amounts of chemicals for 1 ml of 1.4 M solution: 500 mg PbI_2_, 116 mg PbBr_2_, 188 mg FAI, 80 mg CsI (weighed into one vial) + 4.7 mg MACl, 19.5 mg PbCl_2_ (in another vial).

The perovskite solution was spin coated at 3,500 rpm for 40 s and 250 µl anisole as the antisolvent was dripped at 28 s after start of the spinning, followed by 20 min annealing on a hotplate at 100 °C in N_2_.

For interface modification, either PI or LiF were deposited on the perovskite before C60 evaporation. For the PI treatment, 100 µl of a solution of 0.3 mg ml^−1^ piperazinium iodide in 2-propanol were dynamically spin coated at 5,000 rpm on the perovskite, followed by 2 min annealing at 100 °C. The sample was then washed with 100 µl 2-propanol and again annealed for 2 min at 100 °C. For LiF, 1 nm was thermally evaporated without breaking the vacuum.

### Bulk solar cell characterization

The ISOS-L-2I accelerated degradation protocol was carried out under an LED array solar simulator (Supplementary Fig. 82 provides spectral details). Unencapsulated devices were placed into an O-ring sealed chamber with a quartz window. The chamber was heated above by the LED array and below by a proportional-integral-derivative (PID) temperature-probe-controlled hotplate. The hotplate heat output was adjusted until the internal temperature of the chamber was 65 ± 1 °C as measured intermittently by an infrared temperature probe. Devices were kept at open circuit voltage due to the more stressful nature of this ageing protocol to devices versus maximum power point^19^.

The solar cells contained each six pixels with an active area of 0.16 cm² (overlap of patterned ITO and the Cu stripe, area confirmed with optical microscope), measured with an Oriel LCS-100 ABB solar simulator and Keithley 2400 source-measure unit inside a N_2_ glovebox. The JV was scanned in 20 mV steps with 20 ms integration time and 20 ms delay time between each voltage step and measurement. JV testing was performed without a mask. The solar simulator was calibrated using a reference KG3 filtered silicon solar cell calibrated by Fraunhofer ISE. The spectral mismatch between the desired spectrum and the solar simulator is ~0.997, within experimental error so no correction is applied.

### XRD

XRD patterns were obtained using a Bruker D8 ADVANCE and a Copper X-ray tube operating at 40 kV with Ka emission wavelength of 1.54 Å. The samples were measured in ambient air. The scan range for 2*θ* was from 7° to 40° with a step size of 0.01° and a dwell time of 0.55 s per angle.

### SEM

SEM micrographs were acquired using an FEI Helios FIB/SEM operated with an accelerating voltage of 2 kV, current of 0.2 nA and working distance of approximately 4 mm. An Everhart–Thornley detector acquired multiple secondary electron images consecutively (typically 32) at a pixel dwell time of 300 ns, which were overlaid to improve the signal to noise ratio. In some micrographs the sample stage was tilted to 40° such that the out-of-plane morphology could be observed.

### Hyperspectral operando luminescence microscopy

Hyperspectral microscopy is performed using a Photon Etc. IMA microscopy system. 20× (Nikon TU Plan Fluor, 0.45 NA) and 63× glass collar corrected objectives (Zeiss LD Plan-Neofluar 63x/0.75 Corr M27) with appropriate chromatic aberration corrections were used for all measurements due to their ability to focus through our ~1.1 mm thickness ITO/glass device substrates. The samples are stored in a nitrogen-filled glovebox before being transferred to the microscope for measurements. The devices are held in a custom 3D-printed device holder allowing individual pixels to be biased with the use of a Keithley 2450 sourcemeter. The devices are optically excited using a fibre-coupled LED array consisting of variable-power red, green and blue LEDs from Thorlabs (M455L4, M530L4, M617L3, respectively). The devices are illuminated with this LED array at an acute angle rather than at normal incidence through the objective. This is to ensure that the entire pixel was illuminated uniformly as illuminating a small spot on the device while leaving the rest in the dark caused considerable artefacts in the resulting data such as artificially reduced apparent optical current densities. This acute angle does not cause appreciable artefacts in our data due to the large refractive index or the wide-bandgap perovskite across the visible range. Assuming a refractive index of the perovskite of 2.55 (ref. ^61^), even light incident at close to 90° from the normal is bent to an angle of 22° from the normal, resulting in minimal shading from the wrinkles. The resulting relative path length increase is only 1.086. The power of the LEDs is adjusted so that the measured short circuit current density under the microscope matched the short circuit current of the device measured under our standard LED solar simulator. The powers are adjusted so that one third of the current came from each of the red, green and blue LEDs to crudely approximate a white light, solar illumination profile. An appropriate long pass filter (Semrock BLP01-664R) is placed in the luminescence collection path to remove any scattered excitation.

For the hyperspectral measurements, the device is held at a particular voltage (either open circuit or maximum power voltage). The device is illuminated as described above, emitted light is collected in the objective and is incident upon a volume Bragg grating which splits the light spectrally onto a high-sensitivity CMOS camera (Hamamatsu ORCA Flash 4.0 V3 sCMOS camera) with 2,048 × 2,048 6.5 × 6.5 µm^2^ pixels that is thermoelectrically cooled to −10 °C. A hyperspectral image is created by scanning the angle of the grating with respect to the emitted light. The microscope is calibrated for absolute number of photons to extract quantitative PL spectra using the methodology previously reported^3^. Details of data fitting to extract the quasi-Fermi level splitting and centre of mass are included in Supplementary Note 1.

Voltage-dependent photoluminescence mapping is performed using the Photon Etc. widefield microscope equipped with the large-area LED illumination area as described above. The set-up was used in broadband mode where the grating is rotated to the zeroth order diffraction and acts simply as a mirror. A Keithley 2450 sourcemeter is used to step the applied voltage between image acquisitions, performing sweeps from open circuit to short circuit, then back in a total of 80 steps. Scan rates are always kept to 0.01 V s^−1^ or below to avoid additional scan-rate-dependent hysteresis and to allow transient changes in luminescence and extracted current to stabilize at a given voltage^24^. Whereas the voltage-dependent PL measurement is occurring, a simultaneous macroscopic electrical JV measurement is also performed. Full details of data treatment to extract solar cell performance metrics are included in Supplementary Note 2.

### Nanoprobe synchrotron X-ray fluorescence

Synchrotron measurements were performed on the I14 hard X-ray nanoprobe beamline at Diamond Light Source Ltd., Didcot, United Kingdom. Samples were stored in an Ar-filled glovebox before measurements. The full experimental set-up has been described elsewhere^62^ and the experimental is very similar to that we have reported previously^3^. X-rays from an undulator source are monochromated to produce a 15 keV X-ray beam, which is focused by a pair of Kirkpatrick–Baez mirrors to produce a beam with a FWHM of approximately 50 × 50 nm at its focus. For mounting of the large solar cell substrates, a custom designed, 3D-printed sample holder was used to enable reproducible mounting on the nanoprobe endstation stages. The sample is placed at the focus and is laterally scanned across this focal point to produce the final maps. The energy-resolved nXRF signal is collected with a four-element silicon drift detector in a back-scattering geometry. The data were analysed in part with the open-source Python package Hyperspy^63^. Integrated XRF peak intensities were extracted the spectra at each point. For the Br:Pb maps, the Br K_α_ and Pb L_α_ peak intensities were used. The ratios shown are a direct ratio of these peak intensities, not a quantitative measure of Br and Pb concentrations. Although the maps were not corrected for self-absorption, we performed checks to ensure that the self-absorption of the perovskite and of the other layers in the device stacks were not substantially skewing the data. We performed complete self-absorption correction using the python package PyMca^64^. This complete correction accounts for the thickness and self-absorption of each layer in the stack, the incident and outcoupled angles of the X-ray beams, the path length of the X-rays through air after fluorescence, which can absorb low-energy X-rays, and the material and thickness of the detector. We see negligible differences in the spatial variation of the chemical composition after having applied the self-absorption correction (Supplementary Fig. 83).

### Reporting summary

Further information on research design is available in the Nature Portfolio Reporting Summary linked to this article.

## Supplementary information

Supplementary Information Supplementary Notes 1–7 and Figs. 1–83.

Reporting Summary

## Data Availability

The data and codes supporting the main text figures in this work are available at the repository at 10.17863/CAM.111849.
