# Supplementary material for: The impact of interfacial quality and nanoscale performance disorder on the stability of alloyed perovskite solar cells
Source: Nat Energy. 2024 Oct 30;10(1):66–76. doi: 10.1038/s41560-024-01660-1 (PMC11774756; doi:10.1038/s41560-024-01660-1)
Supplement: Supplementary file 1 — Supplementary Notes 1–7 and Figs. 1–83. [file 41560_2024_1660_MOESM1_ESM.pdf]

nature energy

Supplementary information https://doi.org/10.1038/s41560-024-01660-1

The impact of interfacial quality and nanoscale performance disorder on the stability of alloyed perovskite solar cells

In the format provided by the authors and unedited

This pdf file contains:

Supplementary Notes 1-7

Supplementary Figures 1-83

Table of Contents

Supplementary Note 1: Hyperspectral Data Analysis	2-3
Supplementary Note 2: Voltage-Dependent Photoluminescence Data Analysis	4-15
Supplementary Note 3: Experimental Limitations of Voltage Dependent PL	15-20
Supplementary Note 4: Image Registration	20
Supplementary Note 5: Compositional Correlations	20-26
Supplementary Note 6: Wrinkle Correlations	27-38
Supplementary Note 7: Hysteresis Spatial Variations	39-45
Supplementary Figures	46-85
Supplementary References	86-87

Supplementary Note 1: Hyperspectral Data Analysis

Quasi-Fermi level splittings were determined by fitting absolute-intensity PL spectra with the generalised Planck's law[1,2] using full peak fitting and models of below-bandgap absorption developed by Katahara and Hillhouse[3,4]. We have previously used this method to extract QFLS from hyperspectral maps of perovskites successfully[5]. The absolute intensity of photoluminescence ($I_{PL}(E)$) can be modelled as the product of the absorptance spectrum of the material, the photon density of states ($\rho(E)$) and a Bose-Einstein ($f_{BE}(E)$) occupation function with a finite quasi-Fermi-level splitting ($\Delta\mu$) as:

$$I_{PL}(E) = \rho(E) \times f_{BE}(E) \times a(E)$$

In the absence of large doping and solar relevant carrier densities, one can ignore occupation corrections to the absorptance spectra and approximate f_{BE} as a Boltzmann type occupation function giving the following expression for I_{PL}:

$$I_{PL}(E) = \frac{2\pi E^2}{h^3 c^2} \times a(E) \times exp\left(-\frac{E - \Delta\mu}{kT}\right)$$

The approach by Katahara and Hillhouse involves a model for the absorption coefficient to fit the entire peak to extract $\Delta\mu$. This involves a convolution of an above bandgap square root density of states type absorption coefficient dependence:

$$\alpha = \alpha_0 \sqrt{E - E_g}$$

α_0 is a parameter that depends on the oscillator strength of the material and E_g is the bandgap, and a below bandgap exponential tail absorption coefficient:

$$\alpha \propto \exp\left(\frac{E_g - E}{\gamma}\right)^{\theta}$$

Where θ is the power of the exponential tail and γ is its characteristic energy broadening (the Urbach energy when θ is 1). The convolution integral looks as follows:

$$\alpha(\varepsilon) = \frac{\alpha_0\sqrt{\gamma}}{2\Gamma\left(1 + \frac{1}{\theta}\right)} \int_{-\infty}^{\Delta\varepsilon} exp\left(-|\Delta\varepsilon'|^{\theta}\right)\sqrt{\Delta\varepsilon - \Delta\varepsilon'}\, d\Delta\varepsilon'$$

where Γ is the gamma function Rather than explicitly evaluating this convolution at each point, an approximation can be made with the assistance of a lookup table provided by Braly et al.[4] where they have explicitly evaluated the convolution integral $G(\frac{E-E_g}{\gamma},\theta)$ for a range of parameters. The resulting approximation for the absorption coefficient is:

$$\alpha = \alpha_0\sqrt{\gamma}G(\frac{E-E_g}{\gamma},\theta)$$

And substituting this expression into the above expression for I_{PL} assuming a Beer-Lambert type exponential relationship between a(E) and α(E) and film thickness d:

$$I_{PL}(E) = \frac{2\pi E^2}{h^3c^2} \times (1 - \exp\left(-\alpha_0 d\sqrt{\gamma}G\left(\frac{E-E_g}{\gamma},\theta\right)\right)) \times exp\left(-\frac{E-\Delta\mu}{kT}\right)$$

$\alpha_0 d$ was fixed at 5 as is commonplace for perovskites and has little impact on the resulted fits[4], and T was set to 300K. The remaining parameters were fit using a Levenberg-Marquardt, non-linear least squares fitting protocol implemented in Python. As is common for multi-variable, non-linear least squares fitting, suitable initial guesses are critical and so the average of each scan area was fit and manually inspected to ensure the quality of the fit before using these output parameters as the initial conditions for the automatic fitting procedure.

The spectral centre of mass for each point was calculated by interpolating the data along a uniform energy axis and then calculating as:

$$COM = \frac{\sum E_n \cdot I_{PL}(E)}{\sum I_{PL}(E)}$$

Supplementary Note 2: Voltage-Dependent Photoluminescence Data Analysis

The measurements produced a voltage dependent photoluminescence signal in both the reverse and forward scan directions. Voltage-dependent PL sweeps in the forward and reverse directions are normalised according to a protocol reported previously by Wagner et al.[6] The difference between the photoluminescence intensity of a point at open circuit $I_{PL}(V_{OC})$ and any voltage below it $I_{PL}(V)$ is related to the charge carrier extraction efficiency ($\Phi_{PL}(V)$) once normalised by $I_{PL}(V_{OC})$.

$$\Phi_{PL}(V) = \frac{I_{PL}(V_{OC}) - I_{PL}(V)}{I_{PL}(V_{OC})} = 1 - \frac{I_{PL}(V)}{I_{PL}(V_{OC})} \approx \frac{J(V)}{J_{gen}}$$

In order to demonstrate this relation, the aim is to relate the current flowing through a solar cell to Δμ which is probed by the photoluminescence. The current flowing through and ideal diode can be written as:

$$J(V) = J_{rec} - J_{gen} = J_0 \left(e^{\frac{eV}{k_B T}} - 1 \right) - J_{gen}$$

Where J_{rec} is the recombination current, J_{gen} is the generation current from the light-source and J_0 is the radiative saturation current and k_B is the Boltzmann constant. This diode equation assumes only radiative recombination, and no transport losses. If we assume that voltage is directly equivalent to Δμ, then this is the relationship needed. However, it has been shown that Δμ is not necessarily equivalent to V in perovskite solar cells, particularly those with low mobility charge transport layers[7,8]. Furthermore, recombination pathways beyond radiative recombination are known to occur in perovskite solar cells. To account for these pathways and to develop an explicit relationship between the current and Δμ, we first begin with a charge carrier recombination rate equation for an intrinsic semiconductor with rate constants for Shockley-Read-Hall recombination (k_1), radiative bimolecular (k_2) and Auger (k_3):

$$\frac{dn}{dt} = -k_1 n - k_2 n^2 - k_3 n^3$$

Writing the recombination rate equation in this form implies an intrinsic semiconductor with equal concentrations of electrons and holes which share non-radiative lifetimes and Auger rate constants. The carrier density can be related to Δμ with the relation $n^2 = n_i^2 \exp\left(\frac{\Delta\mu}{k_B T}\right)$ where n_i is the intrinsic carrier density. Subbing in for the carrier density we obtain:

$$\frac{dn}{dt} = -k_1 n_i e^{\frac{\Delta\mu}{2k_BT}} - k_2 n_i^2 e^{\frac{\Delta\mu}{k_BT}} - k_3 n_i^3 e^{3\Delta\mu/2k_BT}$$

J_{rec} is found by integrating this recombination rate across the active layer of the device. Assuming a thickness invariant Δμ and corresponding recombination rate through a device of thickness d, this gives:

$$J_{rec} = qd\left(-k_1 n_i e^{\frac{\Delta\mu}{2k_BT}} - k_2 n_i^2 e^{\frac{\Delta\mu}{k_BT}} - k_3 n_i^3 e^{\frac{3\Delta\mu}{2k_BT}}\right) \approx J_0\left(e^{\frac{\Delta\mu}{nk_BT}} - 1\right)$$

Where *n* is the ideality factor which accounts for the dominant recombination mechanism. For an ideal diode undergoing only radiative recombination, n=1, is above 1 for recombination mechanisms involving monomolecular processes such as Shockley-Read-Hall recombination and below one for Auger processes. The ideality factor is typically between 1 and 2 for perovskites[9]. Finally, we obtain an ideal diode like equation relating the current to Δμ:

$$J(\Delta\mu) = J_{rec} - J_{gen} = J_0\left(e^{\frac{eV}{nk_BT}} - 1\right) - J_{gen} \approx J_0\left(e^{\frac{\Delta\mu}{nk_BT}}\right) - J_{gen}$$

Next returning to the original expression for $\Phi_{PL}(V)$, by noting from the ideal Planck law written above that the PL intensity can be written as the product of the absorptance, the photon density of states and an exponential factor depending on Δμ, $\Phi_{PL}(V)$ can be written as:

$$\Phi_{PL}(V) = 1 - \frac{a \cdot \rho \cdot e^{\frac{\Delta\mu_V - E}{k_BT}}}{a \cdot \rho \cdot e^{\frac{\Delta\mu_{OC} - E}{k_BT}}} = 1 - \frac{e^{\frac{\Delta\mu_V}{k_BT}}}{e^{\frac{\Delta\mu_{OC}}{k_BT}}}$$

When the diode equation is at open circuit, no current flows and so we get an expression:

$$e^{\frac{\Delta\mu_{OC}}{k_BT}} = \frac{J_{gen}}{J_0}$$

And so $\Phi_{PL}(V)$ can be written as:

$$\Phi_{PL}(V) = 1 - \frac{J_0}{J_{gen}} \cdot e^{\frac{\Delta\mu_V}{k_BT}}$$

This expression is equal to the current for the diode equation with ideality factor of 1 normalised by the generation current and so:

$$\Phi_{PL}(V) \approx \frac{J(V)}{J_{gen}}$$

Normalising the voltage-dependent photoluminescence signal in this way gives you an approximate value for the ratio of the extracted current at that voltage (J(V)) to the total generated current at that point (J_{gen}). This means that if the photoluminescence drops from a finite value at open circuit to 0 at short circuit, all carriers generated by the excitation source are interpreted as being extracted. Any finite luminescence at 0 V implies incomplete charge carrier extraction as carriers are still recombining radiatively within the active layer and there remains a finite quasi-Fermi level splitting. In this normalisation scheme, the radiative recombination acts as a proxy for the total recombination. This relies on the assumption that both the radiative and total recombination scale in the same exponential way with voltage and in this case, an ideality factor of 1. When the ideality factor deviates from one, the expression is no longer exact and so will slightly over or underestimate the extracted current fraction.

To test the assumptions of the model as above, we ran SCAPS simulations[10] and varied an array of parameters to understand under what conditions the model is valid. As above, in the ideal diode case with no non-radiative losses and no transport losses, the relationship between radiative and total recombination is exact. We then simulated an idealised p-i-n perovskite solar cell with no non-radiative recombination losses and low mobility organic transport layers with the following material parameters included in Supplementary Table 1.

Supplementary Table 1: Bulk material parameters used in SCAPS drift diffusion simulations.

	**HTL (PTAA/SAM)**	**Perovskite**	**ETL (C_{60})**
Thickness (nm)	10	400	20
Bandgap (eV)	3	1.65	2.0
Electron Affinity (eV)	2.5	3.9	3.9
Dielectric Permittivity (relative)	3.5	22	5.0
CB Effective Density of States (cm^{-3})	1×10^{20}	2.2×10^{18}	1×10^{20}
VB Effective Density of States (cm^{-3})	1×10^{20}	2.2×10^{18}	1×10^{20}
Carrier thermal velocity ($cm\ s^{-1}$)	1×10^{7}	1×10^{7}	1×10^{7}
Electron mobility ($cm^2\ V^{-1}\ s^{-1}$)	1×10^{-5}	20	1×10^{-2}
Hole mobility ($cm^2\ V^{-1}\ s^{-1}$)	1×10^{-4}	20	1×10^{-2}
Donor density (cm^{-3})	1×10^{5}	1×10^{10}	1×10^{5}
Acceptor density (cm^{-3})	1×10^{5}	1×10^{10}	1×10^{5}
Radiative recombination coefficient ($cm^3\ s^{-1}$)		6×10^{-11}	

	**ITO**	**Cu**
Work function (eV)	5.3	4.1
Majority carrier barrier height (eV)	0.2	0.2
Built in voltage (V)	1.2	

With the above parameters in place we simulated the effects of the addition of parasitic resistance losses on the relationship between radiative recombination and total recombination. For all simulations, solid lines represent the extracted current normalised by the generation current $J(V)/J_{gen}$ while the dashed lines correspond to $\Phi_{PL}(V)$. We first varied the impact of series resistance (R_S) from 0 to 50 Ω cm^2, a wide range from an ideal solar cell to one extremely limited by this loss pathway. The results are plotted in Supplementary Figure 1. There is still an exact overlay between the two metrics over the range of varied parameters showing that series resistance introduces no appreciable errors.

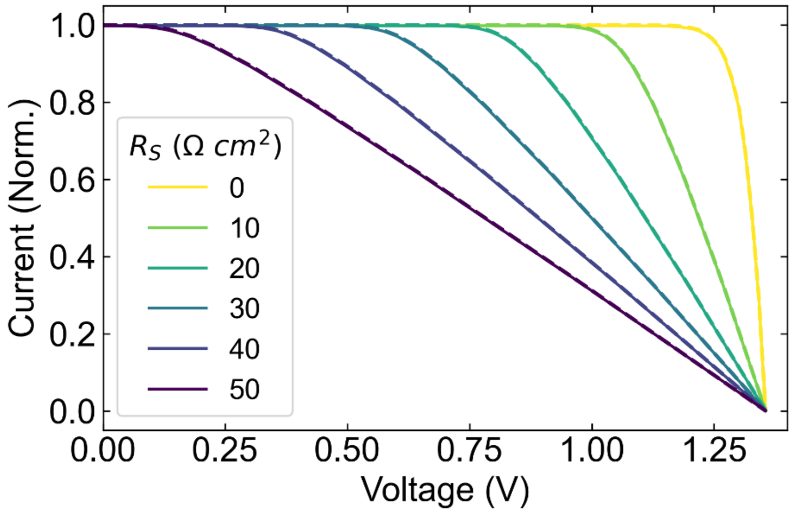

Supplementary Figure 1. Impact of series resistance (R_s) on relationship between $J(V)/J_{gen}$ (solid line) and $\Phi_{PL}(V)$ (dashed line).

By contrast, the effect of low shunt resistance, as shown in Supplementary Figure 2 does introduce a large error. We varied the shunt resistance (R_{sh}) from 200-1000 Ω cm^2 which corresponds to a significantly shunted device beyond what we see in our own devices to highlight this point. In a case with low shunt resistance, current simply bypasses the diode, meaning that electrical current and radiative recombination currents can become disconnected from one another. In cases where devices exhibit low shunt resistance, it should be noted that the relationship between $J(V)/J_{gen}$ and $\Phi_{PL}(V)$ can deviate significantly here. Across the range R_{sh} simulated, $\Phi_{PL}(V)$ does not appreciably change while the electrical curve changes considerably.

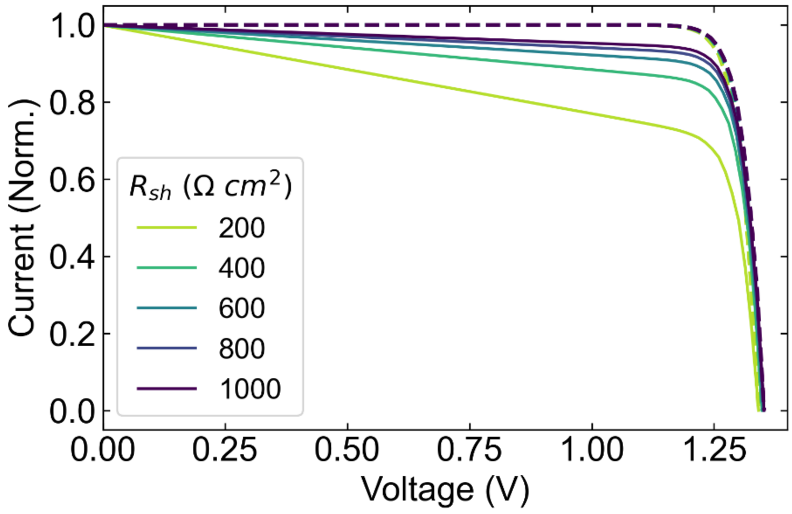

Supplementary Figure 2. Impact of shunt resistance (R_s) on relationship between $J(V)/J_{gen}$ (solid line) and $\Phi_{PL}(V)$ (dashed line).

Next, the mobility (μ) of charge carriers in the perovskite was also varied to simulate one avenue of poor charge extraction. μ was varied between 1×10^{-3} - 20 cm^2 V^{-1} s^{-1} and the results shown in Supplementary Figure 3. The electron and hole mobilities were varied simultaneously so in each simulation, the number listed corresponds to both electron and hole mobilities. While the mobilities at the lower end of the range affected the electrical JV curves significantly, they also affected the radiative recombination in the same way, such that the relationship between optical and electrical JV curves is preserved.

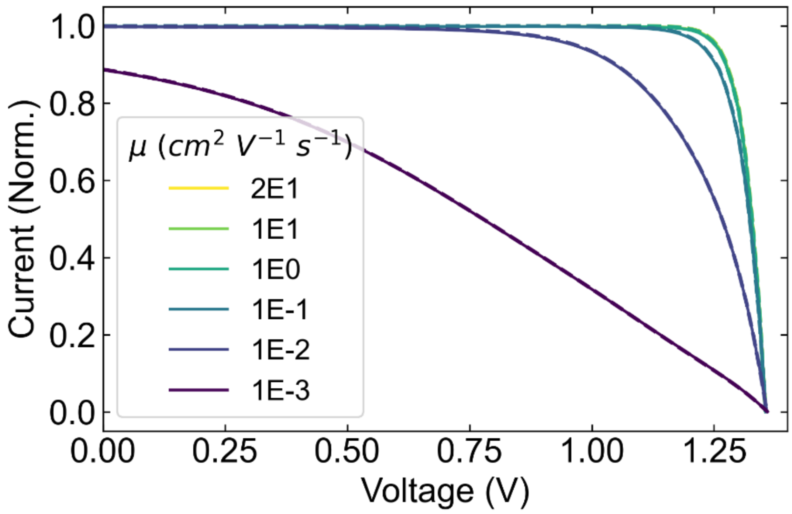

Supplementary Figure 3. Impact of carrier mobility (μ) on relationship between $J(V)/J_{gen}$ (solid line) and $\Phi_{PL}(V)$ (dashed line).

We then added in bulk non-radiative recombination in the perovskite through a defect state 0.6 eV above the valence band with electron and hole capture cross sections of 1×10^{-15} cm^2. The concentration of these defects was varied from $1\times10^{14} – 1\times10^{17}$ cm^{-3}, which corresponds to a range of non-radiative lifetimes from 1000-1 ns and the results shown in Supplementary Figure 4. In this case, the effective ideality factor of the diode is no longer one and as such, the relationship between $J(V)/J_{gen}$ and $\Phi_{PL}(V)$ is no longer exact. In this situation, the optical JV curve can overestimate the fill factor of the device with increasing error at shorter non-radiative lifetimes.

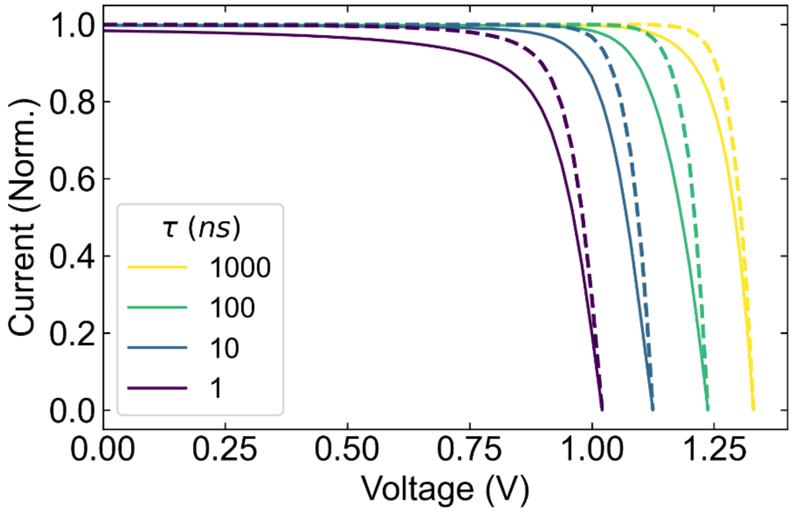

Supplementary Figure 4. Impact of non-radiative lifetime (τ) on relationship between $J(V)/J_{gen}$ (solid line) and $\Phi_{PL}(V)$ (dashed line).

Next the impact of interfacial recombination was investigated. All other non-radiative recombination pathways were removed and a defect was placed at the interface between the perovskite and C_{60} ETL. The concentration of the defect was varied between 10^{7}-10^{13} cm^{-2} which corresponds to surface recombination velocities of 10^{-2}-10^{4} cm s^{-1} and results shown in Supplementary Figure 5. Interestingly, while the non-radiative losses at this defect can still be substantial for large surface recombination velocities, the error introduced by this type of loss is actually reduced compared to the bulk non-radiative recombination.

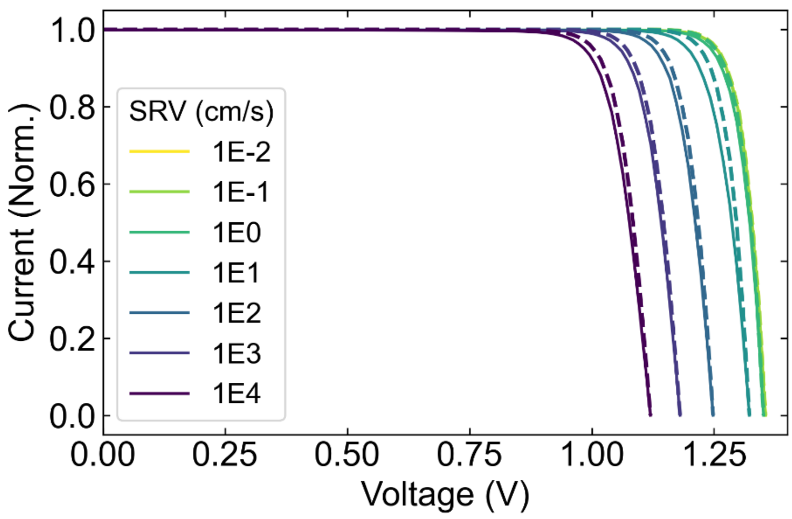

Supplementary Figure 5. Impact of surface recombination velocity (SRV) on relationship between $J(V)/J_{gen}$ (solid line) and $\Phi_{PL}(V)$ (dashed line).

We simulate a 'realistic' perovskite solar cell, incorporating bulk non-radiative recombination in the perovskite and both transport layers, as well as interfacial recombination losses. The complete list of parameters for bulk and interfacial defects are shown in Supplementary Table 2. These parameters are mirrored in the literature and guided by experimental observations of the bulk Shockley-Read-Hall recombination dominated lifetime of perovskites[11], carrier lifetimes in conjugated organics and fullerenes[12,13], surface recombination velocities at HTL and ETL interfaces[14], paying particular attention to our previous work showing that C_{60} is the more problematic interface[15]. Incorporating all of these recombination losses, we also vary the built in voltage by varying the work function difference between the ITO and copper electrodes from 1-1.2 V. Previous work has shown that for these systems that a built in voltage of at least 1.2 V is required to efficiently extract charges[16], so we wanted to see the effect that dipping below this threshold had on the optical-electrical relationship. The results are shown in Supplementary Figure 6. For the range of parameters over which our solar cells vary, the simulations suggest that the relationship between $J(V)/J_{gen}$ and $\Phi_{PL}(V)$ is a good approximation of the true form of the JV curve. For our experimental data, we tend to see a small systematic overestimation of the fill factor from the optical JV data compared to the electrical JV data, suggesting that bulk recombination is still playing an important role as a loss mechanism in our solar cells.

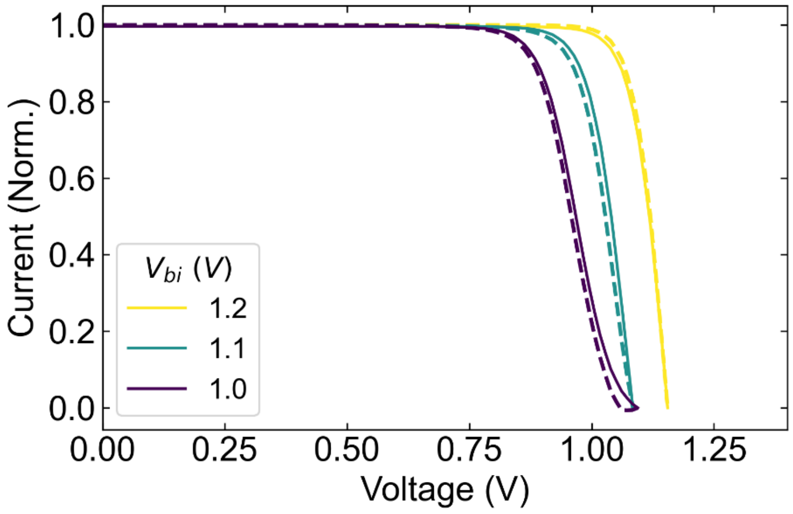

Supplementary Figure 6. Impact of built in potential on relationship between J(V)/J_{gen} (solid line) and Φ_{PL}(V) (dashed line) for a 'realistic' perovskite solar cell.

We finally note that for our solar cells, even at 0 V external bias, there is still a large Δμ or internal voltage within the solar cell that can be seen with luminescence as has recently been reported[8]. This has been attributed to the low mobilities and relative permittivities of the charge transport layers in p-i-n perovskite solar cells. In our simulations, plotting the perovskite thickness averaged Δμ versus applied voltage for our 'realistic' perovskite solar cell allows us to reproduce this result well as shown in Supplementary Figure 7 where the internal and external voltage are dramatically different, particularly at voltages close to short circuit. This is a loss mechanism that will need to be addressed for p-i-n perovskite solar cells to approach radiative efficiency limits.

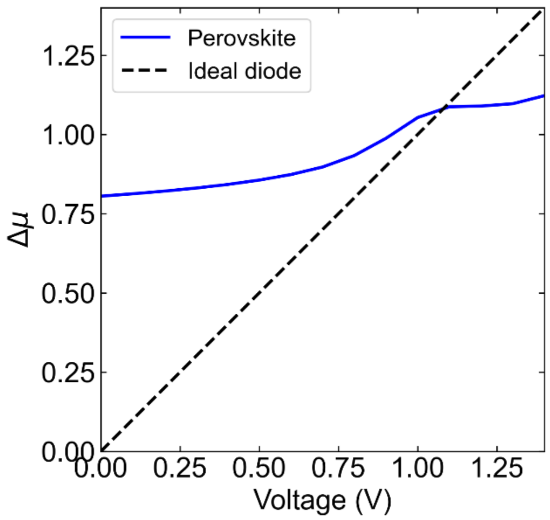

Supplementary Figure 7: Δμ versus voltage for ideal diode solar cell (dashed line) and simulated perovskite solar cell (solid line) showing large finite Δμ at 0V.

Supplementary Table 2: Parameters for defects used in 'realistic' simulation.

	HTL (PTAA/SAM)	Perovskite	ETL (C_{60})
Defect type	Neutral	Neutral	Neutral
Electron capture cross section (cm^2)	1×10^{-15}	1×10^{-15}	1×10^{-15}
Hole capture cross section (cm^2)	1×10^{-15}	1×10^{-15}	1×10^{-15}
Energetic distribution	Single	Single	Single
Energy above valence band (eV)	0.6	0.6	0.6
Trap density (cm^{-3})	1×10^{17}	2.2×10^{14}	1×10^{17}
Effective non-radiative lifetime (ns)	1	450	1

	HTL/Perovskite Interface	Perovskite/ETL Interface
Defect type	Neutral	Neutral
Electron capture cross section (cm^2)	1×10^{-16}	1×10^{-16}
Hole capture cross section (cm^2)	1×10^{-16}	1×10^{-16}
Energetic distribution	Single	Single
Energy above highest valence band (eV)	0.6	0.6
Trap density (cm^{-2})	1×10^{11}	2.2×10^{12}
Surface recombination velocity (cm/s)	100	2200

After the voltage dependent PL maps have been measured, to process the data, we first employ a principle component analysis as implemented in Hyperspy[17] and then denoised the data by reconstructing the dataset based on the first 10 principle components. In all cases, the first 10 components accounted for the vast majority of the variance in the dataset. Then for the reverse scan, the first image at open circuit was used as the normalisation factor, whereas the last image was used for the forward scan. In some cases, some light soaking resulting in photobrightening could be observed during the measurement in some samples, this results in a shifting of the forward scan up in the forward direction, resulting in an apparent hysteresis in the short circuit current that is not observed in the electrical JV data. The implications of this light soaking on actual device performance are not clear but it is worthy of note.

After the denoising and normalisation, the figures of merit were extracted locally in an analogous way to conventional JV data. $\Phi_{PL}(0)$ is the short circuit current extraction ratio, the macroscopic V_{OC} is implicitly assumed to be uniform across the device in this scheme, the maximum pseudo-power is found by finding the maximum value of the product $\Phi_{PL}(V)\times V$, and the fill factor is obtained by dividing this value by the product of the short circuit current extraction efficiency times the open circuit voltage:

$$FF_{PL} = \frac{\Phi_{PL}(V_{MPP}) \times V_{MPP}}{\Phi_{PL}(0) \times V_{OC}}$$

Doing these calculations at each point produces figure of merit maps across the measured area of the device.

Supplementary Note 3: Experimental Limitations of Voltage Dependent PL

Moving on from drift-diffusion simulations, we now discuss some experimentally determined limitations of the voltage dependent PL technique which should be taken into account if replicating this technique. The first key point is that a significant fraction of the active area of the device must be illuminated by the light source. As was also observed by Wagner et al.[6], unlike in a bare perovskite film which is not particularly conductive laterally, once a perovskite is sandwiched between conductive contacts, only illuminating a small spot can cause issues with the measurement. At open circuit, upon illumination of a small spot, there is now a high carrier density in this region – carriers can be extracted at the contacts, diffuse laterally away from the illumination spot and re-inject themselves into the perovskite away from the illuminated spot. This reduces the PL intensity measured at open circuit. This effect becomes less important as the cell is biased towards short circuit as the carrier density gradient is reduced so the PL at J_{SC} is similar to the homogeneous illumination case. The result is that the observed PL quenching is much lower than one might expect for a high performance device. An example of an early measurement where we were using a "wide-field" illumination that only illuminated a small fraction of the active area is shown in Supplementary Figure 8. Short circuit charge extraction efficiencies were routinely measured to be on the order of 0.3, far below what one would expect for a well behaved solar cell and the optical JV curves did not match well with the shape of the electrically measured curve. External illumination where the entire active area was illuminated was critical to reproduce the quenching levels expected.

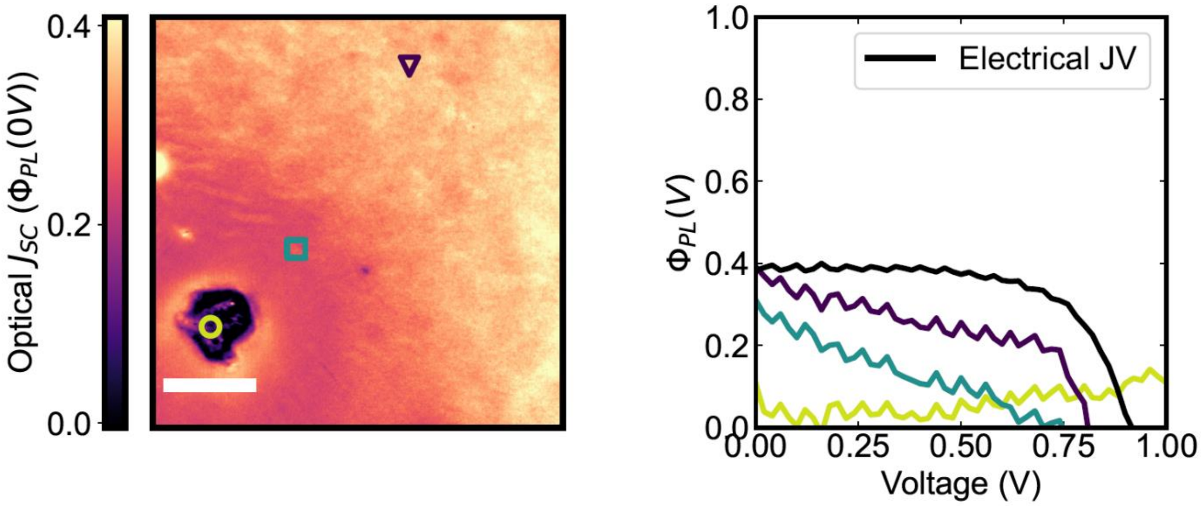

Supplementary Figure 8: Voltage dependent PL microscopy with small area illumination. a) Optical short circuit extraction efficiency map (ΦPL(0V)) of triple cation double halide perovskite solar cell. b) Optical JV curves of the regions marked in a and the normalised, electrically measured macroscopic JV curve measured simultaneously (black line). Scalebar is 75 µm.

It is next important to compare experimentally measured curves to understand the strength and limitations of the technique. It is worth stating first that if optical JV curves are extracted from a small region of the active area, the electrical and optically measured JV curves may not match up due to spatial heterogeneities in the behaviour of the device. In situations where the solar cell initially behaves approximately like a diode, the optical and electrical curves match up well as is shown in Figure 1 of the main text. Even in situations where the solar cell is not showing ideal behaviour and shows reduced fill factor as shown in Supplementary Figure 9.

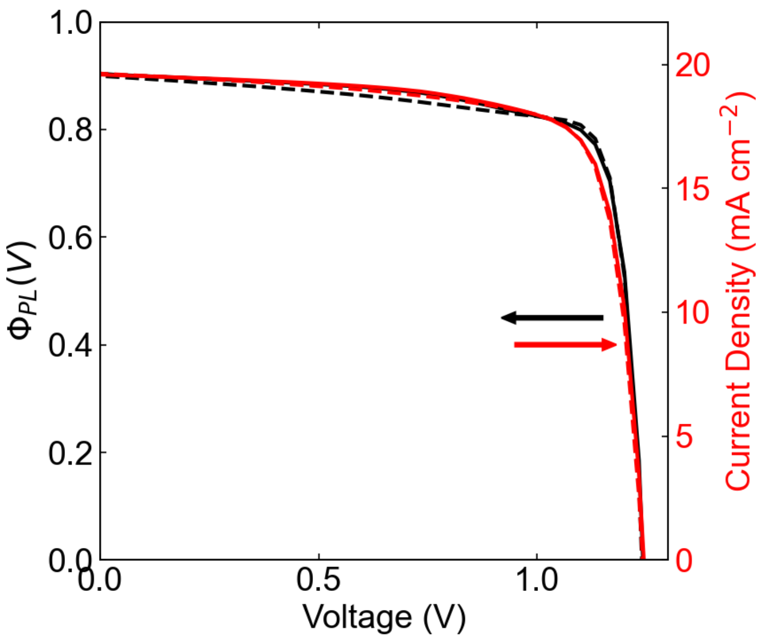

Supplementary Figure 9. Comparison of the optically extracted JV curve (black) and the electrical JV curve (red) for a 2PACz/TCTH sample passivated with LiF. Solid lines are reverse voltage scans, dashed lines are forward scans. Coloured arrows point to their respective y-axes.

In a device where the initial JV characteristic is relatively well behaved, and the JV curve after stress testing is similarly well behaved, the optical and electrical curves continue to show relatively strong agreement. Supplementary Figure 10 shows the JV curves extracted from the same 2PACz/TCTH sample before and after operational stress testing. There is a small offset between the current values which may be explained simply by lateral heterogeneity in the sample, but the shape of the JV curves is close to identical.

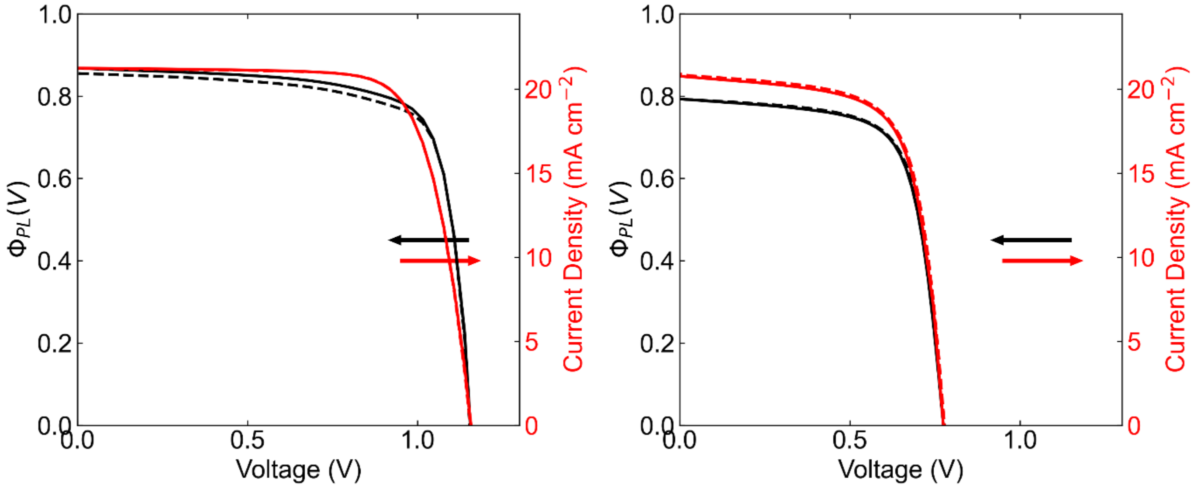

Supplementary Figure 10. Comparison of the optically extracted JV curve (black) and the electrical JV curve (red) for a 2PACz/TCTH sample a) before and b) after operational stress

testing. Solid lines are reverse voltage scans, dashed lines are forward scans. Coloured arrows point to their respective y-axes.

In situations where the device is already more disordered as is the case in the DCTH device shown in Figure 3 of the paper and in Supplementary Figure 11, the fresh device optical JV curve shows a larger hysteresis than in the electrical JV curve – this is in reference to the optical hysteresis mentioned in the main text of the paper and relates to light soaking of the device during the measurement. If the devices emission yield changes during the scan, this can be a source of error that is not reflected in the electrical JV curve so care should be taken if the device is transiently changing. After operational stress, there is a larger degradation of the sample and the optical JV curve somewhat over-estimates the short circuit current of the device, this appears to be a somewhat general trend for devices that show considerable degradation – in particular when mobile ions are present. This over-estimation is also present in the 2PACz/DCDH device that suffered from substantial edge effects and hysteresis as shown in Supplementary Figure 12.

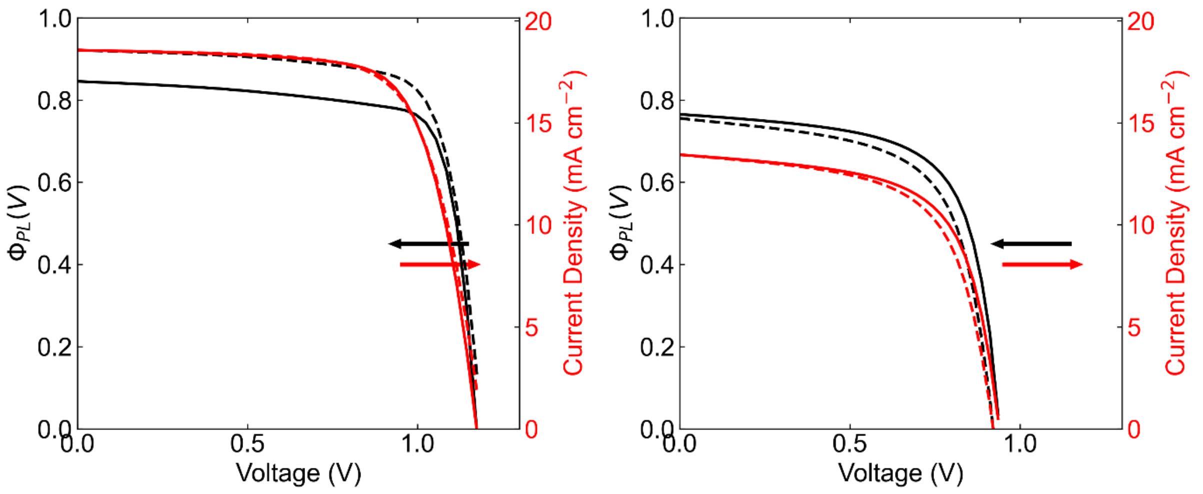

Supplementary Figure 11. Comparison of the optically extracted JV curve (black) and the electrical JV curve (red) for a 2PACz/TCTH sample a) before and b) after operational stress testing. Solid lines are reverse voltage scans, dashed lines are forward scans. Coloured arrows point to their respective y-axes.

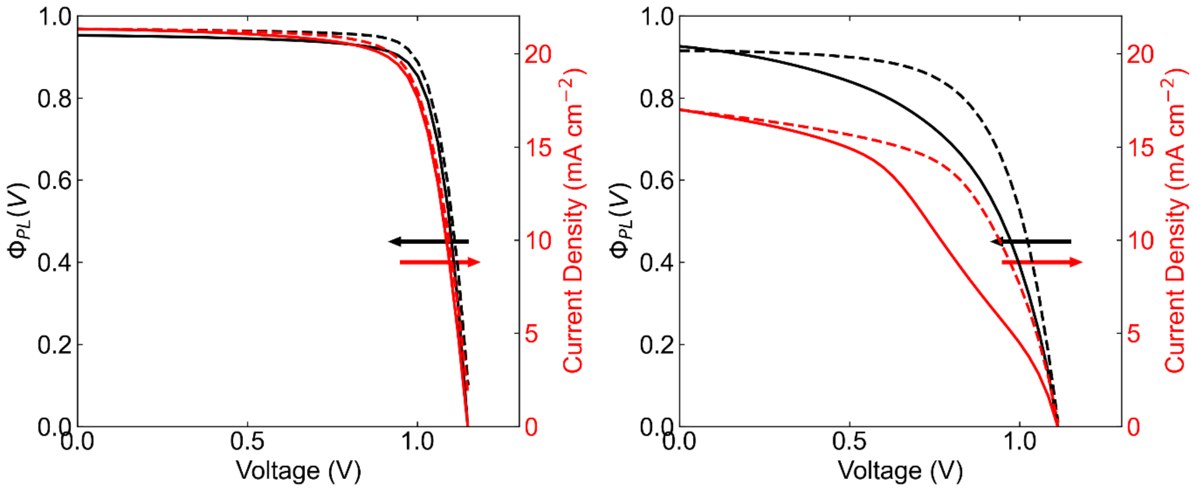

Supplementary Figure 12. Comparison of the optically extracted JV curve (black) and the electrical JV curve (red) for a 2PACz/DCDH sample a) before and b) after operational stress testing. Solid lines are reverse voltage scans, dashed lines are forward scans. Coloured arrows point to their respective y-axes.

The final case worth considering is that, as shown in the simulations, in the case where there is some shunting, the optical JV curve will over-estimate the fill factor of the device as seen in the PI passivated device in Supplementary Figure 13, so care should be taken to ensure that if the shunt resistance of the device is low, this is known in advance. After ageing, this device suffered catastrophic degradation. While this is also reflected in the optical JV curve, the short circuit current density is over-estimated as mentioned previously. Care must therefore be taken that in situations of the most serious degradation to account for this overestimation.

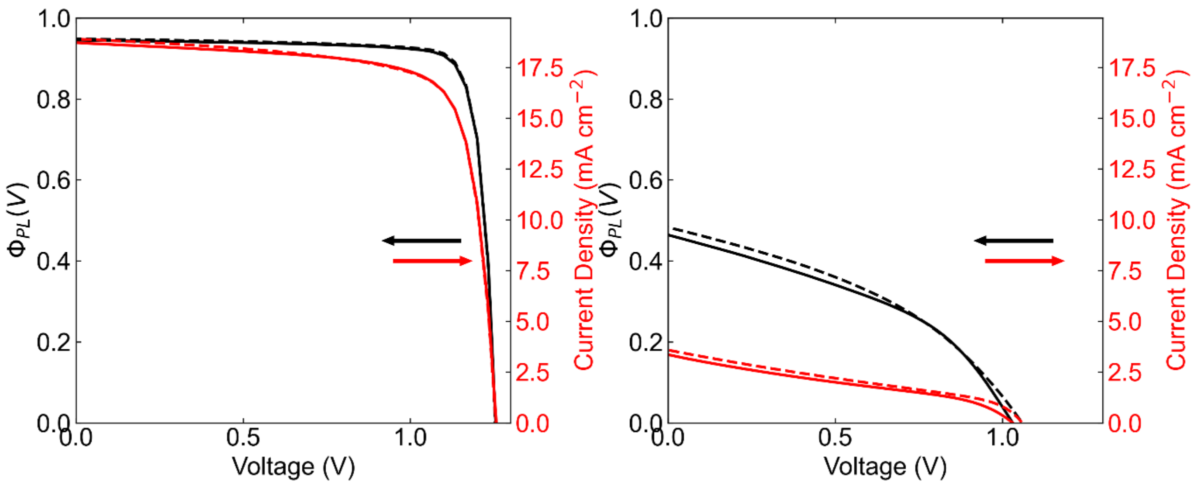

Supplementary Figure 13. Comparison of the optically extracted JV curve (black) and the electrical JV curve (red) for a 2PACz/TCTH device passivated with PI a) before and b) after operational stress testing. Solid lines are reverse voltage scans, dashed lines are forward scans. Coloured arrows point to their respective y-axes.

Supplementary Note 4: Image Registration

All samples were initially marked with a small fiducial scratch produced by a diamond tipped scribe on the glass side. This was used as a coarse alignment by initially focusing on the top of the glass substrate to find the region. After finding the scratch, the focus was brought through the glass to the perovskite where a distinctive marking or imperfection was found. The combination of these two protocols allows reliable, rapid returns to the same area of the sample. Image registration between the fresh and aged datasets was performed using Advanced Normalization Tools (ANTs), an open-source and state-of-the-art medical image registration and segmentation toolkit[18]. ANTs provides a flexible and widely-used registration framework, with many different similarity metrics that can be used to tune registration performance[19]. Optical microscopy images were registered using an affine transformation with a squared intensity difference or mutual information similarity metric. nXRF to optical microscopy image registration primarily relied on the mutual information similarity metric for improved performance between different imaging modalities (see Python scripts for details).

Supplementary Note 5: Compositional Correlations

In order to investigate the extent to which local compositional variation correlates with local device performance parameters we performed pixel-by-pixel spatial correlation analysis between the compositional information measured by nXRF and a wide array of optoelectronic figures of merit measured by the optical microscopes across the device space described in the main text. In Supplementary Figure 14, we show 2-dimensional histograms with the Br:Pb ratio on the x-axis and the optoelectronic figure of merit on the y-axis. The color map represents the density of points contained in that particular bin while the dotted black line represent the mean value of the optoelectronic figure of merit for that value of the composition. Superimposed on each histogram is the Spearman's rank correlation coefficient and associated p-value which assesses whether there is a monotonic relationship between the two variables of interest and the strength of said relationship (rather than assuming a linear relationship in the case of Pearson's coefficient). Supplementary Figure 14 shows the case for a pristine double-cation

double-halide (DCDH) perovskite fabricated on the 2PACz HTL. Supplementary Figure 14 a shows a strong anticorrelation between open circuit photoluminescence (PL) intensity and Bromine content as we previously qualitatively commented on in the manuscript, while there is a strong positive correlation between the PL centre of mass energy (COM) and Br:Pb. These two opposing trends effectively cancel one another out in the quasi-Fermi level splitting (Δμ) which shows effectively no dependence on the Br:Pb, suggesting that a combination of lower trap densities and local carrier funnelling cancel out any voltage penalty induced by the lower bandgap. For the optical Jsc, PCE and fill factor shown in Supplementary Figure 14 b-d, there are all very weak relationships between composition and electrical performance suggesting that for the pristine DCDH sample, the compositional variation has a relatively small effect. The changes that are present are most likely attributable to the wrinkles which we will go into much greater detail in Supplementary Note 6.

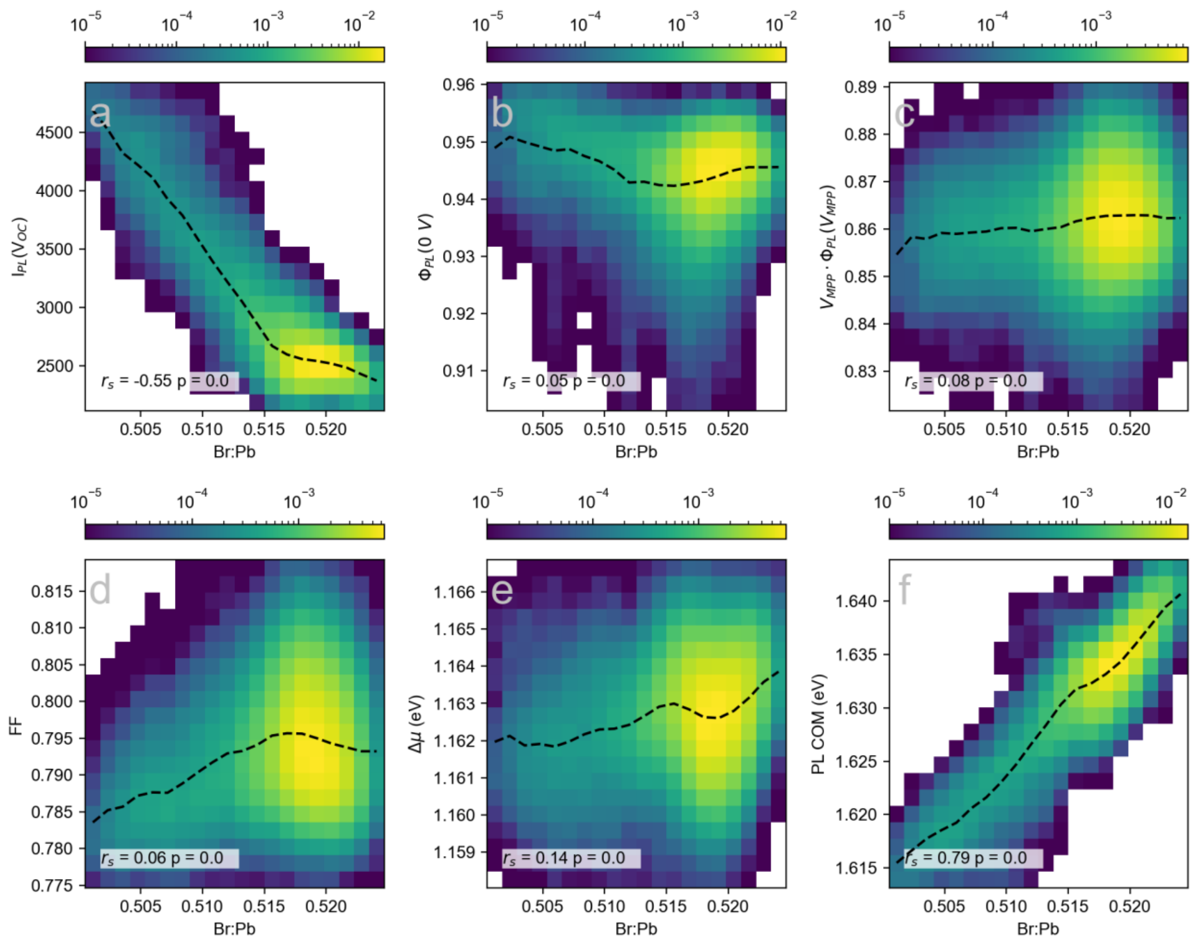

Supplementary Figure 14: 2-dimensional histograms of the spatial correlations between the Br:Pb content of a pristine 2PACz/DCDH solar cell and a) open circuit PL intensity ($I_{PL}(V_{OC})$), b) short circuit current extraction efficiency ($\Phi_{PL}(0\ V)$), c) optical power conversion efficiency,

d) optical fill factor, e) Δμ and f) PL centre of mass. Overlaid over each map is the Spearman's rank correlation coefficient between the two variables and the related p-value (two-sided test). The black dashed curves represent the mean value of the FOM of interest for each compositional window in x and are guides to the eye.

By comparison, compositional correlations of a stress tested equivalent of the same sample type (2PACz/DCDH) is included in Supplementary Figure 15. The same correlation/anticorrelation between composition and PL COM/$I_{PL}(V_{OC})$ is still observed. However, there is now a small increase in Δμ with Br:Pb suggesting that the low Br regions may have degraded slightly more than the other areas and this is also reflected in the optoelectronic figures of merit. However, the effect size is extremely small in Δμ and in the optical PCE where there is a few percent change across the Br:Pb range. In Supplementary Figure 37, one can see that in the optical PCE maps of the pristine sample, the wrinkles are quite visible, but in the operated samples, they are less evident as larger scale variations dominate the contrast. However, the behaviour of the 2PACz/DCDH is not entirely general across the sample space.

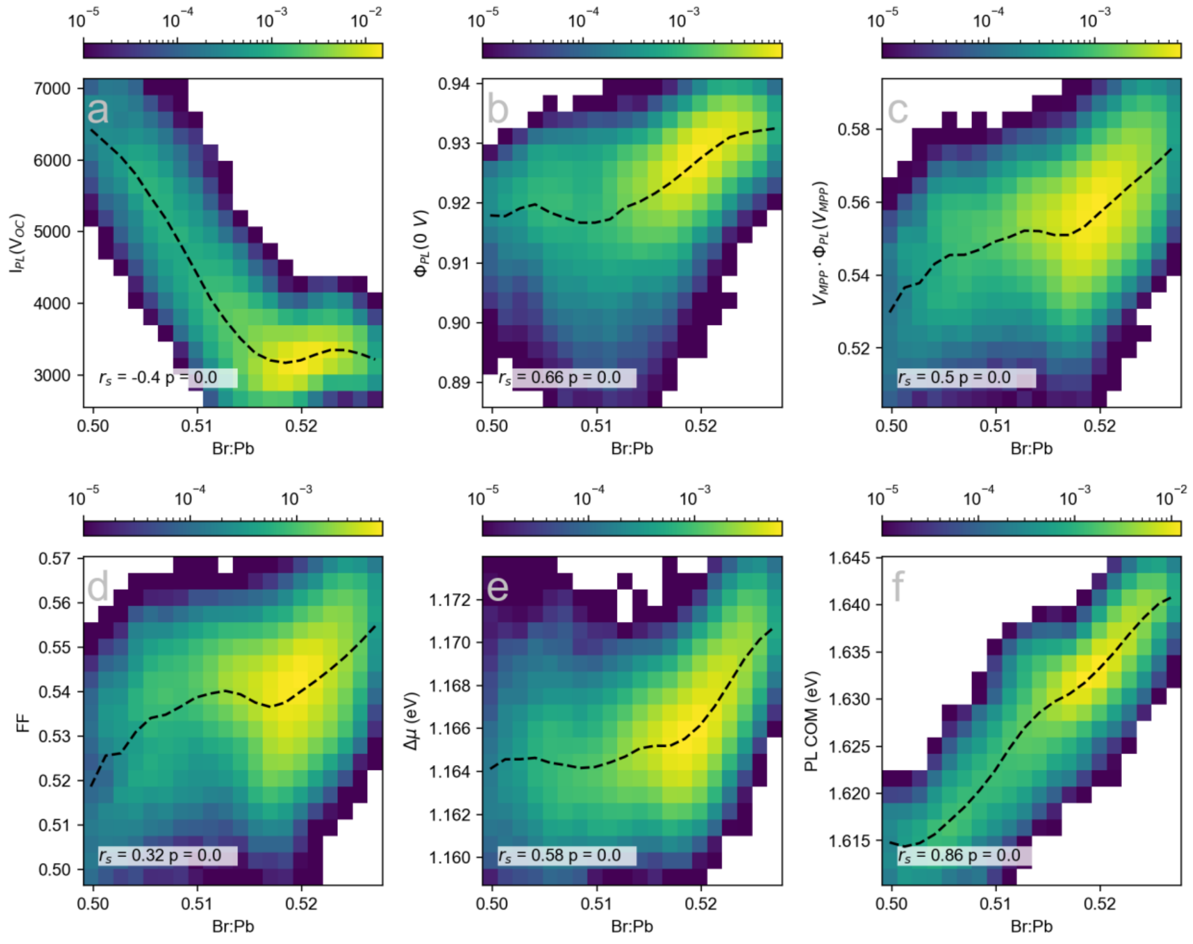

Supplementary Figure 15: 2-dimensional histograms of the spatial correlations between the Br:Pb content of a operationally stress tested 2PACz/DCDH solar cell and a) open circuit PL intensity ($I_{PL}(V_{OC})$), b) short circuit current extraction efficiency (Φ_{PL}(0 V)), c) optical power conversion efficiency, d) optical fill factor, e) $\Delta\mu$ and f) PL centre of mass. Overlaid over each map is the Spearman's rank correlation coefficient between the two variables and the related p-value (two-sided test). The black dashed curves represent the mean value of the FOM of interest for each compositional window in x and are guides to the eye.

The most extreme case of phase segregation (and therefore largest range of Br:Pb ratios observable which are most likely to detect the compositional limits at which losses may occur) is seen in the 2PACz/DCTH samples, particularly those after operation. This can be seen in Figure 3 and Supplementary Figures 51, 53 and 54. In the analysis of the fresh DCTH sample (Supplementary Figure 16), it is evident that there is now a significant correlation between the Br:Pb content and the optoelectronic figures of merit (in particular charge extraction efficiency and optical PCE) where there was not before, as is also qualitatively observable in Supplementary Figure 51 and 53. This implies that the low Br regions/wrinkles are negative

for performance, in particular charge extraction, in this device architecture. However, in the operationally stressed devices as shown in Supplementary Figure 17, there is the emergence of highly Br deficient, phase segregated regions with markedly different recombination and transport behaviour and the resulting spread of composition is much larger than in other sample types. Previously, the distribution of PL COM and the associated correlation with Br:Pb was effectively unimodal, however, the PL COM of these samples indicate two distinct populations with low Br: wrinkles and segregation. The segregated regions have an ever more pronounced impact on the PL COM (they are the lower band in the plot), and actually appear to be positive for the charge extraction relative to the surroundings. The overall statistical result suggests that above a threshold value of Br:Pb, there is very little effect of composition on optical PCE, however below this value, low Br actually improves the charge transport and therefore the performance.

Taken together, these results suggest that in pristine samples, there often appears to be a statistically significant positive correlation with small effect size between composition and optical PCE. However, there is additionally a Br threshold relative to its surroundings, below which, the sample is sufficiently segregated to have a large, positive effect on the charge extraction. This suggests that the phase segregated low bandgap regions are not the cause of current loss in wide bandgap solar cells, but it may indeed be the unsegregated or wide gap regions left behind.

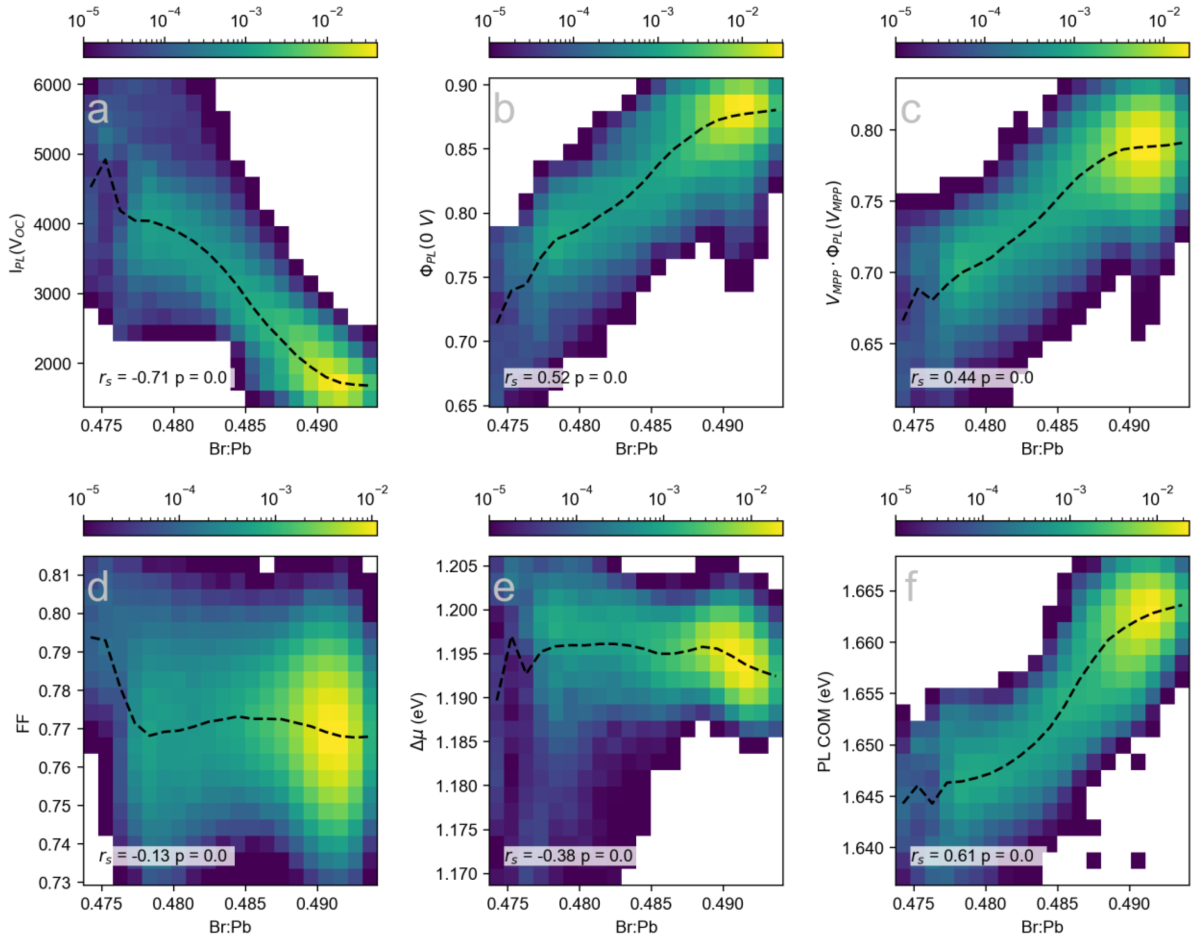

Supplementary Figure 16: 2-dimensional histograms of the spatial correlations between the Br:Pb content of a pristine 2PACz/DCTH solar cell and a) open circuit PL intensity ($I_{PL}(V_{OC})$), b) short circuit current extraction efficiency ($\Phi_{PL}(0\ V)$), c) optical power conversion efficiency, d) optical fill factor, e) $\Delta\mu$ and f) PL centre of mass. Overlaid over each map is the Spearman's rank correlation coefficient between the two variables and the related p-value (two-sided test). The black dashed curves represent the mean value of the FOM of interest for each compositional window in x and are guides to the eye.

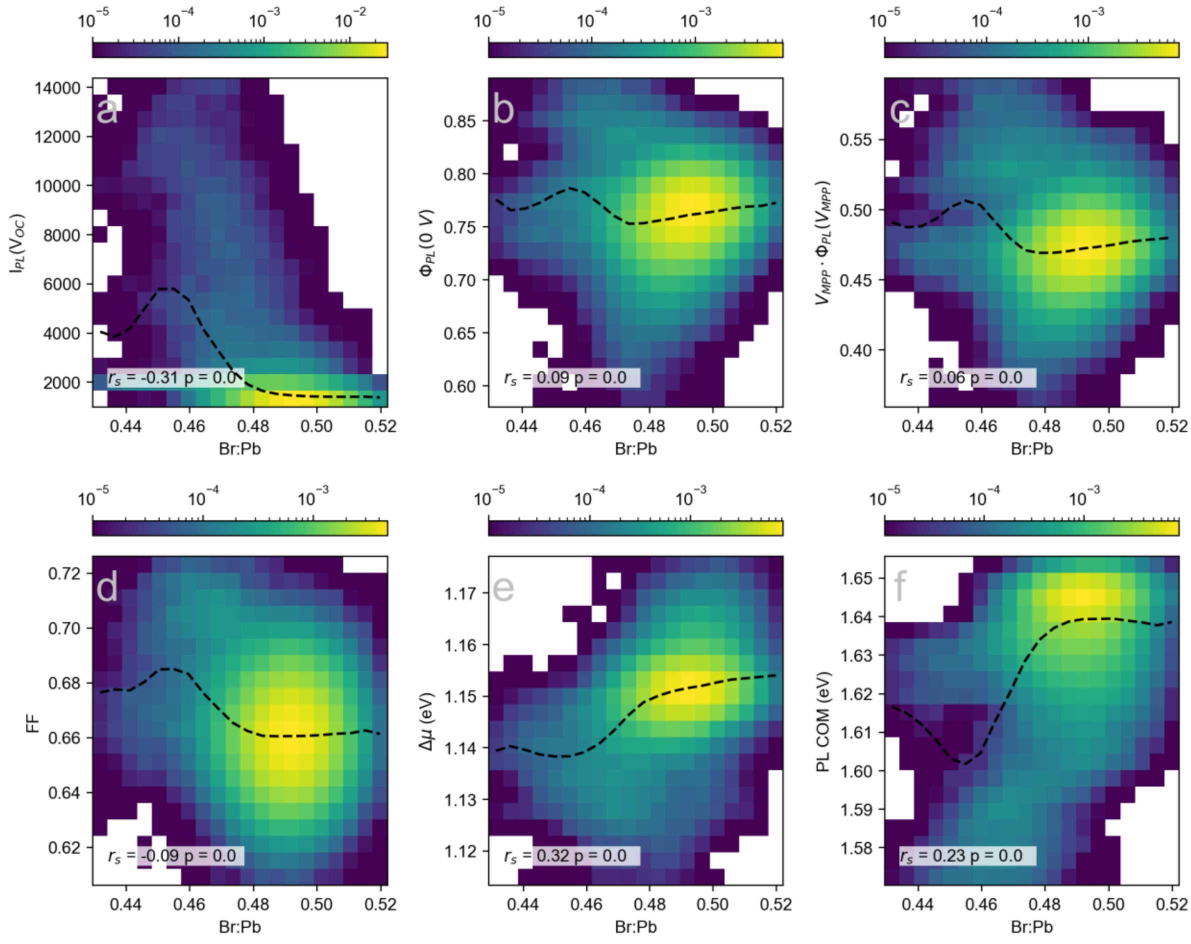

Supplementary Figure 17: 2-dimensional histograms of the spatial correlations between the Br:Pb content of an operationally stress tested 2PACz/DCTH solar cell and a) open circuit PL intensity ($I_{PL}(V_{OC})$), b) short circuit current extraction efficiency ($\Phi_{PL}(0\ V)$), c) optical power conversion efficiency, d) optical fill factor, e) $\Delta\mu$ and f) PL centre of mass. Overlaid over each map is the Spearman's rank correlation coefficient between the two variables and the related p-value (two-sided test). The black dashed curves represent the mean value of the FOM of interest for each compositional window in x and are guides to the eye.

Supplementary Note 6: Wrinkle Correlations

Much like compositional variation, a feature present throughout all of the perovskite films and devices we measured in this study is the presence of wrinkles that cover a significant fraction of the perovskite's area. The field has been aware of the presence of these wrinkles for some time[20], with the microscopy approach demonstrated here, we have the ability to comment directly on influence of wrinkles on local device figures of merit and stability.

The wrinkles are very apparent in bright-field optical reflectance images so we used these as the basis for a thresholding tool to produce a mask specifically for the wrinkles. We used the python package Pyclesperanto, a tool traditionally used for quantitative cell segmentation and analysis in a biological context. After a series of thresholding, filtering and segmentation steps, a mask can be reliably produced with statistical information about the size, shape and spatial distribution of each wrinkle, enabling us to perform a detailed quantitative analysis. An example of a bright field reflectance image of a 2PACz/DCDH device is shown in Supplementary Figure 18 a. The wrinkles very clearly appear as regions of low intensity in the reflected image. Their morphology may prevent coherent thin-film reflection effects such as interference and may waveguide light laterally into the device to reduce reflectance. The masked wrinkles from the reflectance image are shown in Supplementary Figure 18 b. Each individual wrinkle object is marked by a colour distinct from its neighbours. The distribution of different wrinkle areas, the total number of wrinkles and the total surface coverage of wrinkles across the area of interest is shown in Supplementary Figure 18 c. This sample is in line with the most wrinkled of the devices in this study showing a ~20% surface coverage depending on the location of measurement. The filtered wrinkle mask was either used as a positive or negative mask to filter only the regions at or away from the wrinkles. Equivalent masks and distributions were generated for all the measured devices.

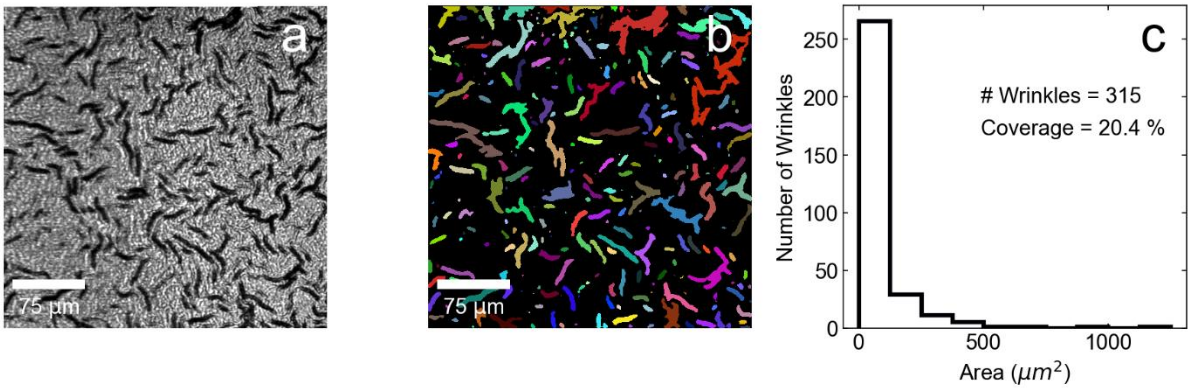

Supplementary Figure 18: a) Bright field reflectance image of 2PACz/DCDH solar cell. b) Filtered wrinkle mask from the reflectance image in a. c) Histogram of the size distribution of wrinkles in panel b.

Having established a robust protocol for masking regions where the wrinkles are present versus not present, we then apply them as a mask for the optoelectronic measurements. Supplementary Figure 19 a and b show the mean PL spectra and optical JV curves before and after the operational stress test on and off the wrinkled areas. The wrinkled areas show higher intensity, red shifted PL compared to the wrinkle free areas but both areas show effectively equivalent $\Delta\mu$. After operational stress, both areas have increased in intensity, with the spectral differences between both regions increasingly similar. The optical JV curves in this sample show effectively no difference in either the forward or reverse direction before or after operational stress – showing that while the wrinkles to mediate the emission spectrum, the charge extraction and stability is close to unaffected by the presence of the wrinkles in these samples. To show this more explicitly, we calculated the distributions of a range of device relevant optoelectronic parameters in the wrinkled versus non wrinkled cases. These are shown in Supplementary Figure 20. The open circuit PL follows a predictable trend and PL COM follows a similar trend to the compositional correlations above where the wrinkled areas have higher intensity, red-shifted emission. The other optoelectronic figures of merit follow suit, showing very close agreement both before and after operational stress testing, further solidifying our claim that the wrinkles are not seeds of high performance or degradation. One caveat to this dataset is the degradation was dominated by an edge effect which may blur out the effects we might otherwise with the wrinkles (see Supplementary Figures 41 and 42). We therefore include further analysis on regions and devices where this is not the case.

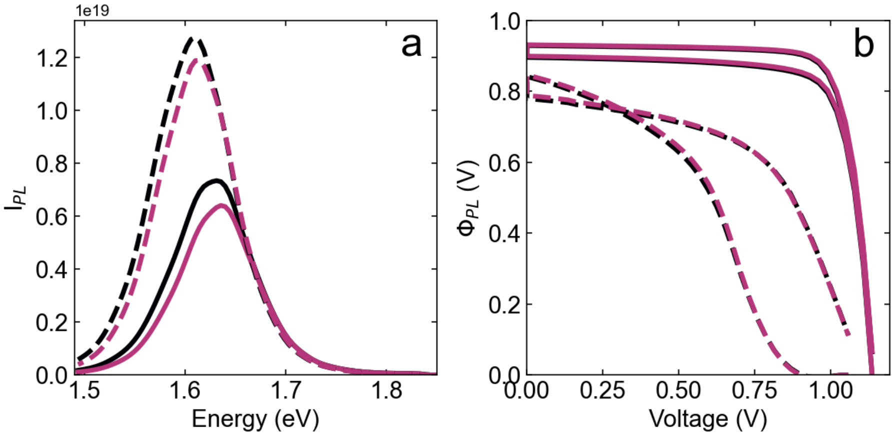

Supplementary Figure 19: a) Average PL spectra of non-wrinkled (red) and wrinkled (black) regions in a 2PACz/DCDH device before (solid lines) and after (dashed lines) operational stress. b) Mean optical JV curves of the same device and masked regions before and after operational stress.

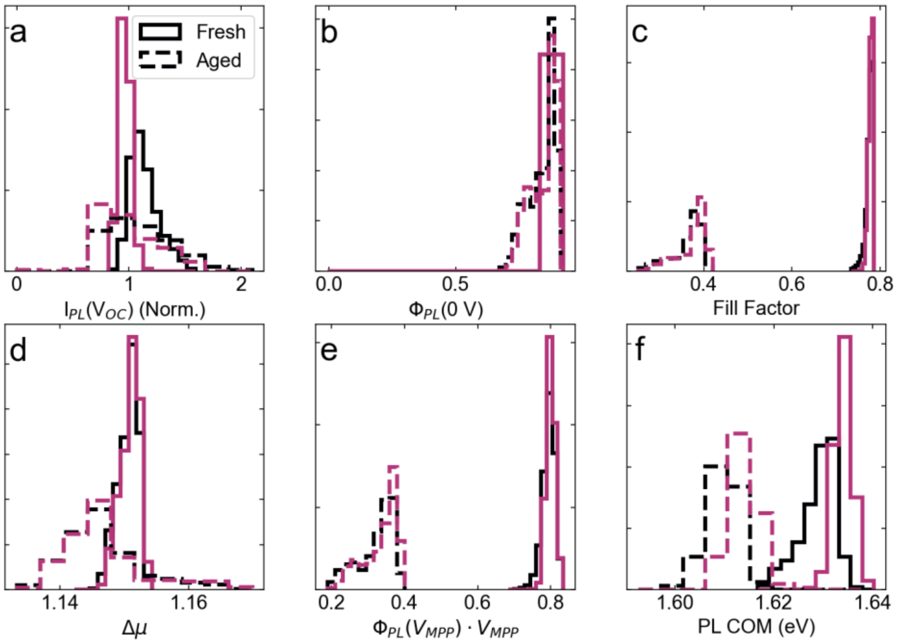

Supplementary Figure 20: Distributions of optoelectronic parameters on non-wrinkled (red) and wrinkled (black) regions before (solid) and after (dashed) operational stress of a 2PACz/DCDH device. a) open circuit PL intensity, b) short circuit optical charge extraction efficiency, c) optical fill factor, d) quasi-Fermi level splitting, e) optical power conversion efficiency and f) PL centre of mass.

We perform the same masking analysis on the complete range of devices measured beginning with 2PACz/DCTH. The size distribution and the surface coverage in these samples as shown in Supplementary Figure 21 is similar to the DCDH case as we previously claimed without quantitative evidence. The mean PL spectra before and after operational stress in Supplementary Figure 22 a show a consistent PL intensity increase and red shift in the wrinkled areas. However, after ageing both regions display quite a stark red shift and this is indicative of the fact that the highly phase segregated regions appear to form on both the wrinkled and non wrinkled regions – as opposed to their growth being preferentially seeded by one versus another. The optical JV curves in the pristine sample show that the wrinkles exhibit worse optical charge extraction efficiency and fill factor, but this variation disappears after operational stress where the performance of the wrinkles has homogenised with the rest of the sample (Supplementary Figure 22 b), and the only relevant deviation from the norm is the highly segregated regions which show reduced Δμ and improved optical extraction efficiency. This is further highlighted in Supplementary Figure 23 which show this homogenisation of the properties of the wrinkled and non-wrinkled areas after operational stress.

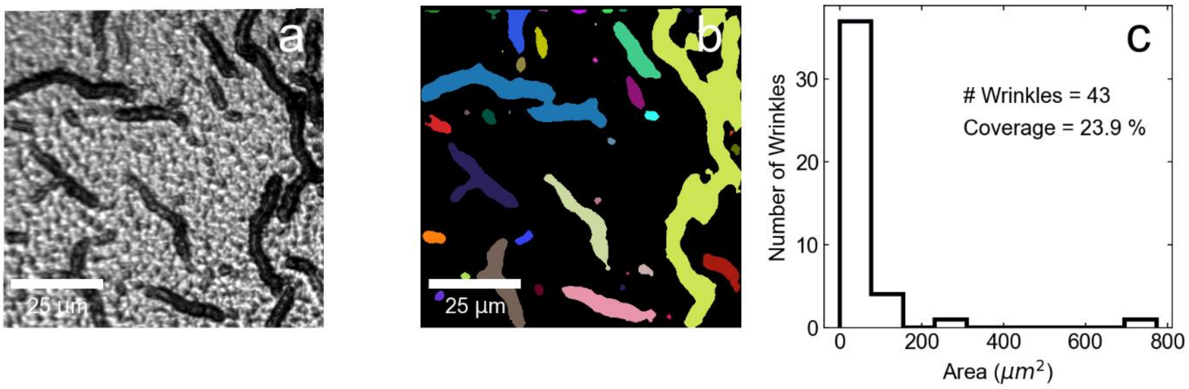

Supplementary Figure 21: a) Bright field reflectance image of 2PACz/DCTH solar cell. b) Filtered wrinkle mask from the reflectance image in a. c) Histogram of the size distribution of wrinkles in panel b.

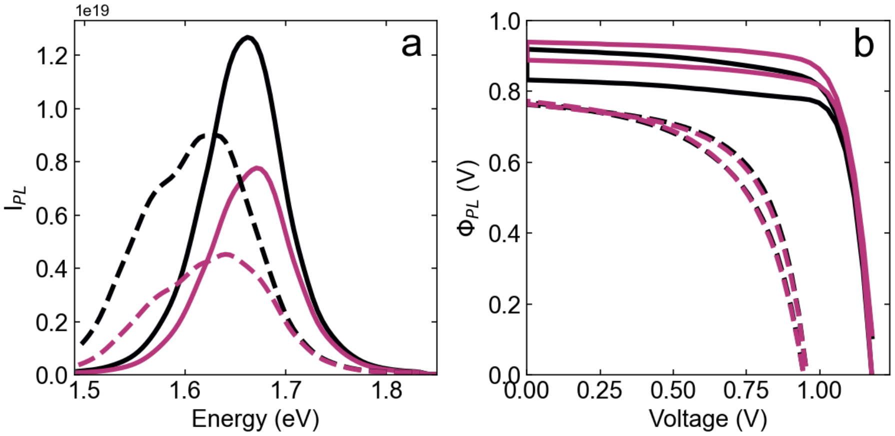

Supplementary Figure 22: a) Average PL spectra of non-wrinkled (red) and wrinkled (black) regions in a 2PACz/DCTH device before (solid lines) and after (dashed lines) operational stress. b) Mean optical JV curves of the same device and masked regions before and after operational stress.

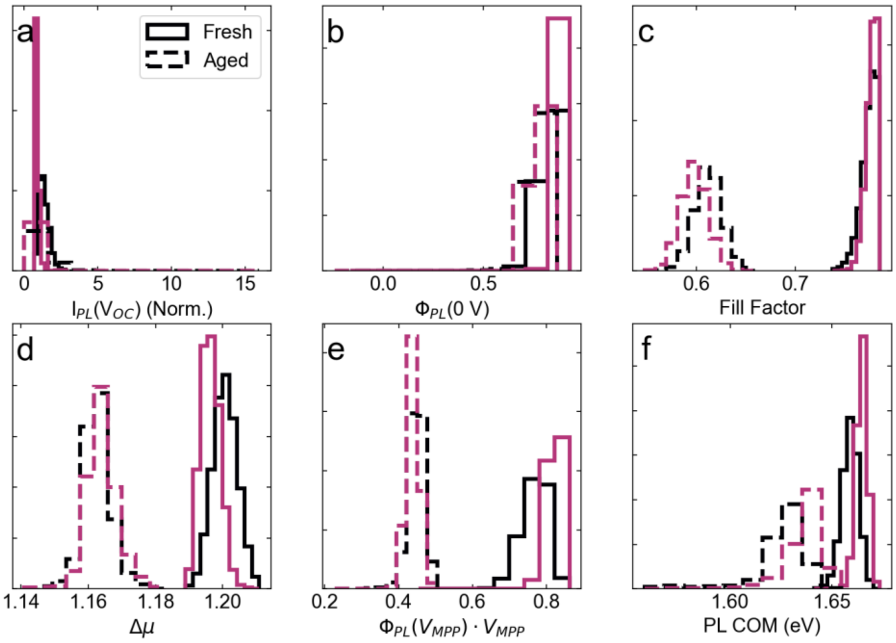

Supplementary Figure 23: Distributions of optoelectronic parameters on non-wrinkled (red) and wrinkled (black) regions before (solid) and after (dashed) operational stress of a 2PACz/DCTH device. a) open circuit PL intensity, b) short circuit optical charge extraction efficiency, c) optical fill factor, d) quasi-Fermi level splitting, e) optical power conversion efficiency and f) PL centre of mass.

We perform the same analysis on the 2PACz/TCTH which we qualitatively observed to have a much lower density of wrinkles than the DCDH and TCTH and plot the distribution in Supplementary Figure 24 where it is evident that both the number of wrinkles per unit area and the total surface coverage of wrinkles in this sample is much lower. This was also observed in equivalent samples with the same TCTH perovskite fabricated on other hole transporting layers (Me-4PACz and MeO-2PACz). Supplementary Figures 25 and 26 show a similar story to the case of the DCTH sample where the wrinkled areas show lower performance in pristine samples but this homogenises after operational stress testing.

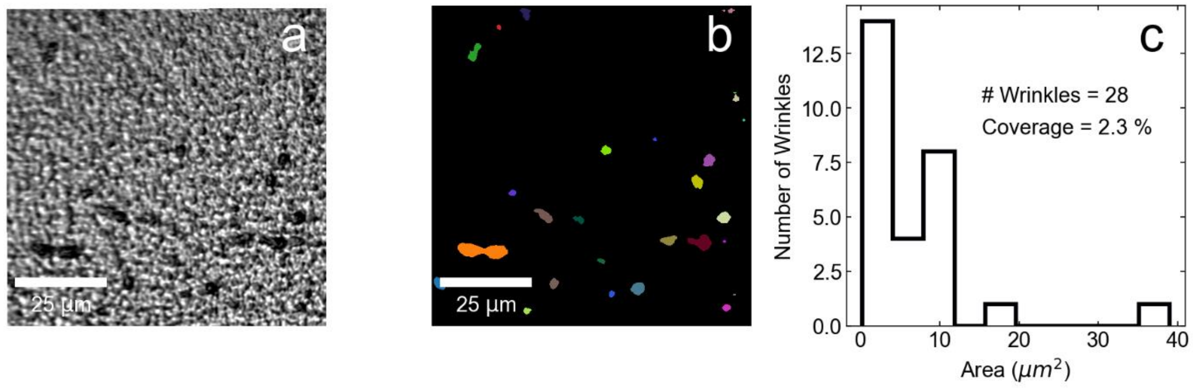

Supplementary Figure 24: a) Bright field reflectance image of 2PACz/TCTH solar cell. b) Filtered wrinkle mask from the reflectance image in a. c) Histogram of the size distribution of wrinkles in panel b.

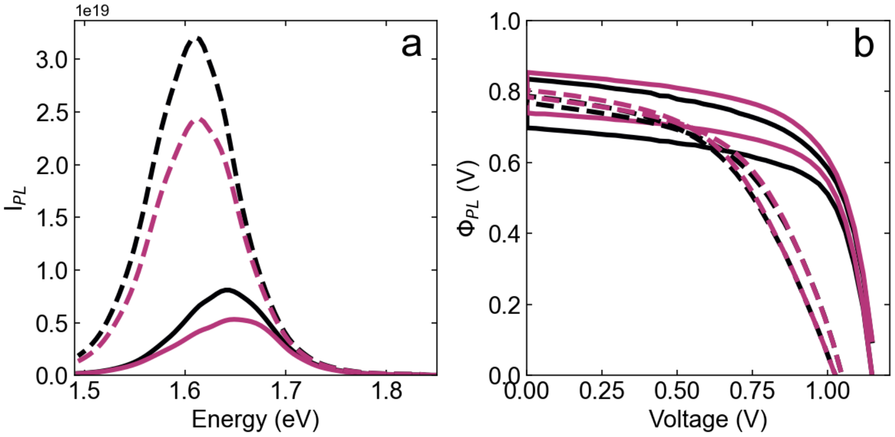

Supplementary Figure 25: a) Average PL spectra of non-wrinkled (red) and wrinkled (black) regions in a 2PACz/TCTH device before (solid lines) and after (dashed lines) operational stress. b) Mean optical JV curves of the same device and masked regions before and after operational stress.

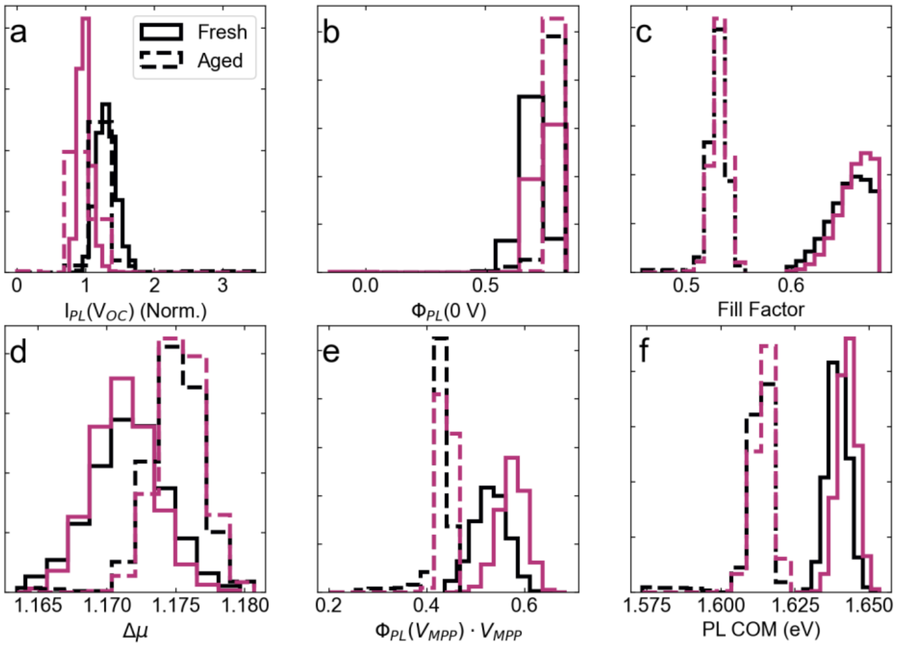

Supplementary Figure 26: Distributions of optoelectronic parameters on non-wrinkled (red) and wrinkled (black) regions before (solid) and after (dashed) operational stress of a 2PACz/TCTH device. a) open circuit PL intensity, b) short circuit optical charge extraction efficiency, c) optical fill factor, d) quasi-Fermi level splitting, e) optical power conversion efficiency and f) PL centre of mass.

One point that is important to mention is that while within batches, the distribution of wrinkling for a given perovskite/device is consistent, this is not always true between batches. Solvent drying, glovebox atmosphere and crystallisation kinetics play a very important role in the formation (or absence) of wrinkling, and the composition of the solution and substrate will certainly play a role, other factors are important to consider including the temperature, atmosphere and solvent vapor pressure in the glovebox at the time of fabrication. However, we find interestingly that even large changes to the wrinkling distribution have little impact on the performance or stability of these devices. To demonstrate this, we show a 2PACz/TCTH device from a separate batch of devices fabricated several months after the above reported samples which has a much higher surface coverage of wrinkles (Supplementary Figure 27), comparable to the distributions in the DCDH and DCTH samples. The optoelectronic and stability behaviour of this device (Supplementary Figure 28 and 29) is almost identical to the much less wrinkled equivalent shown above. The wrinkles have a small negative impact on the charge extraction efficiency in the pristine device which disappears after operation.

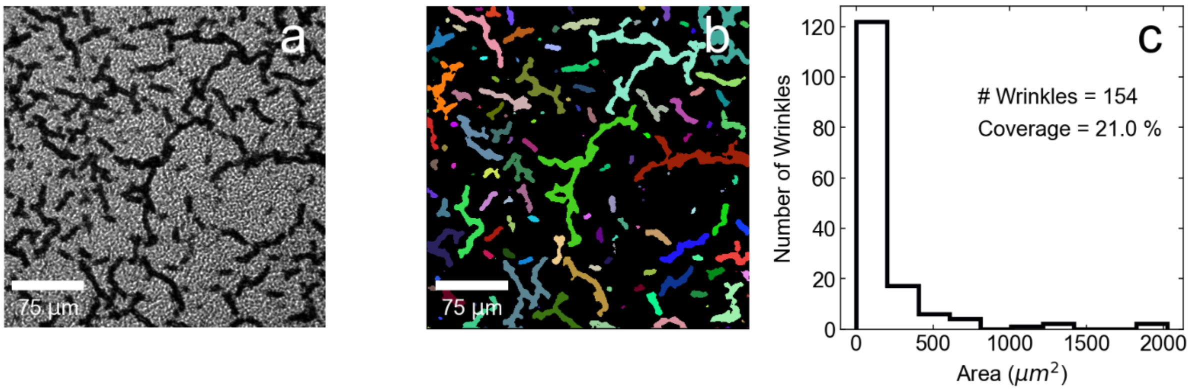

Supplementary Figure 27: a) Bright field reflectance image of 2PACz/TCTH solar cell. b) Filtered wrinkle mask from the reflectance image in a. c) Histogram of the size distribution of wrinkles in panel b.

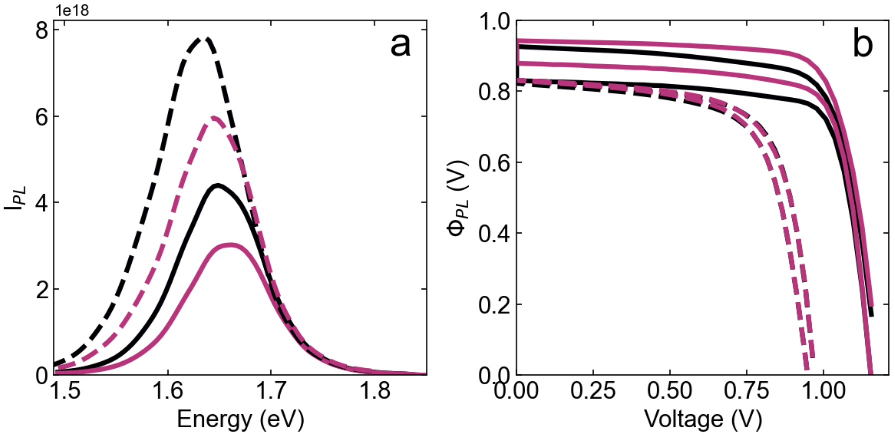

Supplementary Figure 28: a) Average PL spectra of non-wrinkled (red) and wrinkled (black) regions in a 2PACz/TCTH device before (solid lines) and after (dashed lines) operational stress. b) Mean optical JV curves of the same device and masked regions before and after operational stress.

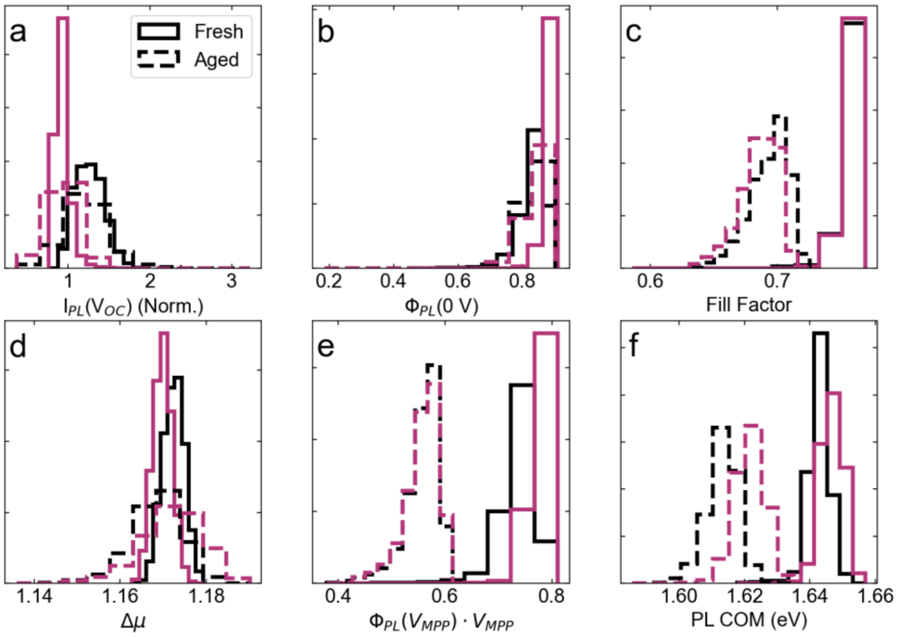

Supplementary Figure 29: Distributions of optoelectronic parameters on non-wrinkled (red) and wrinkled (black) regions before (solid) and after (dashed) operational stress of a 2PACz/TCTH device. a) open circuit PL intensity, b) short circuit optical charge extraction efficiency, c) optical fill factor, d) quasi-Fermi level splitting, e) optical power conversion efficiency and f) PL centre of mass.

To confirm that our optical measurements were not somehow missing a proportion of the wrinkling or light outcoupling was causing distortions skewing our view of their size and distribution, we used another morphology sensitive imaging technique, scanning electron microscopy (SEM) in order to image the wrinkles and grain structure across all of the device stacks studied. The images of the large scales wrinkle structures and smaller scale apparent grain morphologies are shown in Supplementary Figure 30. The relatively sparse coverage of the wrinkles and their interesting shape and features across each individual wrinkle matches well with our optical measurements, giving us further confidence that our above analysis is valid. To show that these features are not just 2-dimensional texture in the film and to get a closer look at the structure of the wrinkles, we performed some tilted SEM images with the devices tilted at 40° relative to normal incidence which are shown in Supplementary Figure 25. Several observations are immediately apparent from these images. The first simple observation is that the wrinkles do indeed have an appreciable height change relative to their surroundings as expected. The second is that rather than each wrinkles having a single ‘peak’, each wrinkle has two peaks, one on each side surrounded by a valley in the centre. This structure helps us explain some observations observed in the optical microscopy data where often each wrinkle

would appear as one entity in bright-field reflection mode, but as two distinct features in PL (see Figure 1h, Supplementary Figure 21 for examples). This unusual morphology clearly has a defining role in this effect. The final, more unexpected observation is that the morphological grains on the wrinkles are substantially larger on average than the surrounding flat film. The red shift of the PL from these domains may therefore also be influenced by the larger grain size, in addition to the thickness change and compositional variation.

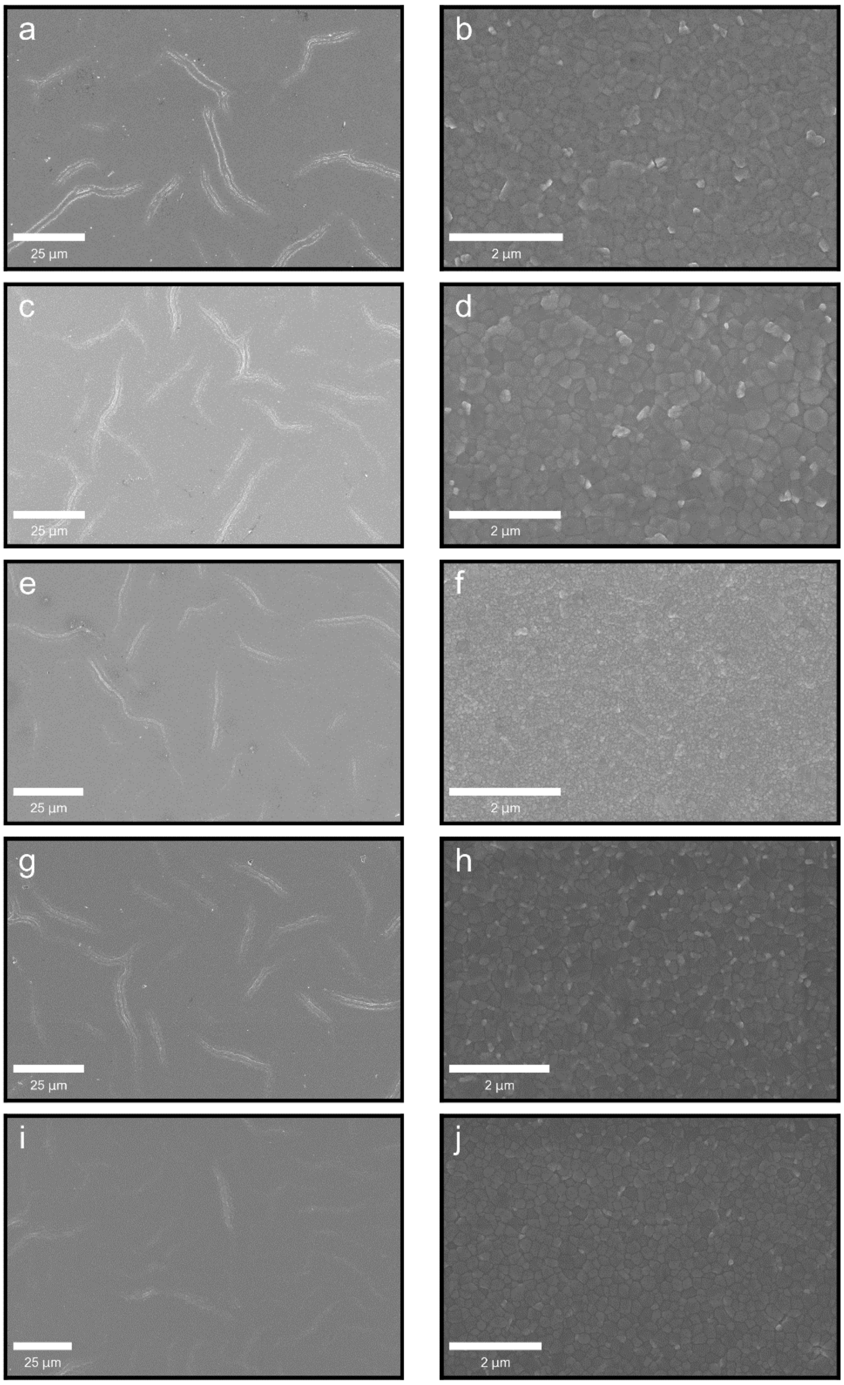

Supplementary Figure 30: Low mag images showing the larger scale wrinkle structure and small-scale grain structure respectively of a), b) Me-4PACz/TCTH, c), d), MeO-2PACz/TCTH, e), f), 2PACz/TCTH, g),h), 2PACz/DCTH and i), j) 2PACz/DCDH.

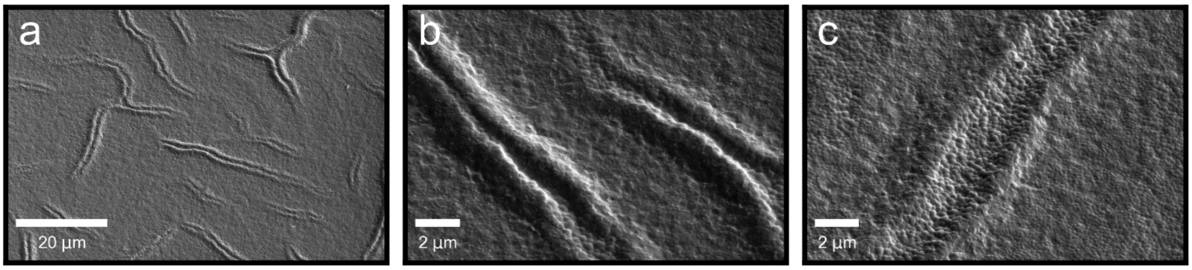

Supplementary Figure 31: SEM image of a) and b) 2PACz/DCTH and c) 2PACz/DCDH wrinkled perovskite devices taken at 40° tilt angle.

Taken together, these results suggest that the wrinkles are quite benign to device stability as they do not appear to be the seeds of spreading degradation or degrade any more than their surroundings. This is somewhat surprising as we might naively assume that these large-scale heterogeneities may invite degradation, but this appears not to be the case.

Supplementary Note 7: Hysteresis Spatial Variations

Here we investigate whether the local emission properties of the perovskite (Δμ, open circuit PL intensity and COM) correlate more strongly with optoelectronic properties measured from voltage dependent PL mapping measured in either the forward or reverse voltage scan direction. To investigate this further, we took 2PACz/DCDH dataset and first performed autocorrelation plots between figures of merit extracted from the reverse and forward scans. If these are highly correlated, it means that the optical figures of merit (Δμ and COM) will have a similar correlation to both scan directions. In Supplementary Figure 32, we plot the autocorrelation of the optical extraction efficiency, fill factor and PCE in the pristine 2PACz/DCDH device. Each column in the figure shows maps of the reverse and forward scan figures of merit, the histograms of their respective distributions before a 2D histogram to show the autocorrelations. Both qualitative inspection and the statistical analysis from the Spearman's coefficient indicate a strong spatial correlation between the forward and reverse direction figures of merit in the pristine sample, implying the optical figures of merit will be correlated equally with the figures of merit from both scan directions. We confirm this in Supplementary Figure 33 where once again, both the qualitative appearance and statistical analysis of the data of both Δμ and COM correlations are very similar in both forward and reverse scans.

The situation is more complex after the operational stress test as there is a particular disparity between forward and reverse scans in this device. We plot the autocorrelation of the stress tested figures of merit in Supplementary Figure 34. In this device, the optical extraction efficiency is still relatively homogeneous in the forward and reverse directions, however, strikingly we find a strong gradient in performance across the sample from one corner to the other which particularly manifests in the fill factor and the resulting optical PCE. However, the gradients are of opposite signs in the forward and reverse scans, strongly suggesting that the gradient observed is due to a gradient in mobile ionic concentrations. This means that there is a positive autocorrelation in the extraction efficiency but a negative correlation in both FF and optical PCE. The gradient in the fill factor in the forward scan qualitatively matches the gradient of the optical figures of merit, in particular Δμ. The correlations are shown in Supplementary Figure 35. The COM and Δμ values positively correlate with forward direction figures of merit and anticorrelate with the reverse scan figures, suggesting that the 'true' optoelectronic performance of the perovskite itself is exhibited in the forward scan, before being somewhat masked by the ionic effects. However, we caveat this statement that given

these values are particularly affected by the large lateral variation caused by the edge effects, it is worth considering a region that is less affected by these effects. We perform the same correlation analysis on a different region of the same stress tested device that is less hysteretic (Supplementary Figure 36). Here we find that the optical figures of merit correlate positively with the reverse scan, while there is a more complex, non-monotonic relationship with the forward scan. We assert that in a complete device, simply measuring the microscopic properties of the perovskite ($\Delta\mu$ and COM) in isolation is not sufficient to determine local performance, particularly in cases with large concentrations of mobile ionic species and hysteresis.

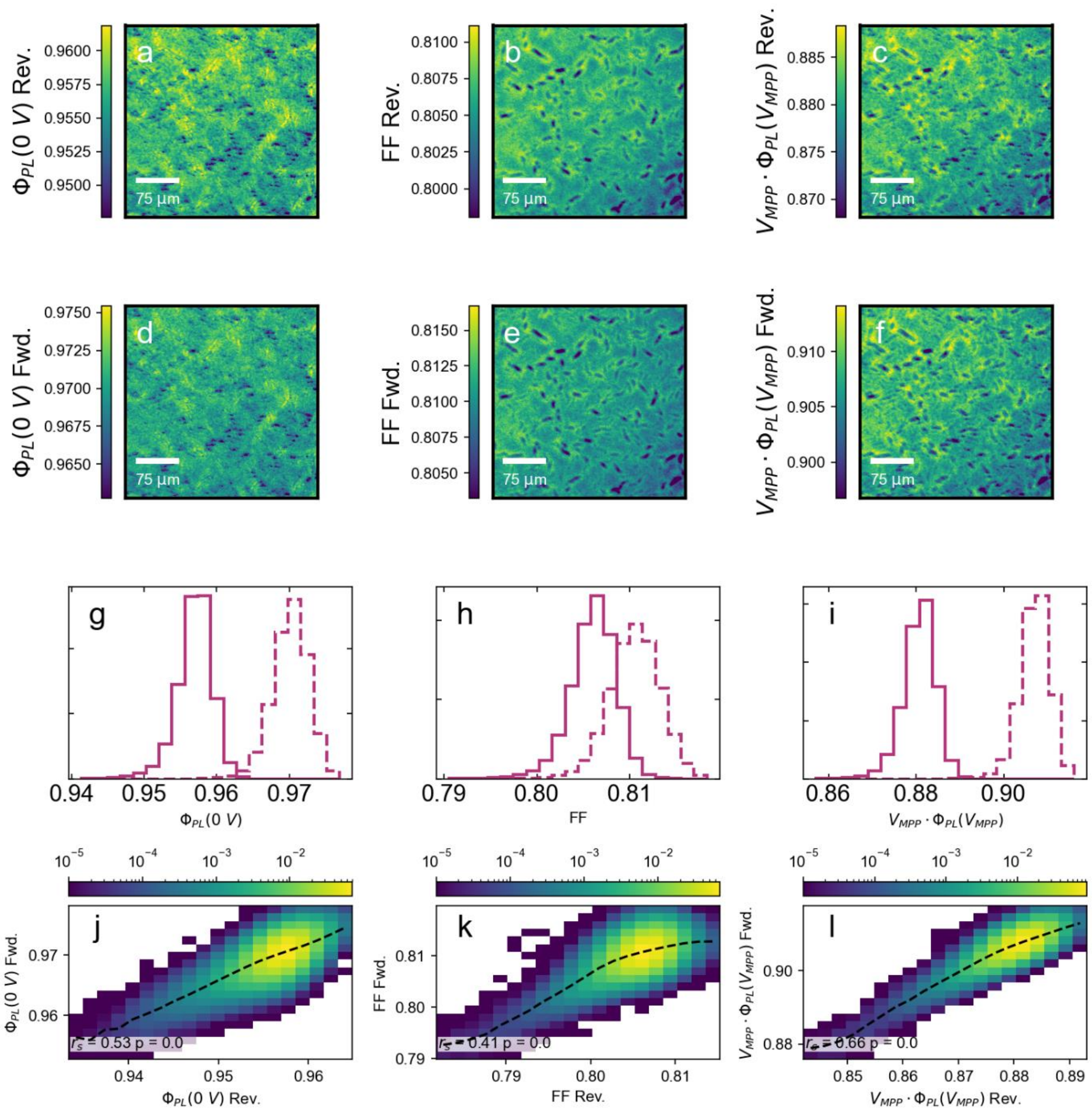

Supplementary Figure 32: Optical JV parameters extracted in both the reverse and forward directions from a pristine 2PACz/DCDH solar cell. Maps of the reverse scan a) optical extraction efficiency, b) fill factor and c) optical PCE. Maps of the forward scan d) optical extraction efficiency, e) fill factor and f) optical PCE. Histograms comparing the distributions of g) optical extraction efficiency, h) FF and i) optical PCE extracted in reverse (solid line) and forward (dashed line) figures of merit. 2D histograms showing the correlation with j) optical extraction efficiency, k) fill factor and i) optical PCE in the reverse and forward direction. The black dashed curves represent the mean value of the component on the y axis in each window of the variable on the x axis and are guides to the eye. Overlaid over each map is the Spearman's rank correlation coefficient between the two variables and the related p-value (two-sided test).

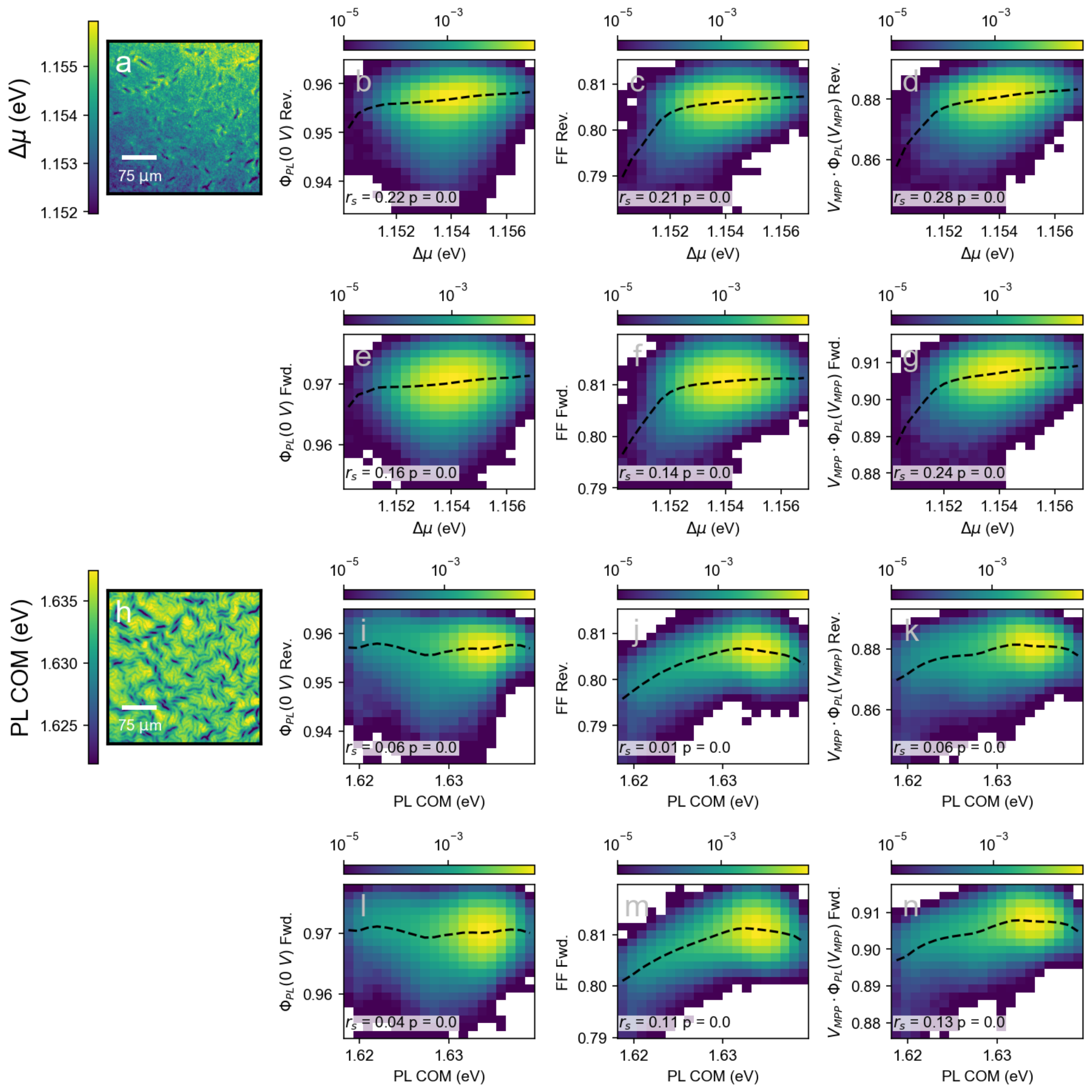

Supplementary Figure 33: Correlations between optical figures of merit and optoelectronic figures of merit extracted from forward and reverse scans in pristine 2PACz/DCDH solar cell. a) Map of Δμ. 2D histograms showing correlation between Δμ and b), e) optical extraction efficiency, c) f) fill factor and d), g) optical PCE in the reverse and forward directions respectively. h) Map of COM. 2D histograms showing correlation between COM and i), l) optical extraction efficiency, j) m) fill factor and k), n) optical PCE in the reverse and forward directions respectively. The black dashed curves represent the mean value of the component on the y axis in each window of the variable on the x axis and are guides to the eye. Overlaid over each map is the Spearman’s rank correlation coefficient between the two variables and the related p-value (two-sided test).

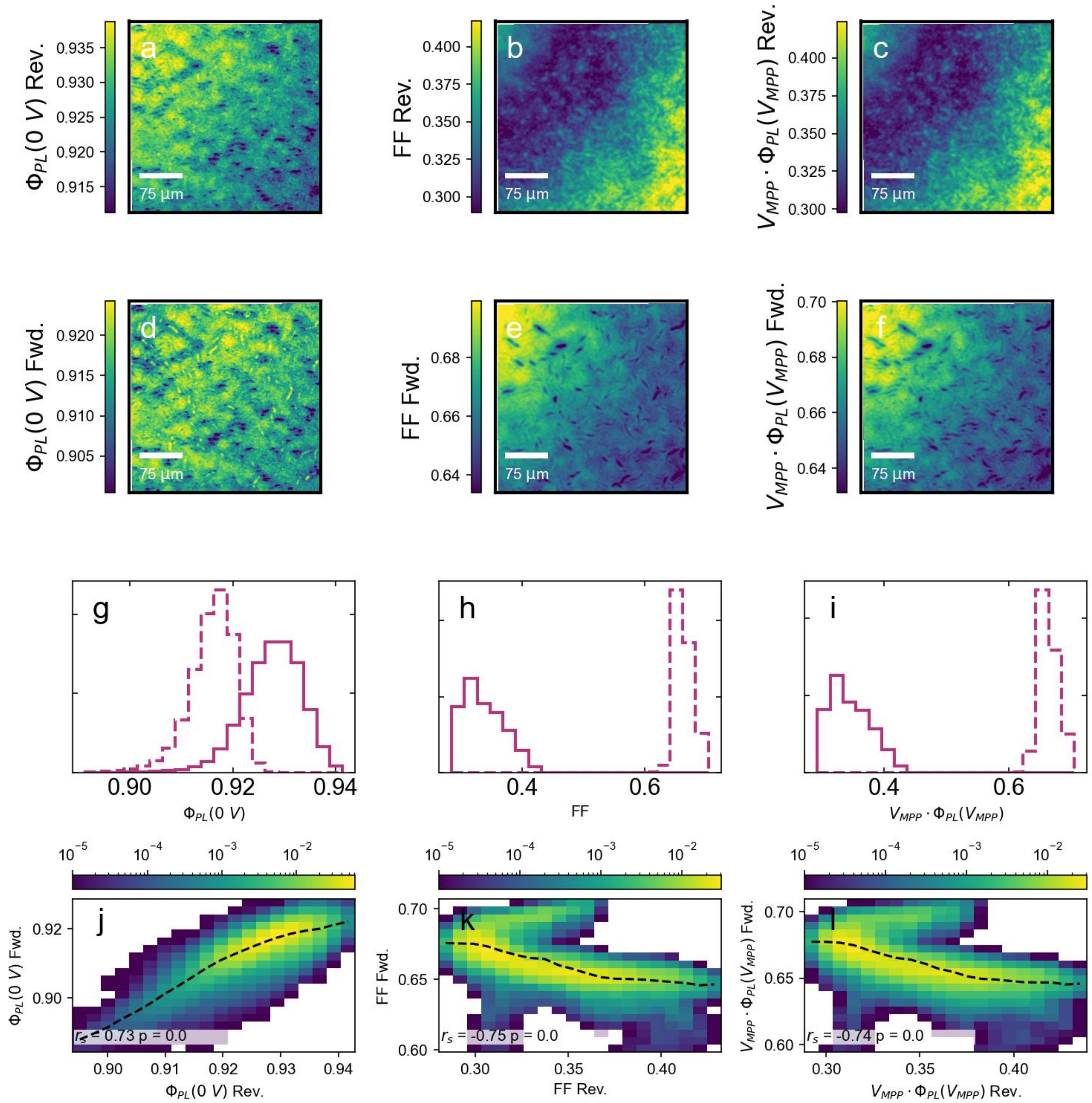

Supplementary Figure 34: Optical JV parameters extracted in both the reverse and forward directions from an operationally stress tested 2PACz/DCDH solar cell. Maps of the reverse scan a) optical extraction efficiency, b) fill factor and c) optical PCE. Maps of the forward scan d) optical extraction efficiency, e) fill factor and f) optical PCE. Histograms comparing the distributions of g) optical extraction efficiency, h) FF and i) optical PCE extracted in reverse (solid line) and forward (dashed line) figures of merit. 2D histograms showing the correlation with j) optical extraction efficiency, k) fill factor and i) optical PCE in the reverse and forward direction. The black dashed curves represent the mean value of the component on the y axis in each window of the variable on the x axis and are guides to the eye. Overlaid over each map is the Spearman's rank correlation coefficient between the two variables and the related p-value (two-sided test).

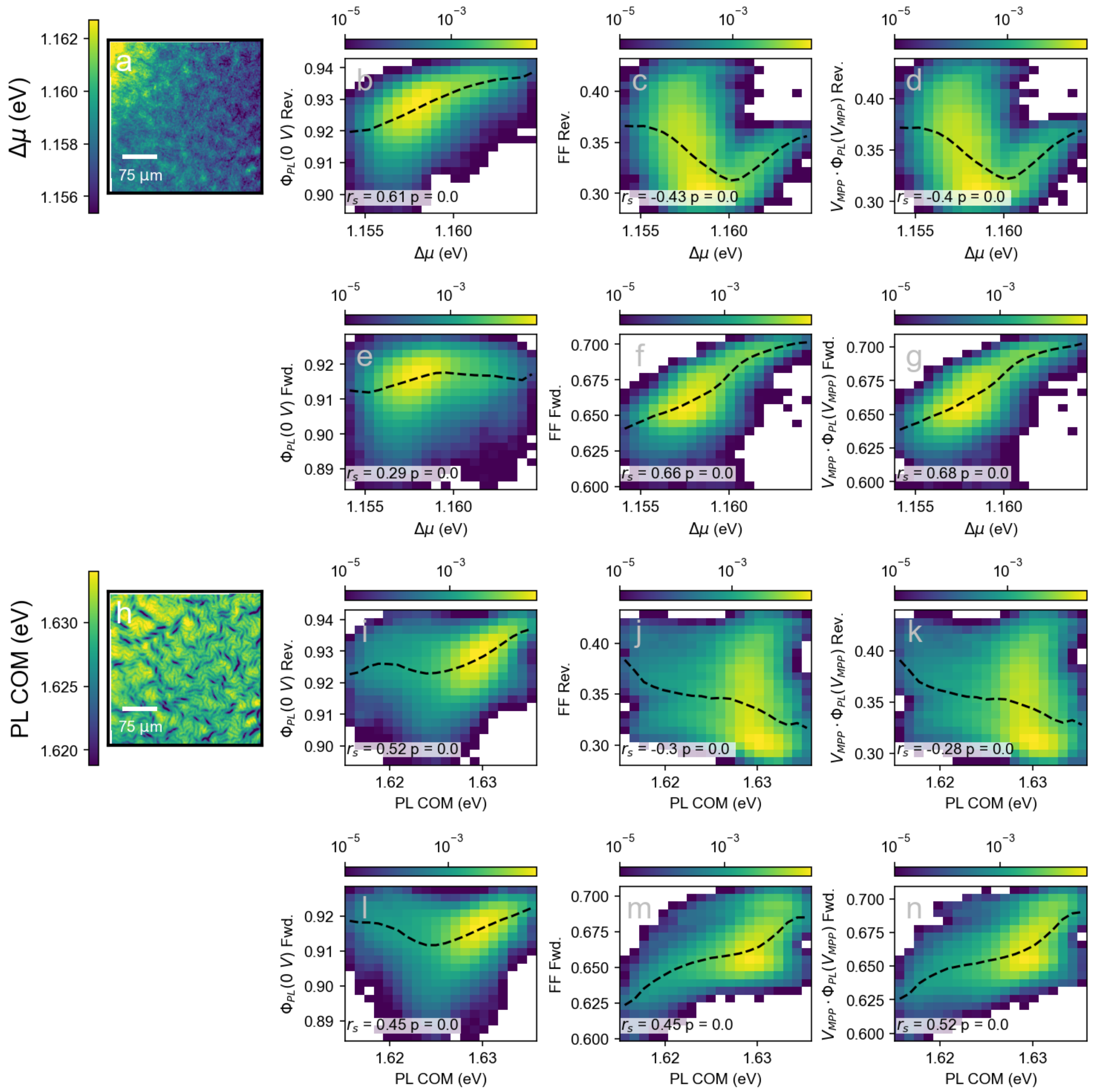

Supplementary Figure 35: Correlations between optical figures of merit and optoelectronic figures of merit extracted from forward and reverse scans in operationally stress tested 2PACz/DCDH solar cell. a) Map of Δμ. 2D histograms showing correlation between Δμ and b), e) optical extraction efficiency, c) f) fill factor and d), g) optical PCE in the reverse and forward directions respectively. h) Map of COM. 2D histograms showing correlation between COM and i), l) optical extraction efficiency, j) m) fill factor and k), n) optical PCE in the reverse and forward directions respectively. The black dashed curves represent the mean value of the component on the y axis in each window of the variable on the x axis and are guides to the eye. Overlaid over each map is the Spearman's rank correlation coefficient between the two variables and the related p-value (two-sided test).

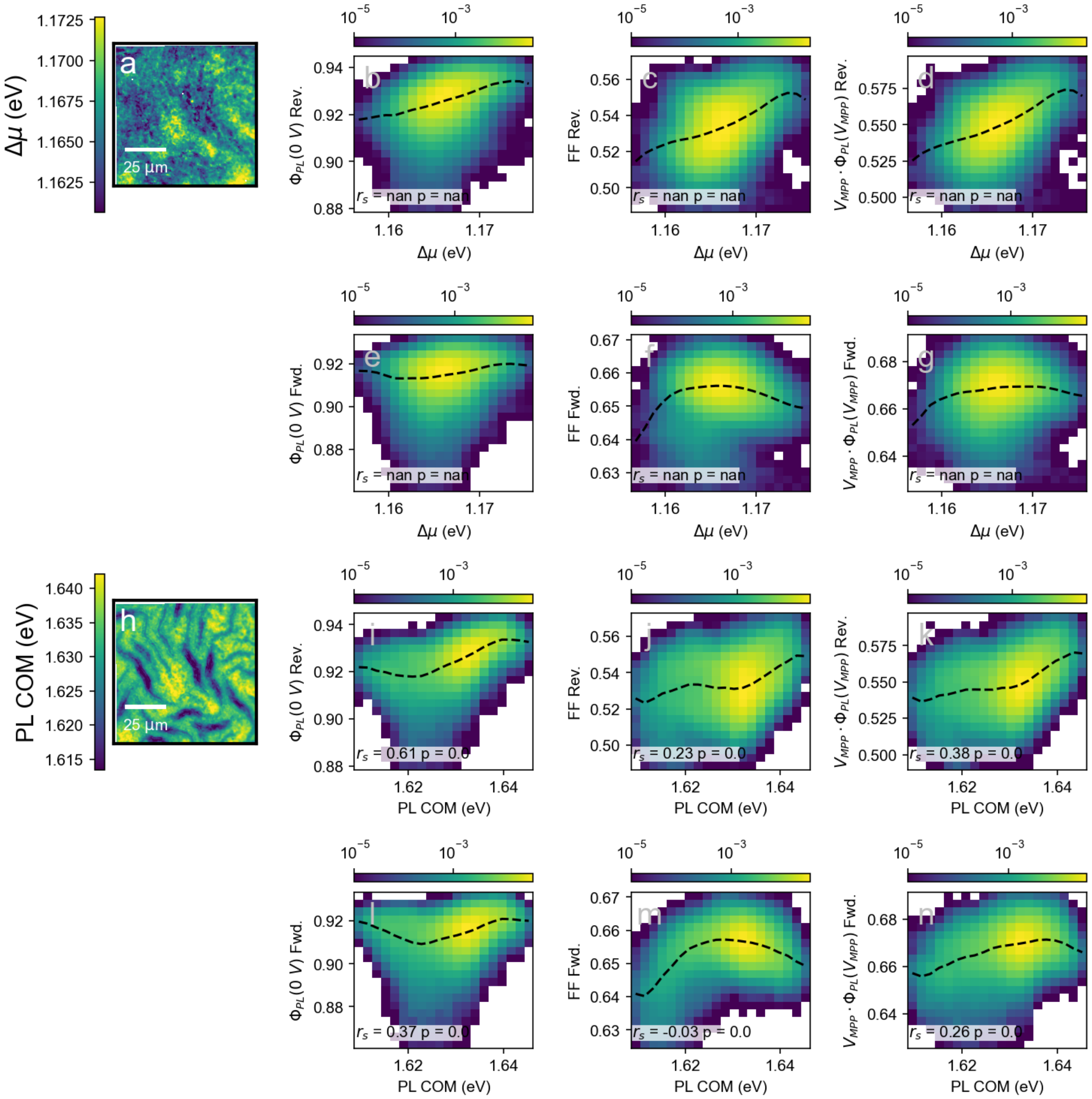

Supplementary Figure 36: Correlations between optical figures of merit and optoelectronic figures of merit extracted from forward and reverse scans in operationally stress tested 2PACz/DCDH solar cell. a) Map of Δμ. 2D histograms showing correlation between Δμ and b), e) optical extraction efficiency, c) f) fill factor and d), g) optical PCE in the reverse and forward directions respectively. h) Map of COM. 2D histograms showing correlation between COM and i), l) optical extraction efficiency, j) m) fill factor and k), n) optical PCE in the reverse and forward directions respectively. The black dashed curves represent the mean value of the component on the y axis in each window of the variable on the x axis and are guides to the eye. Overlaid over each map is the Spearman's rank correlation coefficient between the two variables and the related p-value (two-sided test).

Supplementary Figures

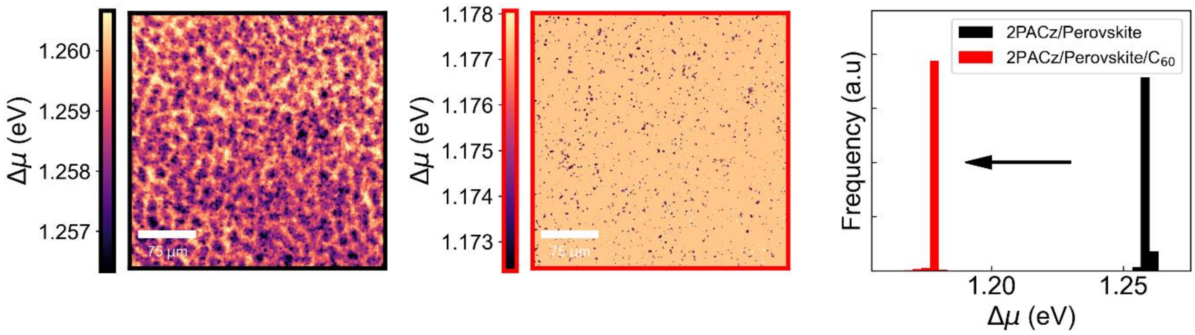

Supplementary Figure 37: Δμ maps of perovskite solar cell half stacks. a) Δμ map of a half stack consisting of ITO/2PACz/TCTH perovskite and b) the same stack with C_{60} evaporated on top.

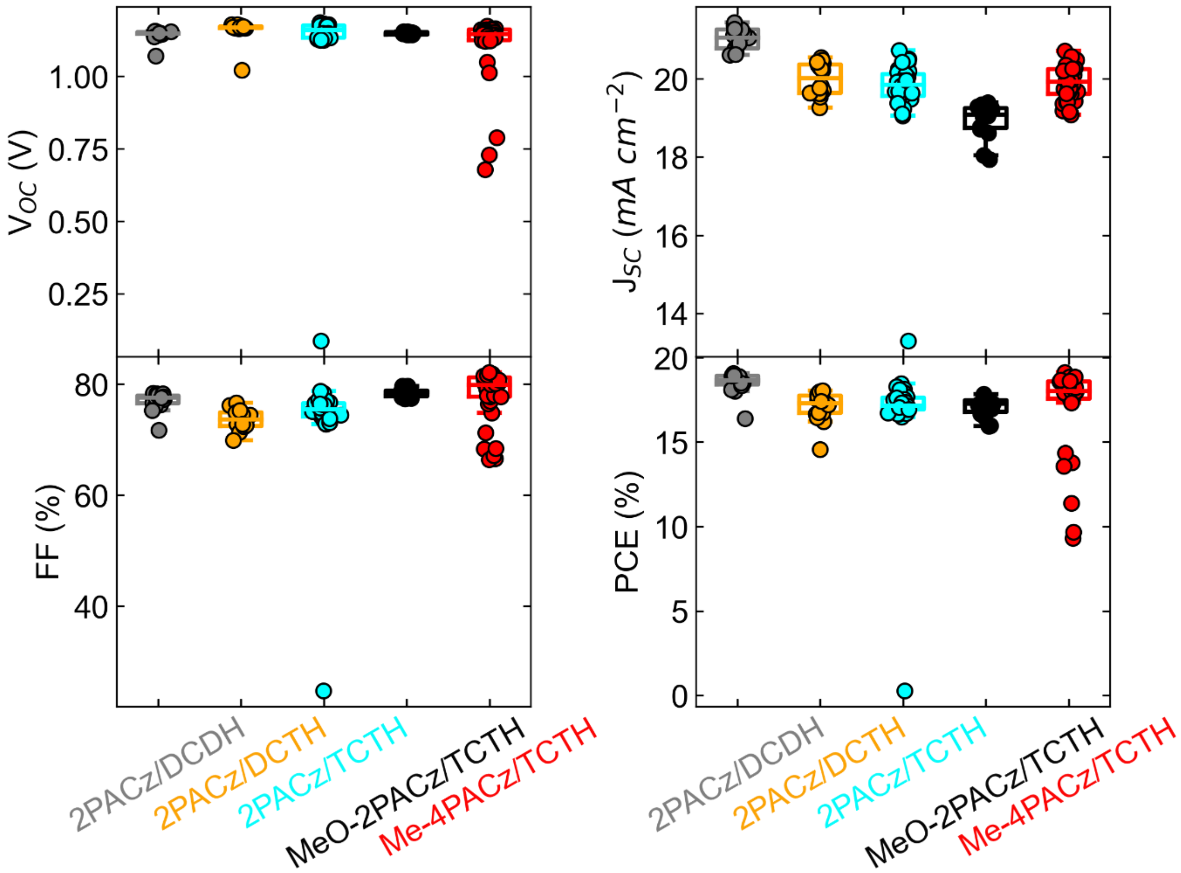

Supplementary Figure 38: Electrically measured JV figures of merit. Box plots of the 4 main solar cell figures of merit across the compositional and HTL phase space described in the main text. Data shown for 116 devices. Boxplot horizontal lines represent the median, the box represents the 1st and 3rd quartile. Whiskers extend to the last data point less/greater than the upper/lower quartile plus 1.5 times the interquartile range.

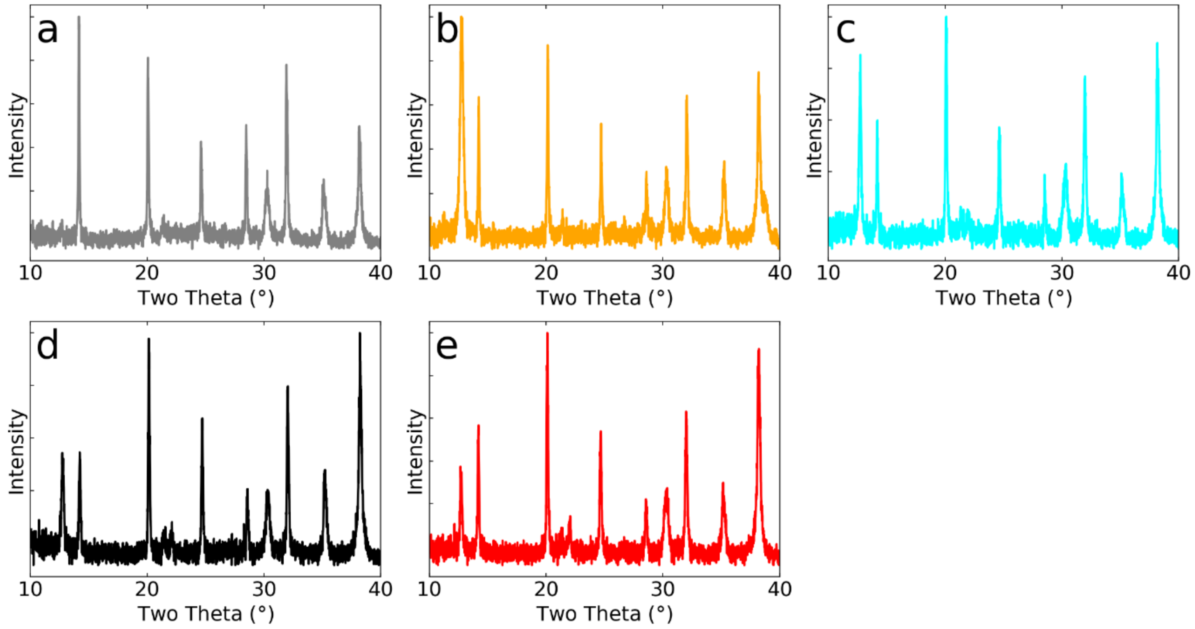

Supplementary Figure 39: XRD patterns of the 5 main device stacks in the study. a) 2PACz/DCDH, b) 2PACz/DCTH, c) 2PACz/TCTH, d) MeO-2PACz/TCTH, e) Me-4PACz/TCTH.

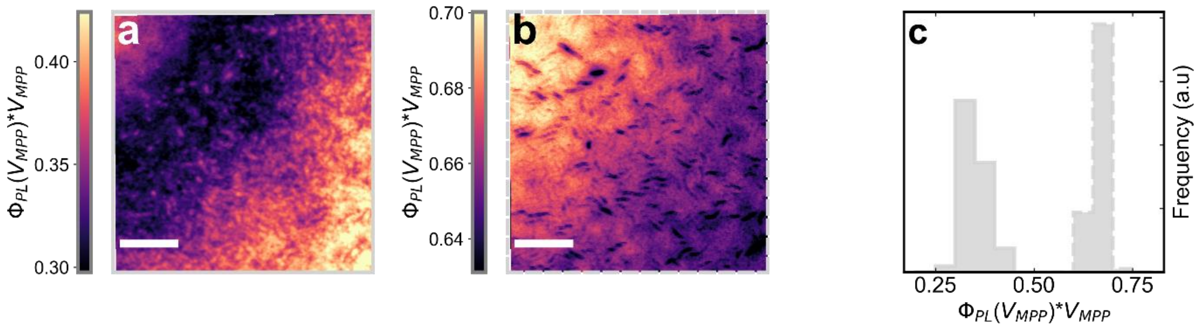

Supplementary Figure 40: Spatial distribution of hysteresis in DCDH solar cell after operational stress test. Optical PCE maps of DCDH solar cells after operational stress extracted from the a) reverse scan (as shown in Figure 2) and b) the forward voltage sweep. Scalebars are 75 µm. c) Histogram of optical PCE values from the maps in a and b (dashed lines represent forward voltage sweep values).

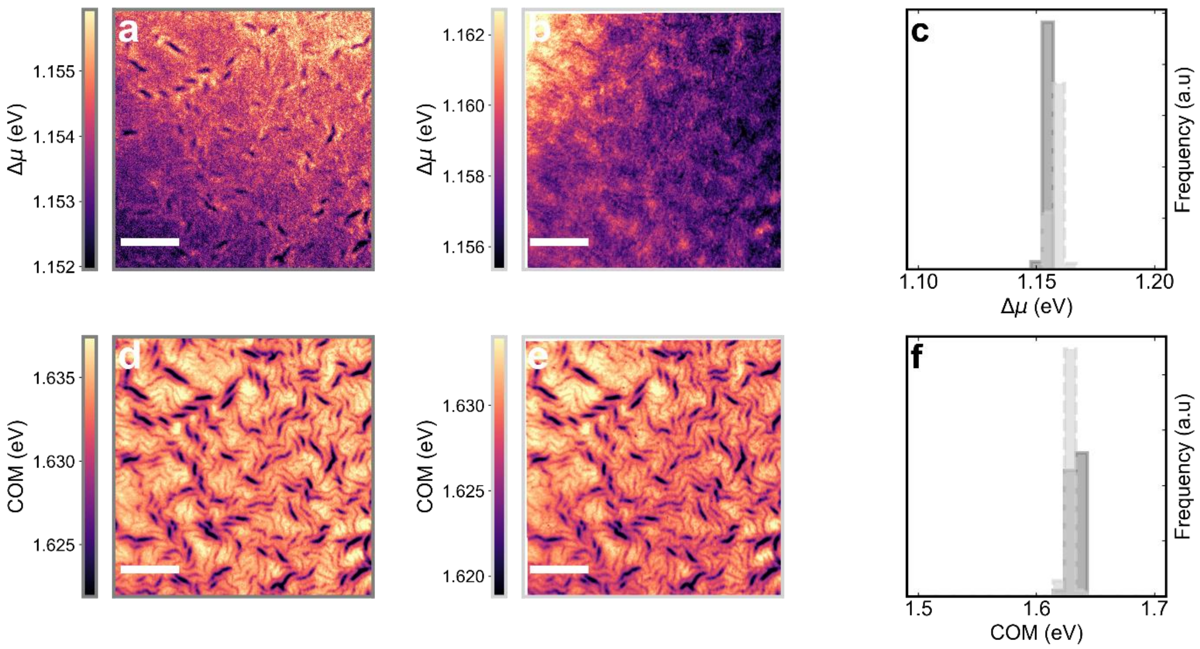

Supplementary Figure 41: Δμ and COM of DCDH solar cell before after accelerated ageing. Δμ a) before and b) after accelerated ageing, distributions summarised in panel c). Solid bars are before ageing, dashed bars are after ageing. COM d) before and e) after accelerated ageing, distributions summarised in panel f). Dark gray is before and light gray is after the accelerated ageing protocol. The scan area is the same as for the voltage dependent measurement shown in Figure 2. Scalebars are 75 μm.

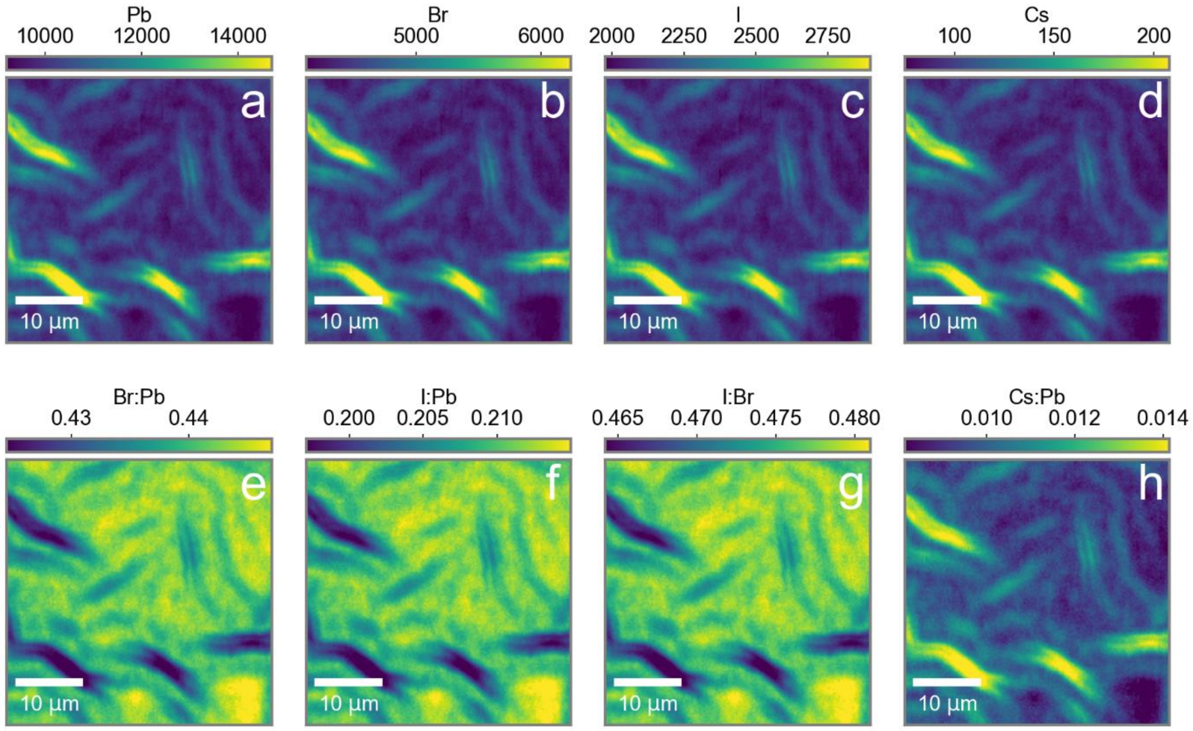

Supplementary Figure 42: nXRF maps of 2PACz/DCDH solar cell. XRF maps of region 1 from Figure 2 in the main text showing the a) Pb L, b) Br K, c) I L and d) Cs L lines. Ratios of the e) Br:Pb lines, f) I:Pb lines, g) I:Br lines and h) Cs:Pb lines. Scalebars are 10 µm.

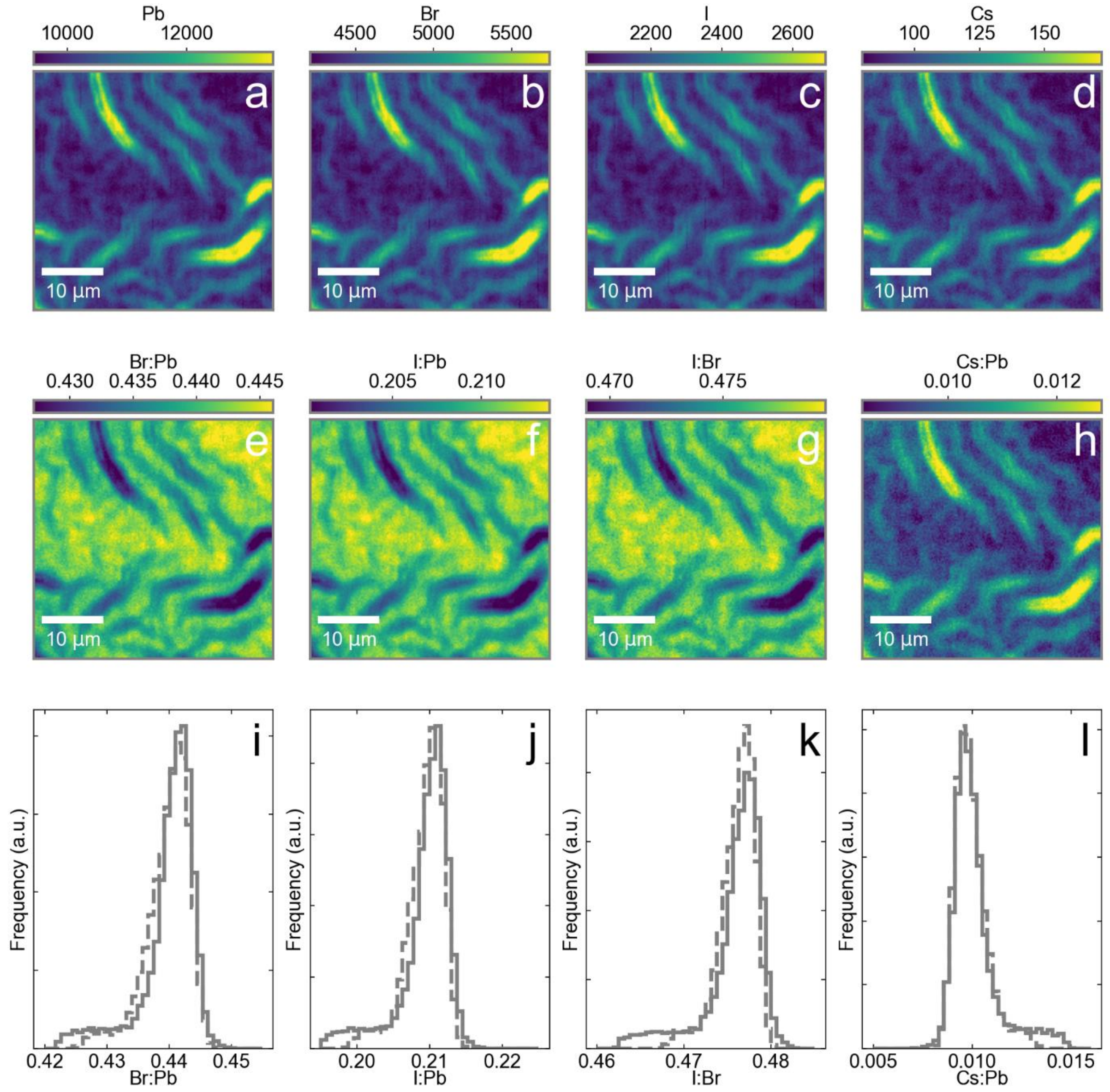

Supplementary Figure 43: nXRF maps of region 2 of 2PACz/DCDH solar cell. XRF maps of region 2 from Figure 2 in the main text showing the a) Pb L, b) Br K, c) I L and d) Cs L lines. Ratios of the e) Br:Pb lines, f) I:Pb lines, g) I:Br lines and h) Cs:Pb lines. Histograms comparing the XRF intensities between region 1 (solid line) and region 2 (dashed lines) for the i) Br:Pb ratio, j) I:Pb ratio, k) I:Br and l) Cs:Pb lines.

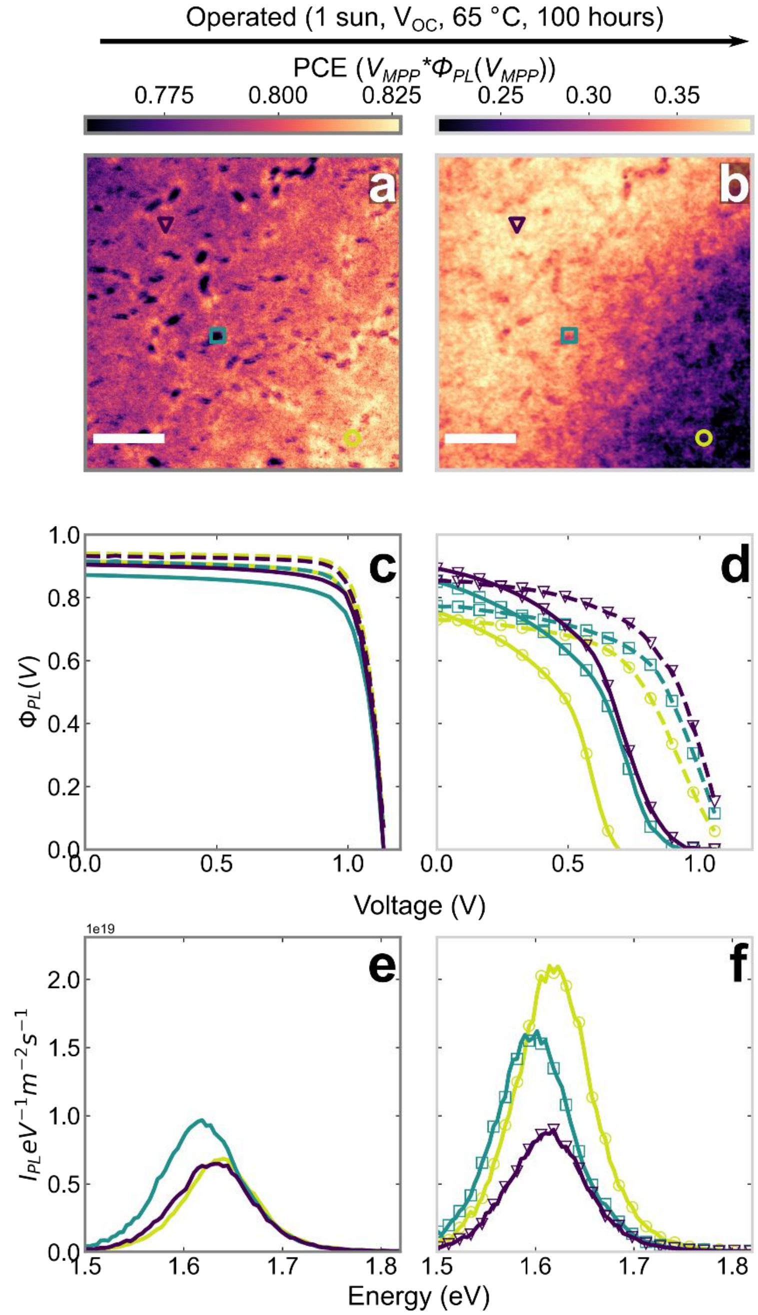

Supplementary Figure 44: Optical and electronic spectra of 2PACz/DCDH solar cell before and after operational stress test. Optical PCE maps of the same area of a fresh a) and operated b) DCDH solar cell after 100 hours at V_{OC}, 65 °C and 1 sun illumination. c) and d) Optical JV curves before and after ageing from the points marked in panels a and b. Solid lines are reverse scans, dashed lines are forward scans. e) and f) PL spectra from the same marked areas before and after ageing respectively. Scalebars in a and b are 75 µm.

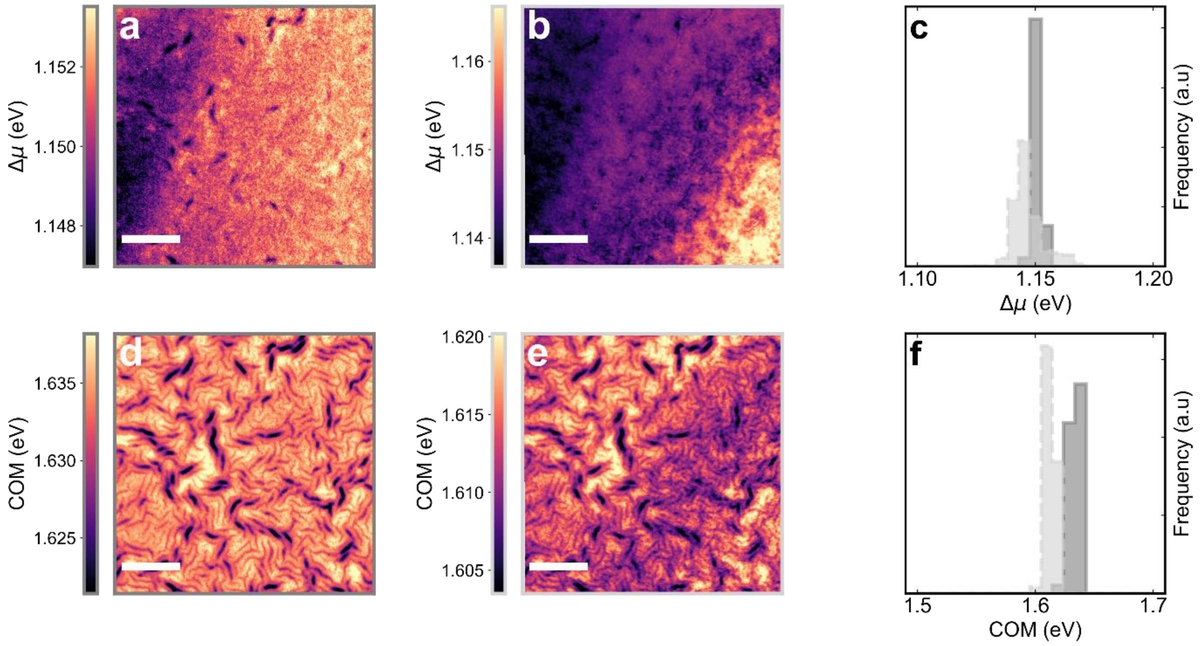

Supplementary Figure 45: Δμ and COM of 2PACz/DCDH solar cell before after opertaional stress test. Δμ a) before and b) after accelerated ageing, distributions summarised in panel c). Solid bars are before ageing, dashed bars are after ageing. COM d) before and e) after accelerated ageing, distributions summarised in panel f). Dark gray is before and light gray is after the operational stress protocol. The scan area is the same as for the voltage dependent measurement shown in Supplementary Figure 13. Scalebars are 75 μm.

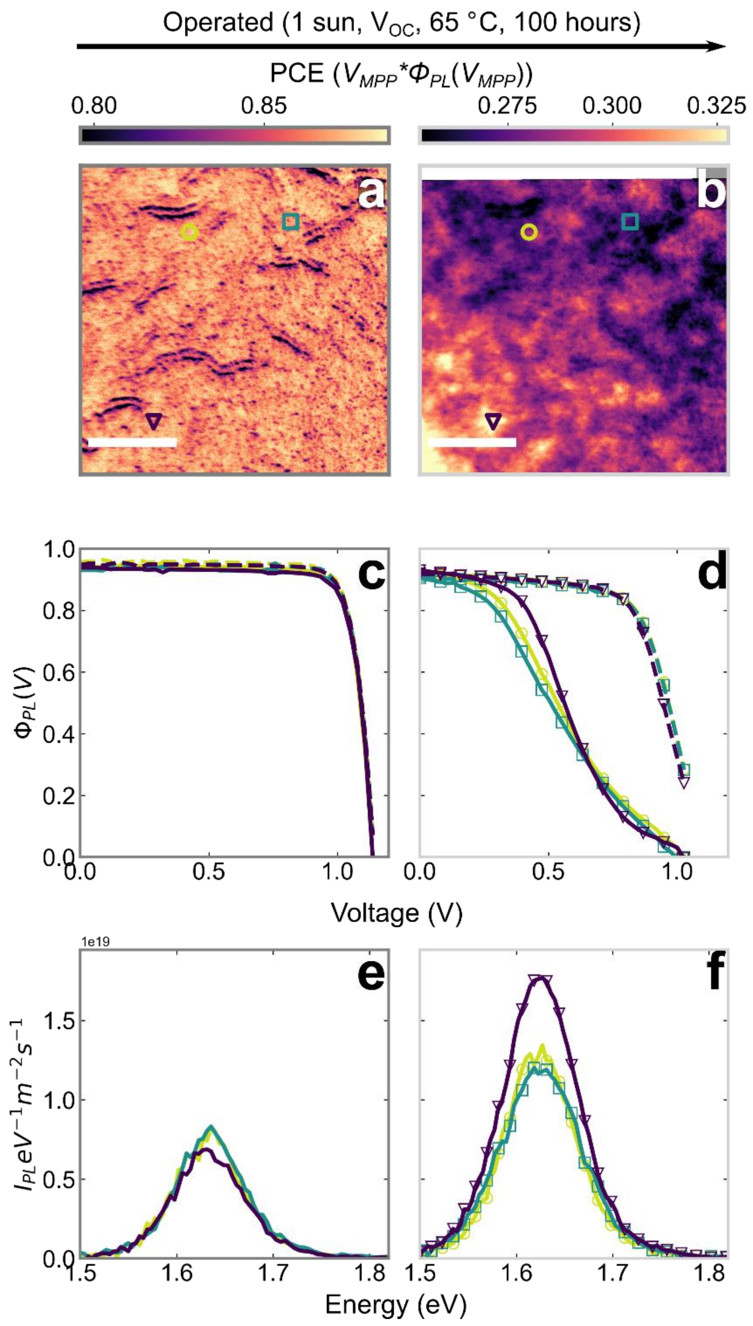

Supplementary Figure 46: Optical and electronic spectra of a 2PACz/DCDH solar cell before and after operational stress test away from pixel edge. Optical PCE maps of the same area of a fresh a) and operated b) DCDH solar cell after 100 hours at V_{OC}, 65 °C and 1 sun illumination. c) and d) Optical JV curves before and after ageing from the points marked in panels a and b. Solid lines are reverse scans, dashed lines are forward scans. e) and f) PL spectra from the same marked areas before and after ageing respectively. Scalebars in a and b are 25 μm.

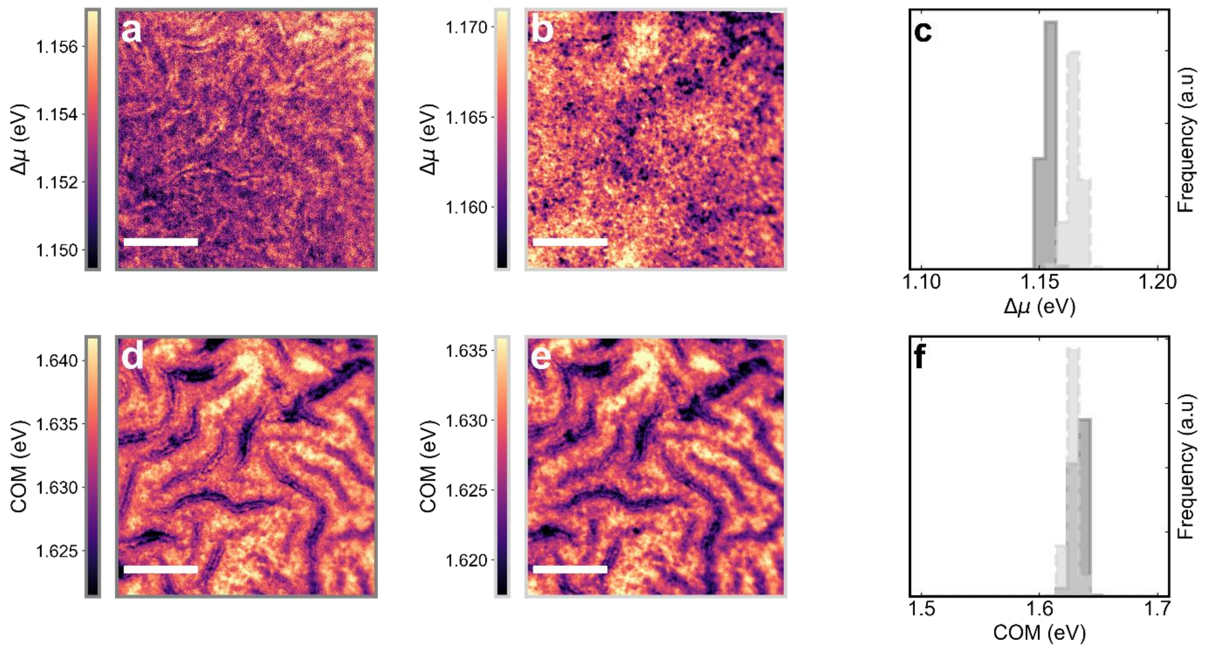

Supplementary Figure 47: Δμ and COM of DCDH solar cell before after accelerated ageing. Δμ a) before and b) after accelerated ageing, distributions summarised in panel c). Solid bars are before ageing, dashed bars are after ageing. COM d) before and e) after accelerated ageing, distributions summarised in panel f). Dark gray is before and light gray is after the accelerated ageing protocol. The scan area is the same as for the voltage dependent measurement shown in Supplementary Figure 39. Scalebars are 25 μm.

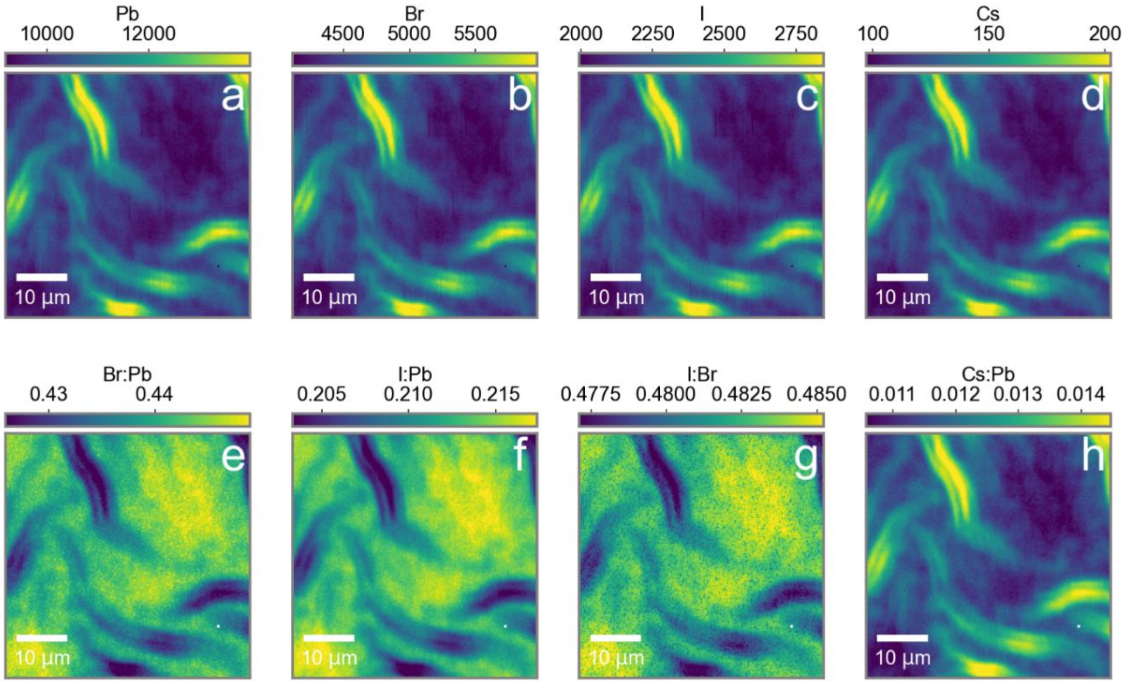

Supplementary Figure 48: nXRF maps of a 2PACz/DCDH solar cell. nXRF maps of the a) Pb L, b) Br K, c) I L and d) Cs L lines. Ratios of the e) Br:Pb lines, f) I:Pb lines, g) I:Br lines and h) Cs:Pb lines. Scalebars are 10 µm.

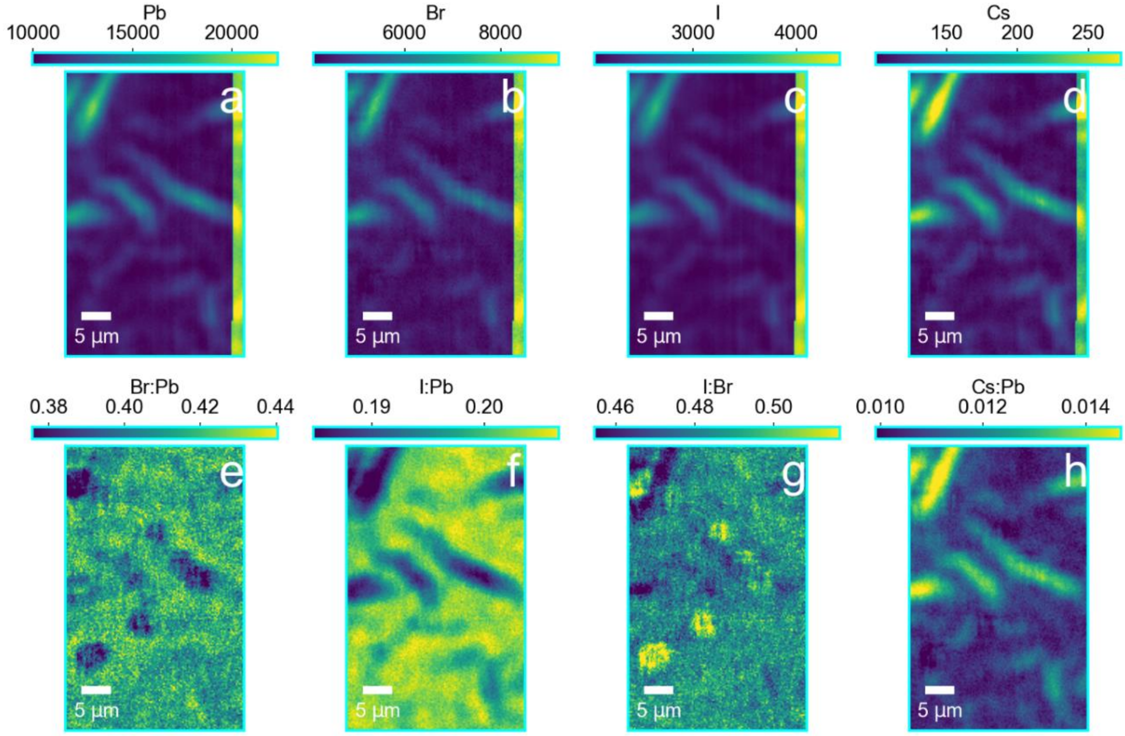

Supplementary Figure 49: nXRF maps of a 2PACz/DCTH solar cell. nXRF maps of the a) Pb L, b) Br K, c) I L and d) Cs L lines. Ratios of the e) Br:Pb lines, f) I:Pb lines, g) I:Br lines and h) Cs:Pb lines. Scalebars are 5 µm.

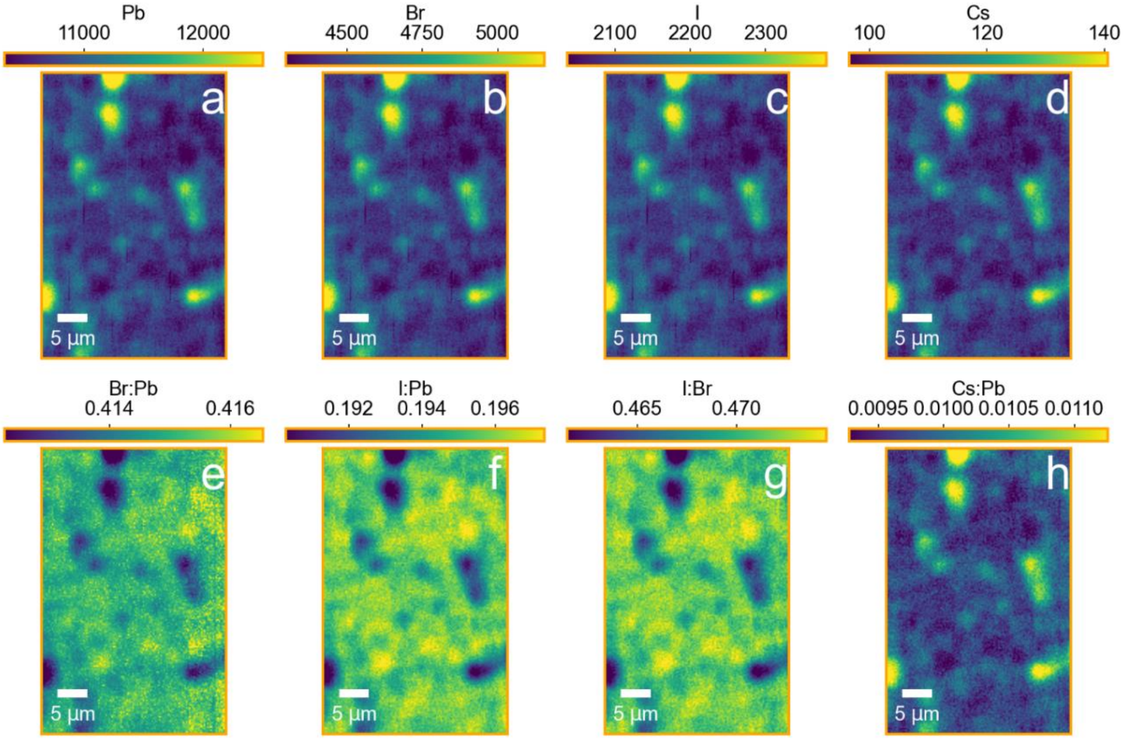

Supplementary Figure 50: nXRF maps of a 2PACz/TCTH solar cell. nXRF maps of the a) Pb L, b) Br K, c) I L and d) Cs L lines. Ratios of the e) Br:Pb lines, f) I:Pb lines, g) I:Br lines and h) Cs:Pb lines. Scalebars are 5 µm.

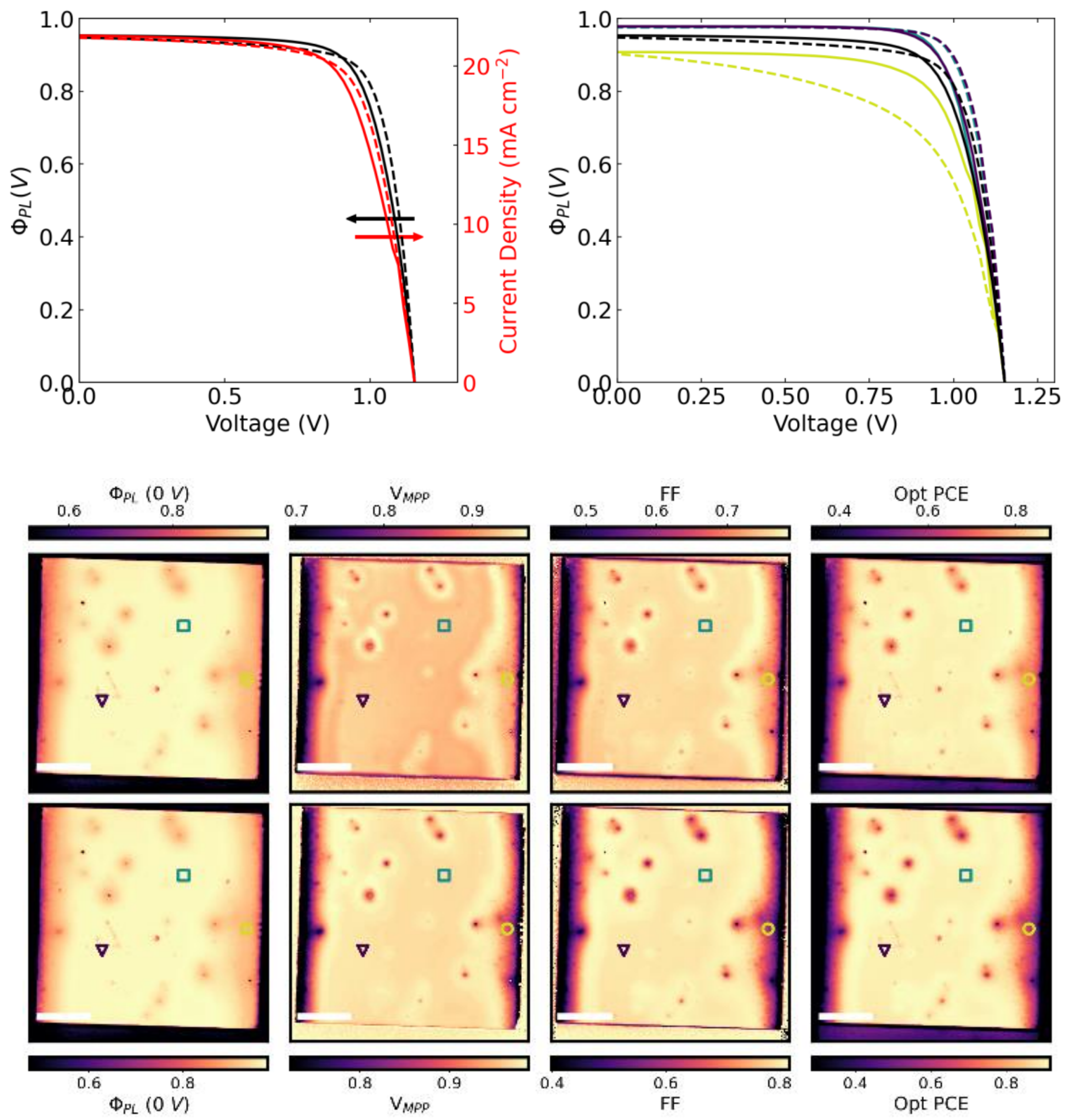

Supplementary Figure 51: Large scale optical JV measurements of a pristine 2PACz/DCDH perovskite solar cell. a) mean optical (black) and electrical (red) JV measurements of the device. b) Optical JV curves extracted from the areas marked in panels c-j. Maps of the optical JV figures of merit c) optical extraction efficiency, d) max power point voltage, e) fill factor, f) optical PCE extracted from the reverse scan. g) optical extraction efficiency, h) max power point voltage, i) fill factor, j optical PCE extracted from the forward scan. Scalebars are 1 mm. Solid lines are reverse voltage scans, dashed lines are forward scans. Coloured arrows point to their respective y-axes.

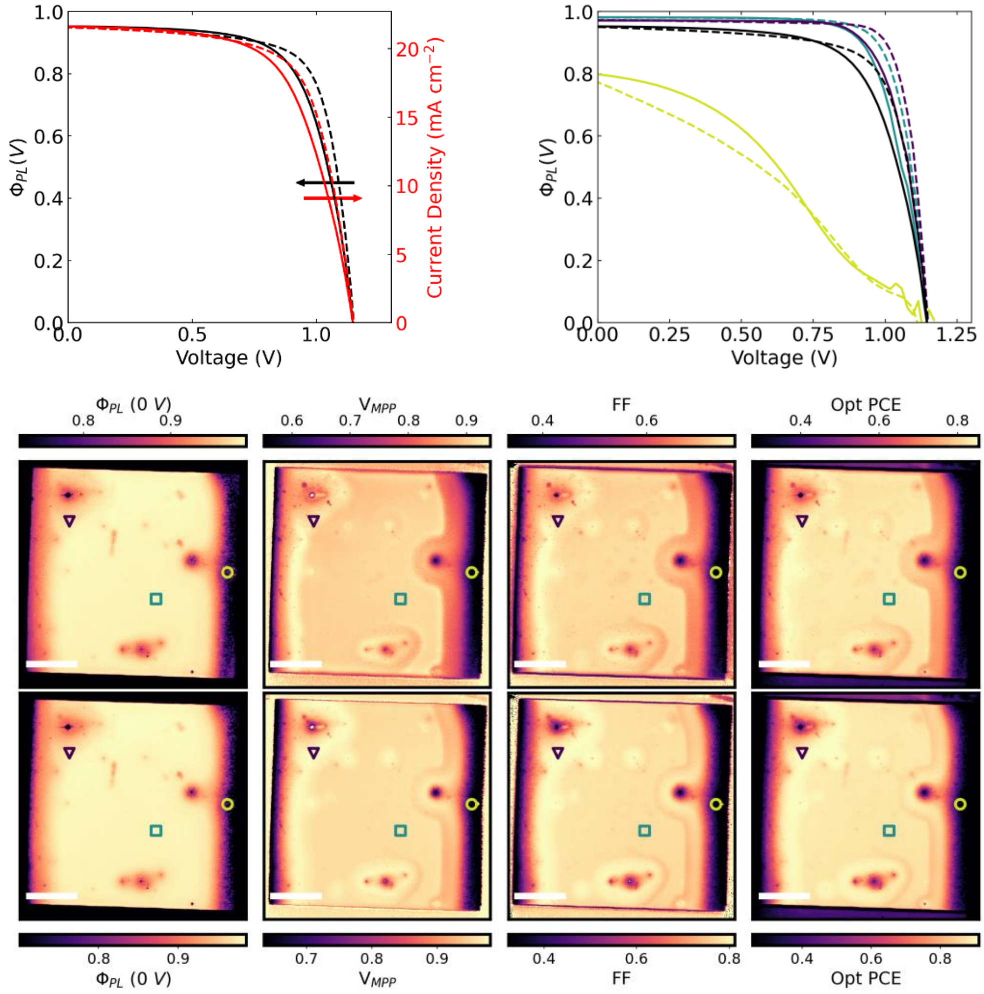

Supplementary Figure 52: Large scale optical JV measurements of pristine 2PACz/DCDH perovskite solar cell. a) mean optical (black) and electrical (red) JV measurements of the device. b) Optical JV curves extracted from the areas marked in panels c-j. Maps of the optical JV figures of merit c) optical extraction efficiency, d) max power point voltage, e) fill factor, f) optical PCE extracted from the reverse scan. g) optical extraction efficiency, h) max power point voltage, i) fill factor, j optical PCE extracted from the forward scan. Scalebars are 1 mm. Solid lines are reverse voltage scans, dashed lines are forward scans. Coloured arrows point to their respective y-axes.

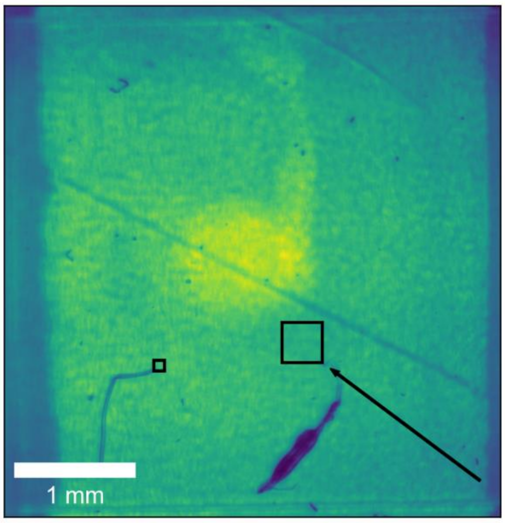

Supplementary Figure 53: Reflection image of the DCDH device shown in Figure 2 with the large hysteresis. The large black square shows the area with pronounced hysteresis shown in the main text, while the small area shows the area with lesser hysteresis further away from the degradation front depicted by the arrow. Scalebar is 1 mm.

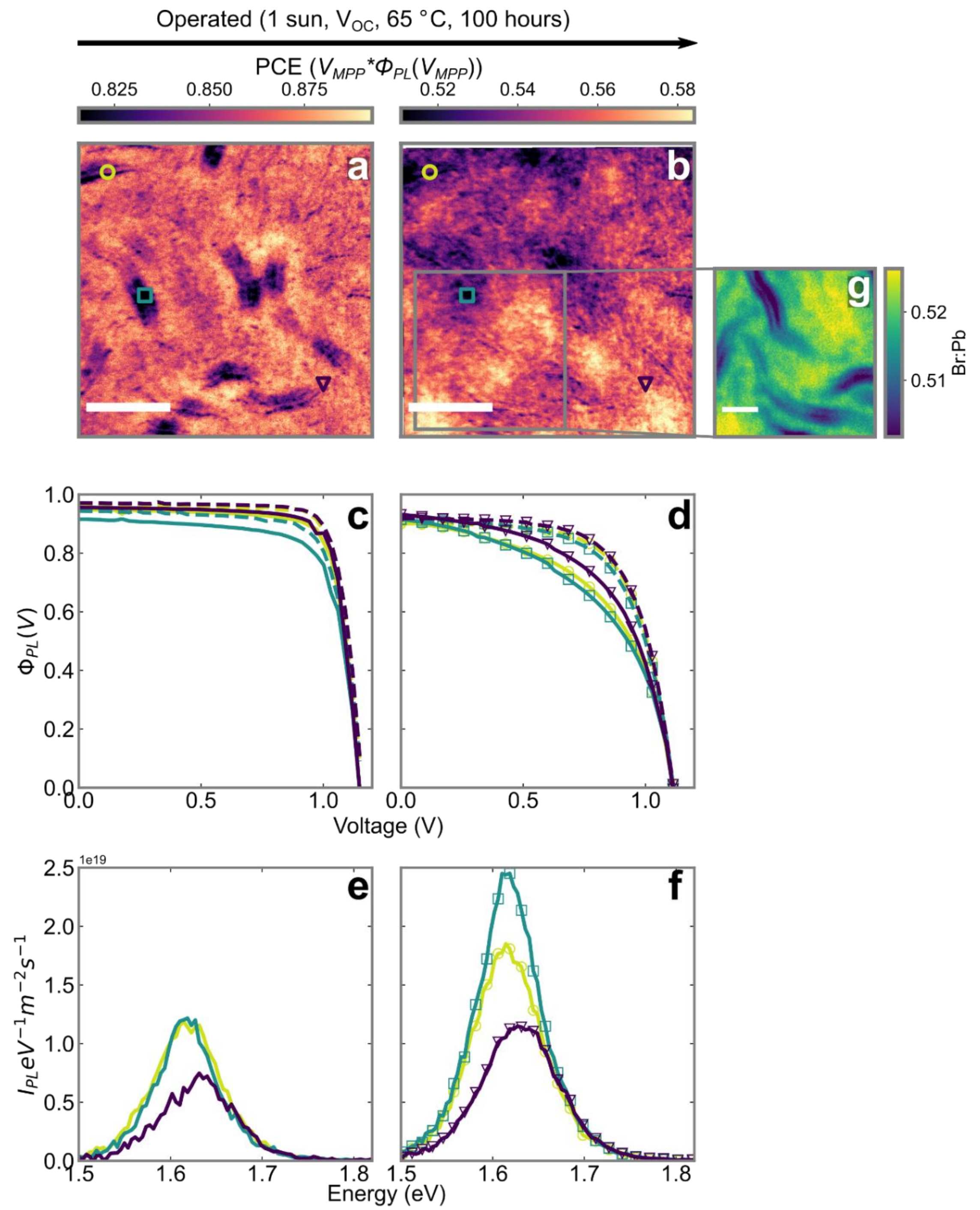

Supplementary Figure 54: Optical and electronic spectra of a 2PACz/DCDH solar cell before and after operation away from an edge. Optical PCE maps of the same area of a fresh a) and operated b) DCDH solar cell after 100 hours at V_{OC}, 65 °C and 1 sun illumination. c) and d) Optical JV curves before and after ageing from the points marked in panels a and b. Solid lines are reverse scans, dashed lines are forward scans. e) and f) PL spectra from the same marked areas before and after ageing respectively. g) Br:Pb map of region marked in panel b consistent with relatively unchanged perovskite chemical character post accelerated ageing. Scalebars in a and b are 25 µm, scalebar in g is 10 µm.

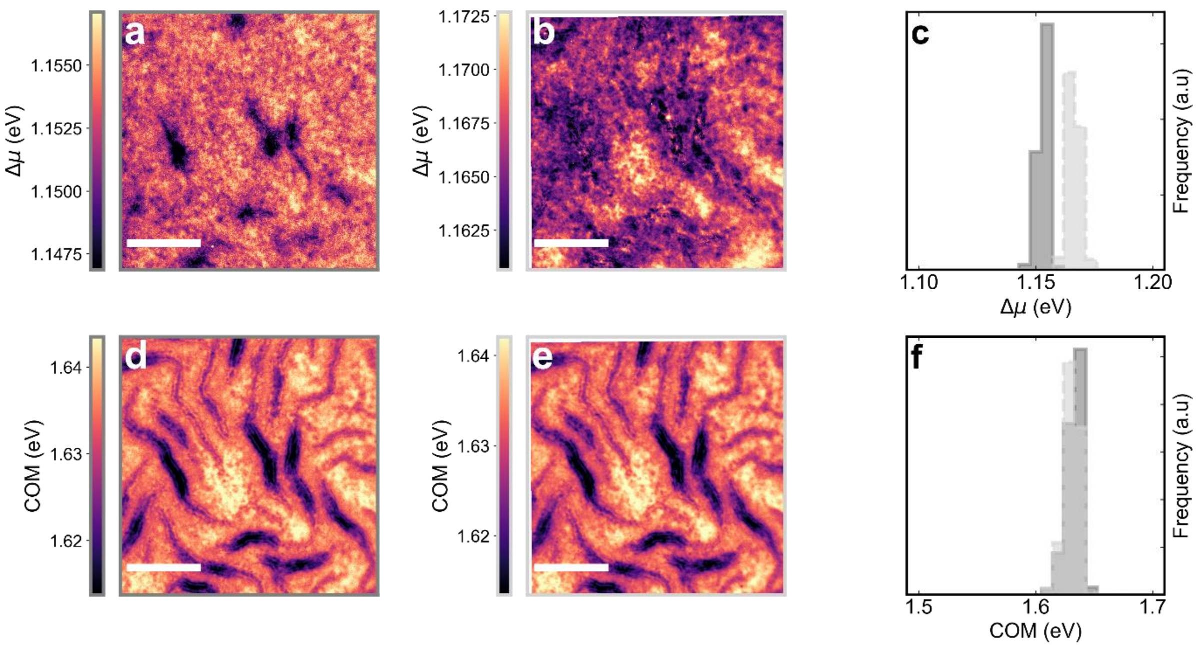

Supplementary Figure 55: Δμ and COM of a 2PACz/DCDH solar cell before after operational stress away from an edge. Δμ a) before and b) after accelerated ageing, distributions summarised in panel c) where the solid gray and dashed gray histograms show before and after stress testing respectively. COM d) before and e) after accelerated ageing, distributions summarised in panel f). The scan area is the same as for the voltage dependent measurement shown in Supplementary Figure 47. Scalebars are 25 μm.

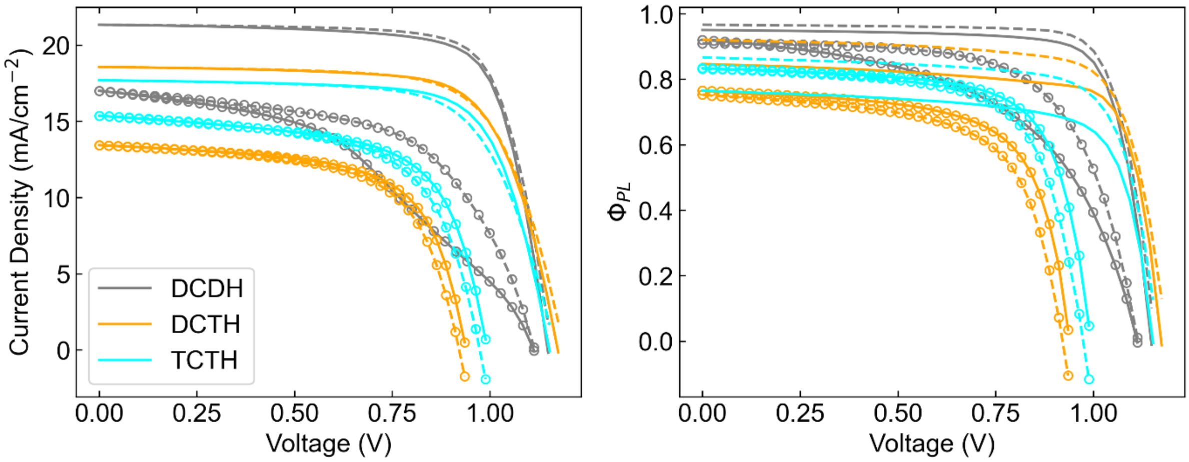

Supplementary Figure 56: Averaged electrical and optoelectronic properties of perovskite compositional series before and after operation. a) Shows the average electronic JV curves for the perovskite compositional series before (no markers) and after (markers) operation. b) Shows the spatially averaged optical JV curves for the perovskite compositional series before (no markers) and after (markers) operation. c) Shows the average open circuit PL spectra for the perovskite compositional series before (no markers) and after (markers) operation.

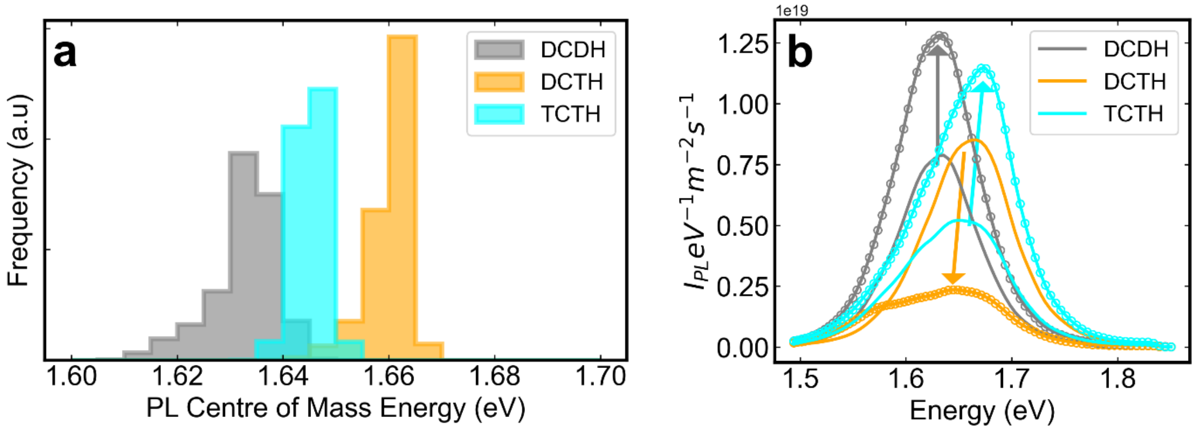

Supplementary Figure 57: PL spectral characteristics of perovskite compositional series. a) Histograms of PL COM energies extracted at each spatial point for DCDH (grey), DCTH (orange) and TCTH (cyan) perovskite solar cells. b) Spatially averaged PL spectra of devices before (solid line no markers) and after accelerated ageing (solid line with markers).

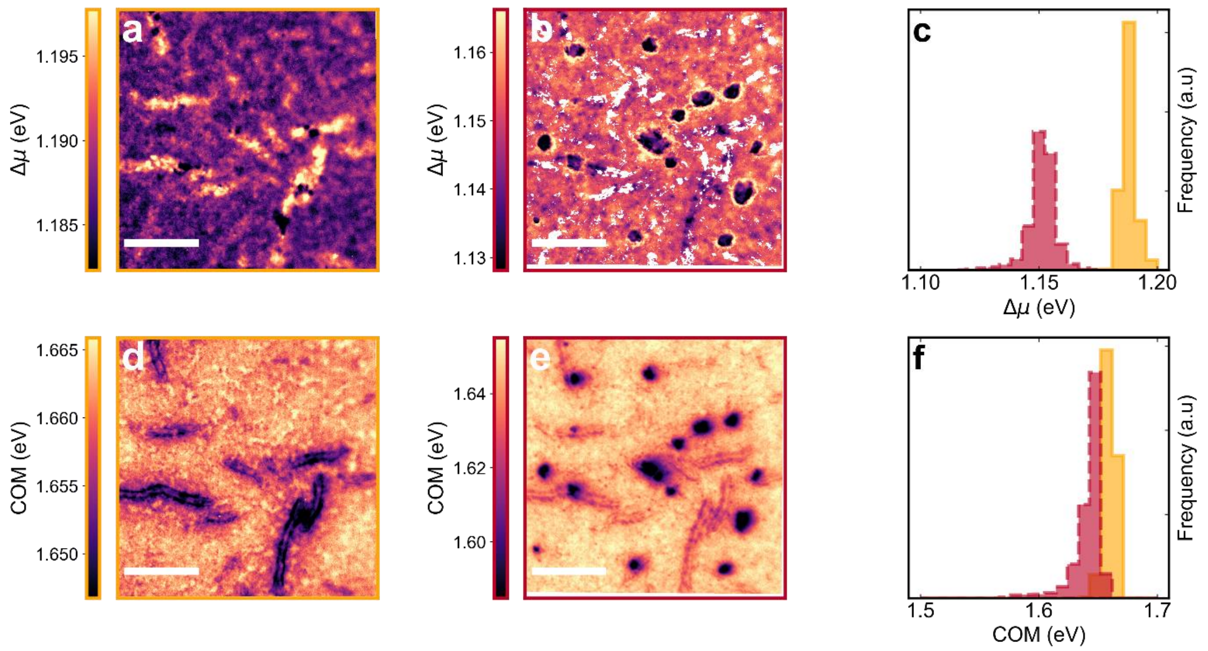

Supplementary Figure 58: Δμ and COM of a 2PACz/DCTH solar cell before after operational stress test. Δμ a) before and b) after accelerated ageing, distributions summarised in panel c) where the orange and red histograms show before and after stress testing respectively. COM d) before and e) after accelerated ageing, distributions summarised in panel f). The scan area is the same as for the voltage dependent measurement shown in Figure 3. Scalebars are 25 μm.

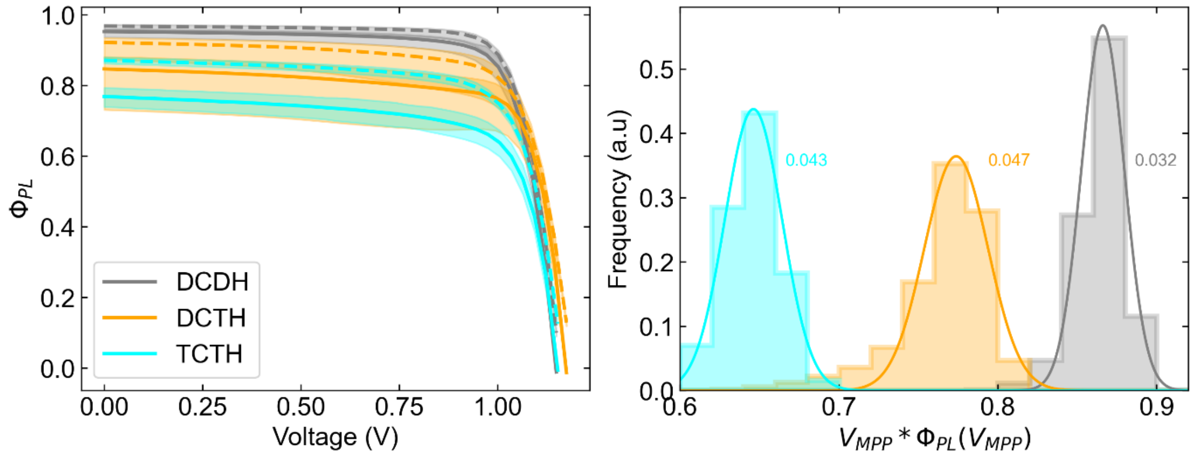

Supplementary Figure 59: Spatial PCE heterogeneity as a function of perovskite composition. a) Reverse (solid lines) and forward (dotted lines) spatially averaged optical JV curves. Shaded areas indicate the 5th and 95th percentile optical PCE area. b) Histograms of optical PCE distributions for a range of perovskite compositions.

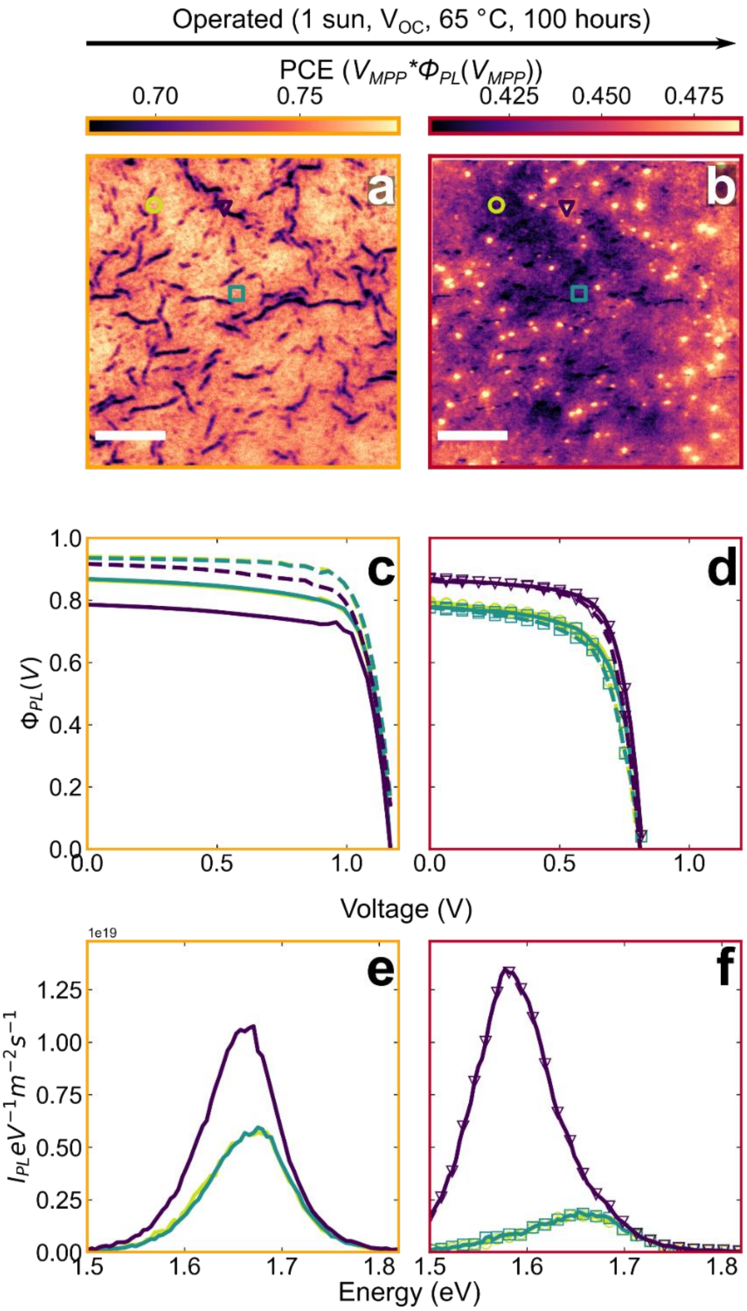

Supplementary Figure 60: Optical and electronic spectra of a 2PACz/DCTH solar cell before and after operational stress test. Optical PCE maps of the same area of a fresh a) and operated b) DCTH solar cell after 100 hours at V_{OC}, 65 °C and 1 sun illumination. c) and d) Optical JV curves before and after ageing from the points marked in panels a and b. Solid lines are reverse scans, dashed lines are forward scans. e) and f) PL spectra from the same marked areas before and after ageing respectively. g) Br:Pb map of region marked in panel b consistent with relatively unchanged perovskite chemical character post accelerated ageing. Scalebars in a and b are 75 µm.

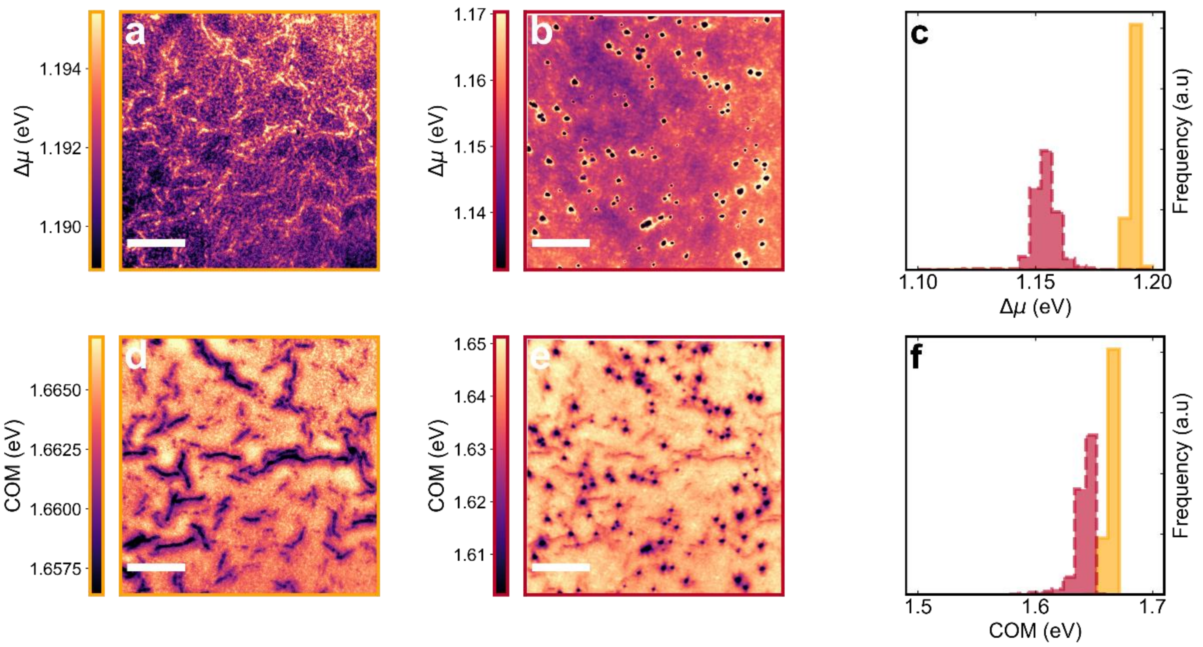

Supplementary Figure 61: Δμ and COM of a 2PACz/DCTH solar cell before after accelerated ageing. Δμ a) before and b) after accelerated ageing, distributions summarised in panel c) where the orange and red histograms show before and after stress testing respectively. COM d) before and e) after accelerated ageing, distributions summarised in panel f). The scan area is the same as for the voltage dependent measurement shown in Supplementary Figure 21. Scalebars are 75 μm.

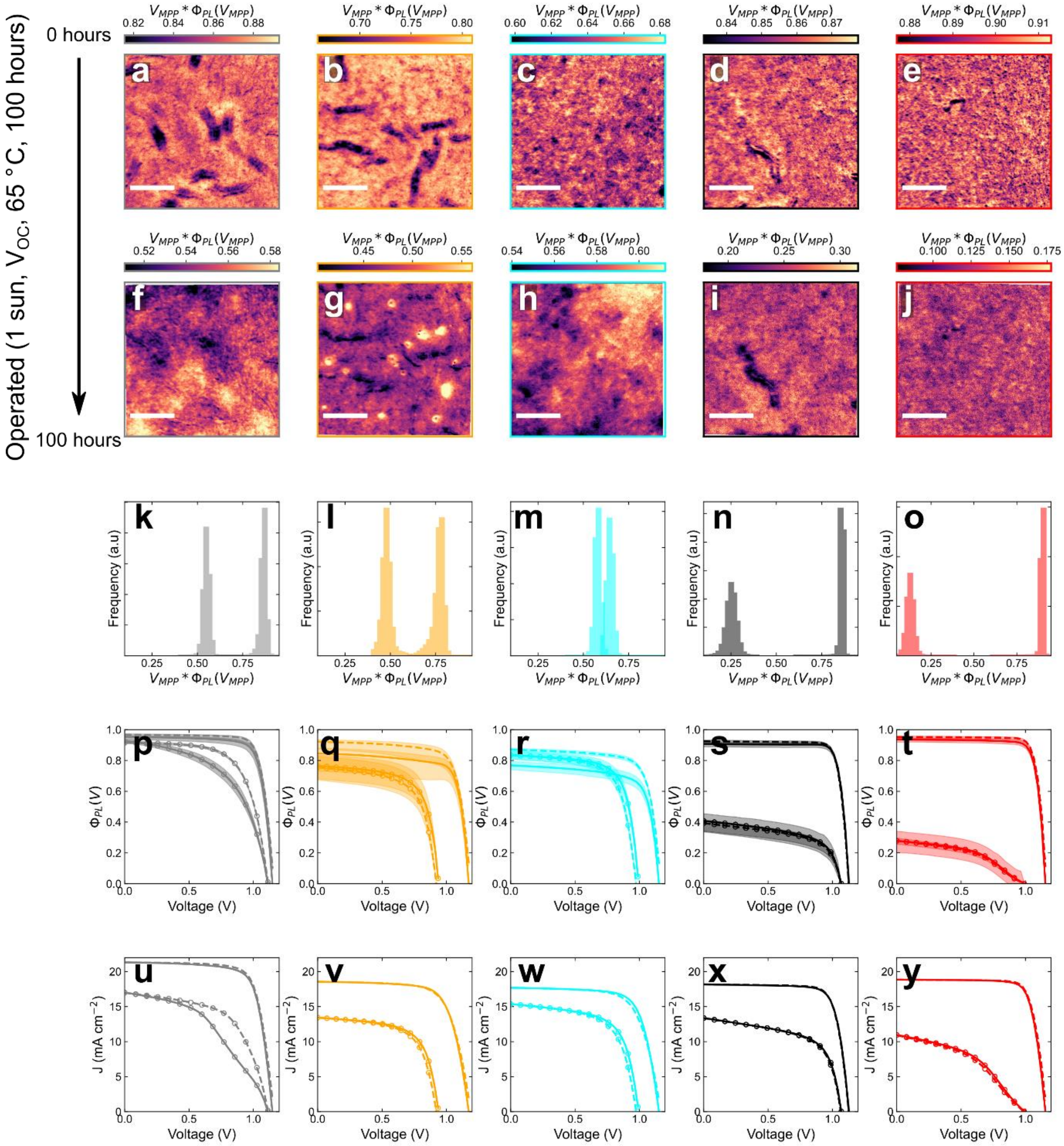

Supplementary Figure 62: Summary of operational degradation observation across the device phase space. Optical PCE maps of a) 2PACz/DCDH, b) 2PACz/DCTH, c) 2PACz/TCTH, d) MeO-2PACz/TCTH and e) Me-4PACz/TCTH before accelerated stress test. Optical PCE maps of f) 2PACz/DCDH, g) 2PACz/DCTH, h) 2PACz/TCTH, i) MeO-2PACz/TCTH and j) Me-4PACz/TCTH after accelerated stress test. Optical PCE histograms of k) 2PACz/DCDH, l) 2PACz/DCTH, m) 2PACz/TCTH, n) MeO-2PACz/TCTH and o) Me-4PACz/TCTH before and after (cross-hatched) the accelerated stress test. Optical JV curves of p) 2PACz/DCDH, q) 2PACz/DCTH, r) 2PACz/TCTH, s) MeO-2PACz/TCTH and t) Me-4PACz/TCTH before (no markers) and after(markers) the accelerated stress test. The intervals indicate the spatial variation in the optical JV curves across the regions measured. Electrical JV curves of u) 2PACz/DCDH, v) 2PACz/DCTH, w) 2PACz/TCTH, x) MeO-2PACz/TCTH

and y) Me-4PACz/TCTH before (no markers) and after (markers) the accelerated stress test. Shaded areas show the distribution of JV curves across the scanned area.

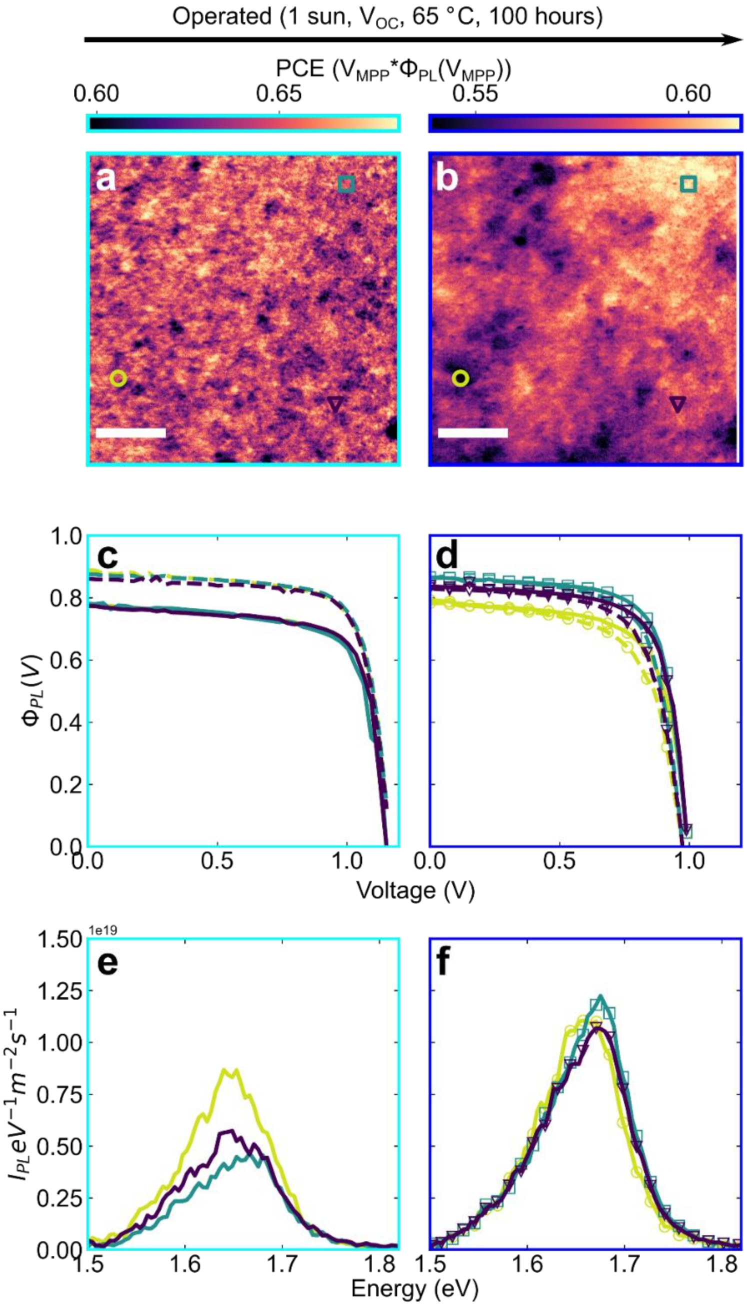

Supplementary Figure 63: Optical and electronic spectra of 2PACz/TCTH solar cell at high magnification before and after operational stress test. Optical PCE maps of the same area of a fresh a) and operated b) TCTH solar cell after 100 hours at V_{OC}, 65 °C and 1 sun illumination. c) and d) Optical JV curves before and after ageing from the points marked in panels a and b. Solid lines are reverse scans, dashed lines are forward scans. e) and f) PL spectra from the same marked areas before and after ageing respectively. Scalebars are 25 µm.

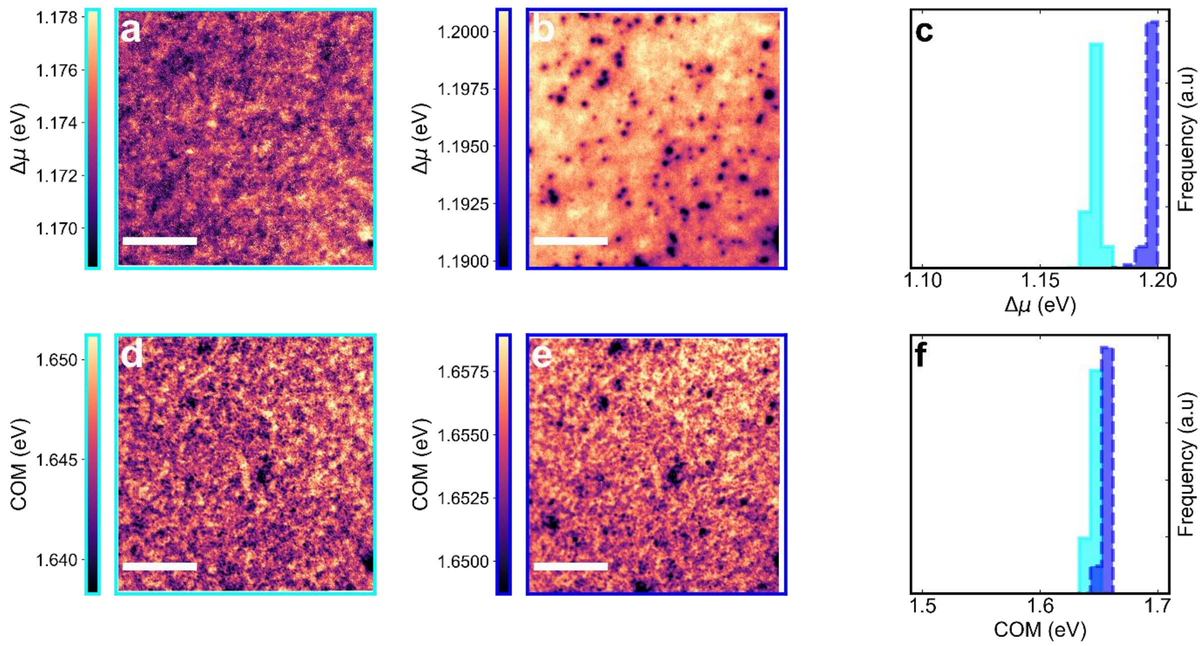

Supplementary Figure 64: Δμ and COM of a 2PACz/TCTH solar cell before and after operational stress test. Δμ a) before and b) after accelerated ageing, distributions summarised in panel c) where the cyan and blue histograms show before and after stress testing respectively. COM d) before and e) after accelerated ageing, distributions summarised in panel f). The scan area is the same as for the voltage dependent measurement shown in Supplementary Figure 26. Scalebars are 75 μm.

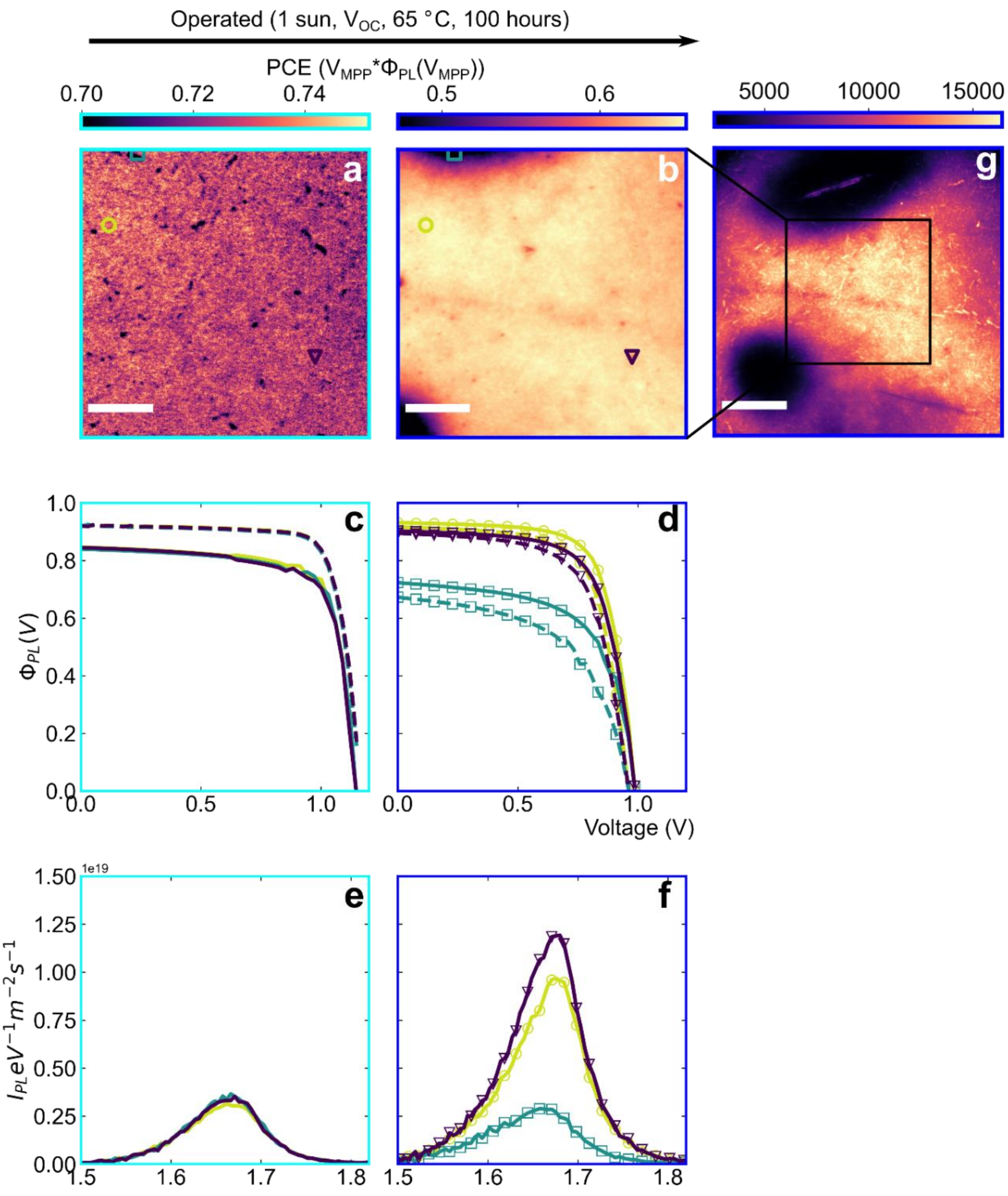

Supplementary Figure 65: Optical and electronic spectra of a 2PACz/TCTH solar cell at low magnification before and after operation away from an edge. Optical PCE maps of the same area of a fresh a) and operated b) TCTH solar cell after 100 hours at V_{OC}, 65 °C and 1 sun illumination. c) and d) Optical JV curves before and after ageing from the points marked in panels a and b. Solid lines are reverse scans, dashed lines are forward scans. e) and f) PL spectra from the same marked areas before and after ageing respectively. g) Large area broadband PL map at open circuit containing the region shown in panels a and b highlighting the large scale degradation features forming. Scalebars in a and b are 75 µm, scalebar in g is 150 µm.

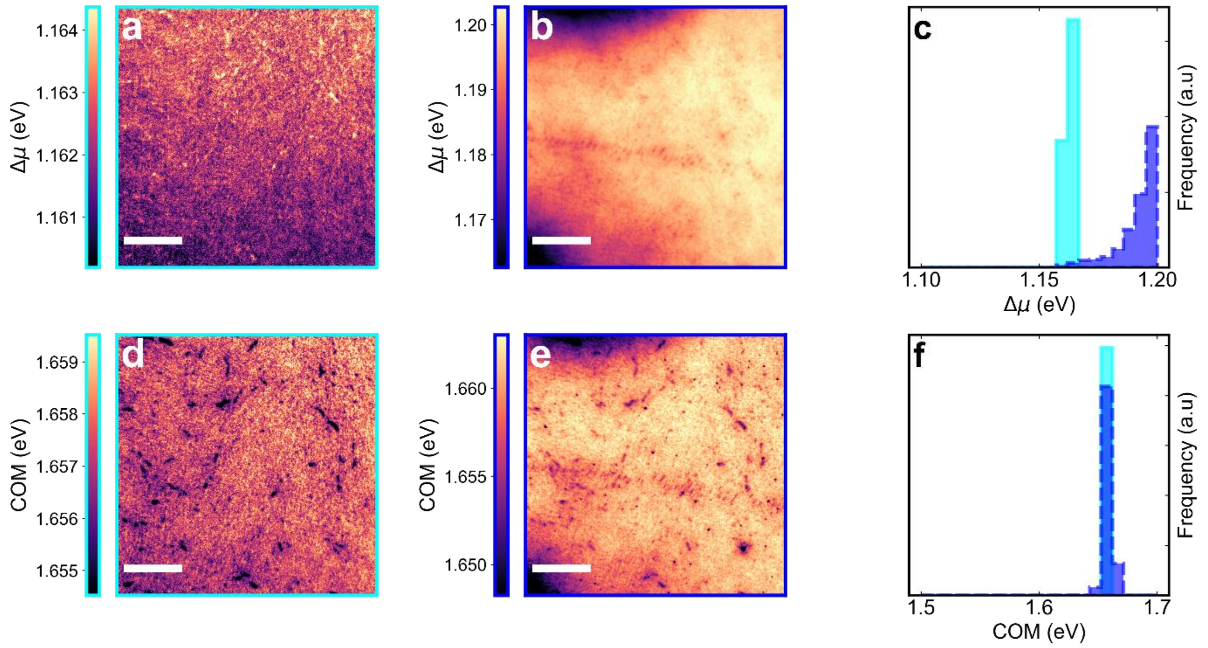

Supplementary Figure 66: Δμ and COM of a 2PACz/TCTH solar cell before after accelerated ageing. Δμ a) before and b) after accelerated ageing, distributions summarised in panel c) where the cyan and blue histograms show before and after stress testing respectively. COM d) before and e) after accelerated ageing, distributions summarised in panel f). The scan area is the same as for the voltage dependent measurement shown in Supplementary Figure 58. Scalebars are 75 μm.

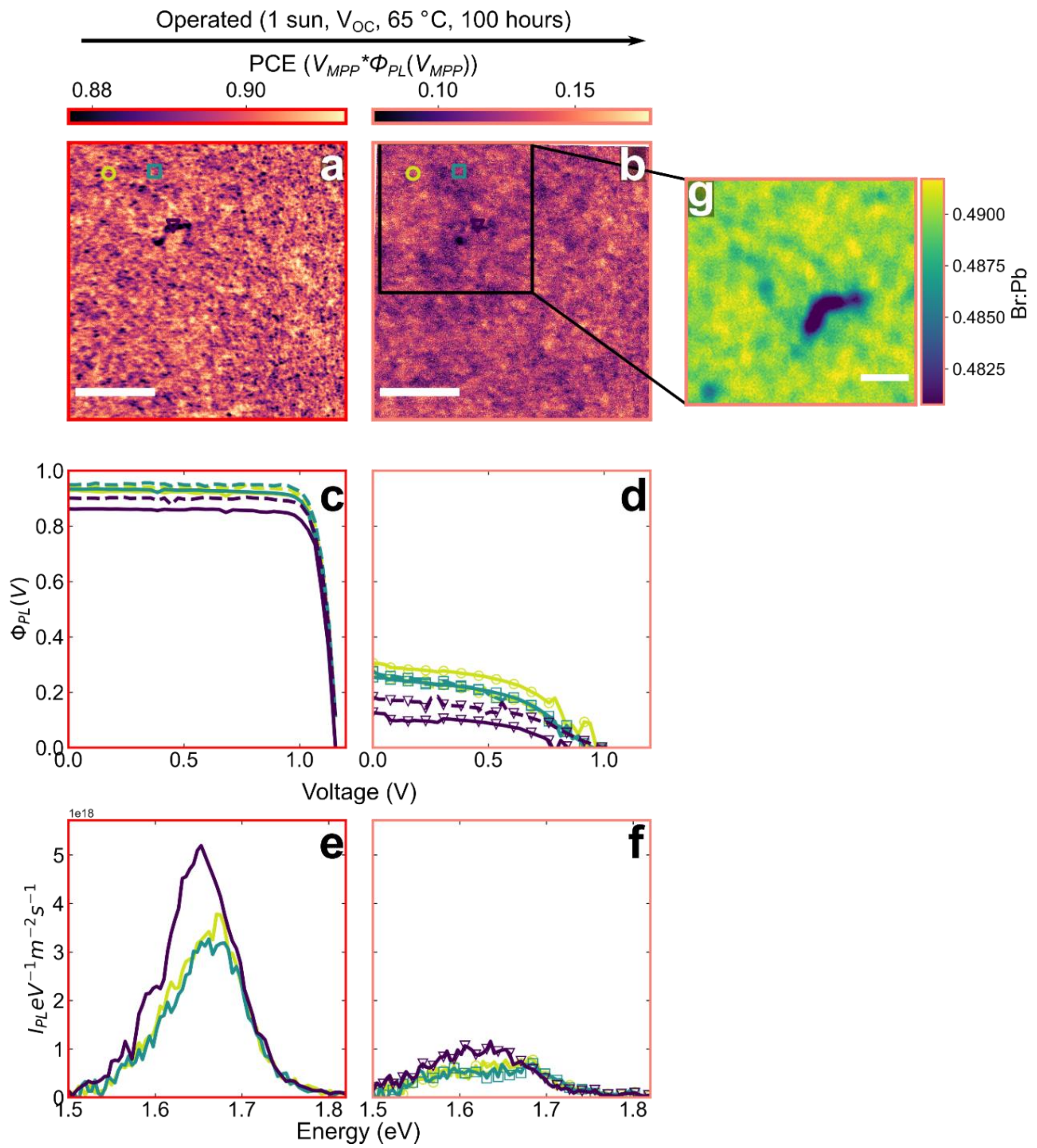

Supplementary Figure 67: Optical and electronic spectra of a Me-4PACz/TCTH solar cell before and after operation away from an edge. Optical PCE maps of the same area of a fresh a) and operated b) TCTH solar cell after 100 hours at V_{OC}, 65 °C and 1 sun illumination. c) and d) Optical JV curves before and after ageing from the points marked in panels a and b. Solid lines are reverse scans, dashed lines are forward scans. e) and f) PL spectra from the same marked areas before and after ageing respectively. g) Br:Pb map of region marked in panel b. Scalebars in a and b are 25 µm, scalebar in g is 10 µm.

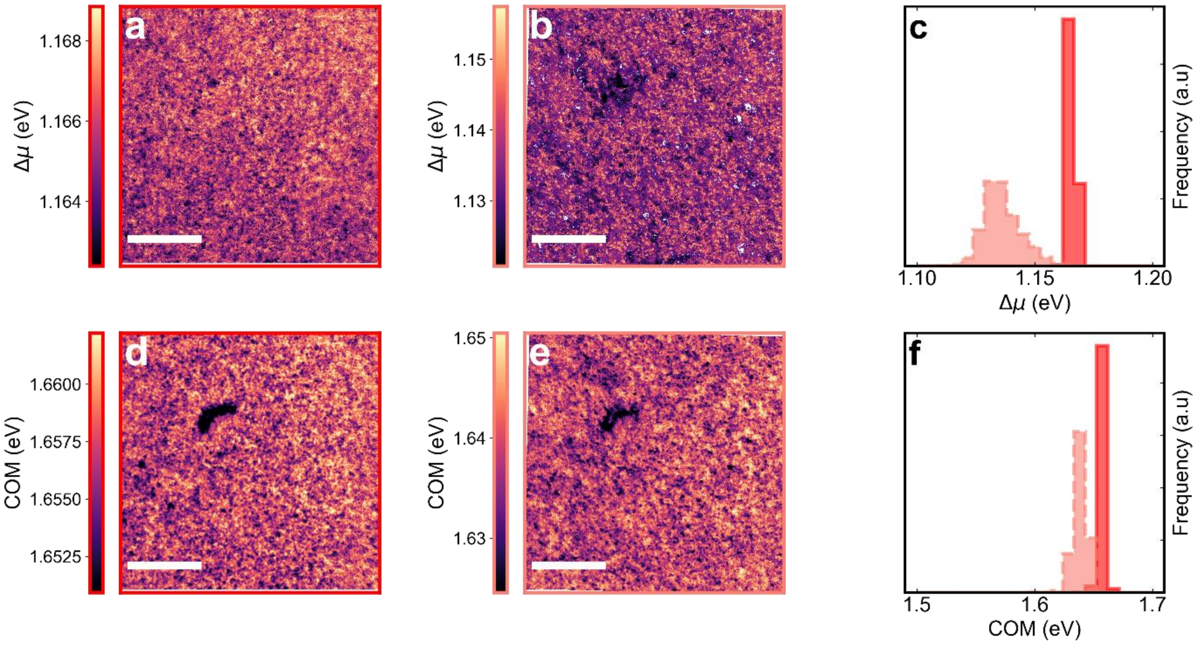

Supplementary Figure 68: Δμ and COM of a Me-4PACz/TCTH solar cell before and after operational stress test. Δμ a) before and b) after accelerated ageing, distributions summarised in panel c) where the red and pink histograms show before and after stress testing respectively. COM d) before and e) after accelerated ageing, distributions summarised in panel f). The scan area is the same as for the voltage dependent measurement shown in Supplementary Figure 60. Scalebars are 25 μm.

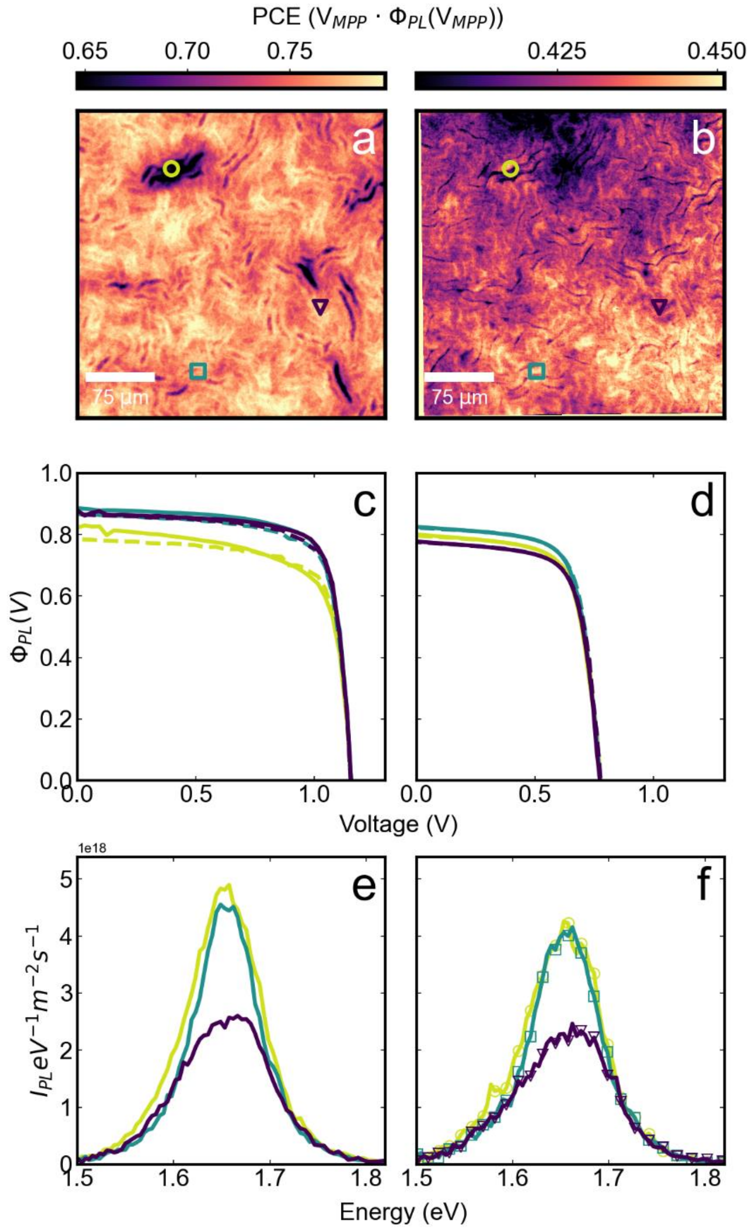

Supplementary Figure 69: Optical and electronic spectra of wrinkled 2PACz/TCTH solar cell before and after operational stress test. Optical PCE maps of the same area of a fresh a) and operated b) TCTH solar cell after 100 hours at V_{OC}, 65 °C and 1 sun illumination. c) and d) Optical JV curves before and after ageing from the points marked in panels a and b. Solid lines are reverse scans, dashed lines are forward scans. e) and f) PL spectra from the same marked areas before and after ageing respectively.

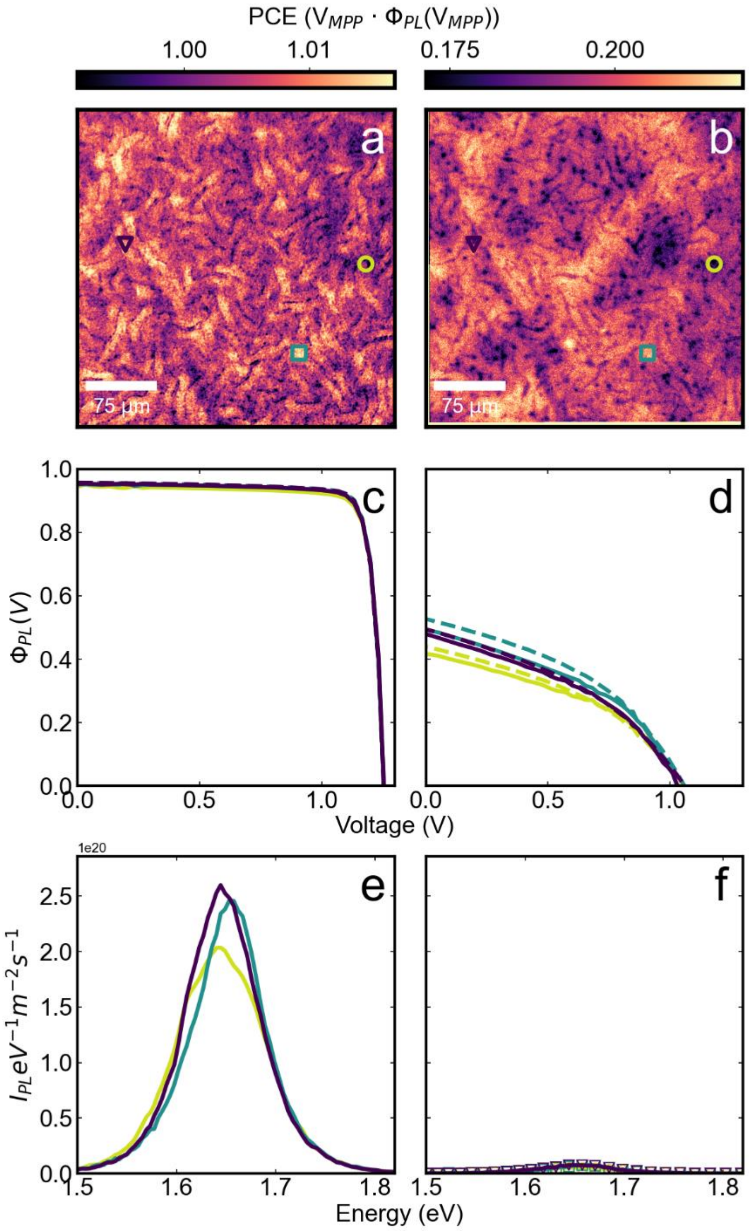

Supplementary Figure 70: Optical and electronic spectra of a 2PACz/TCTH solar cell passivated with PI before and after operational stress test. Optical PCE maps of the same area of a fresh a) and operated b) PI passivated TCTH solar cell after 100 hours at V_{OC}, 65 °C and 1 sun illumination. c) and d) Optical JV curves before and after ageing from the points marked in panels a and b. Solid lines are reverse scans, dashed lines are forward scans. e) and f) PL spectra from the same marked areas before and after ageing respectively.

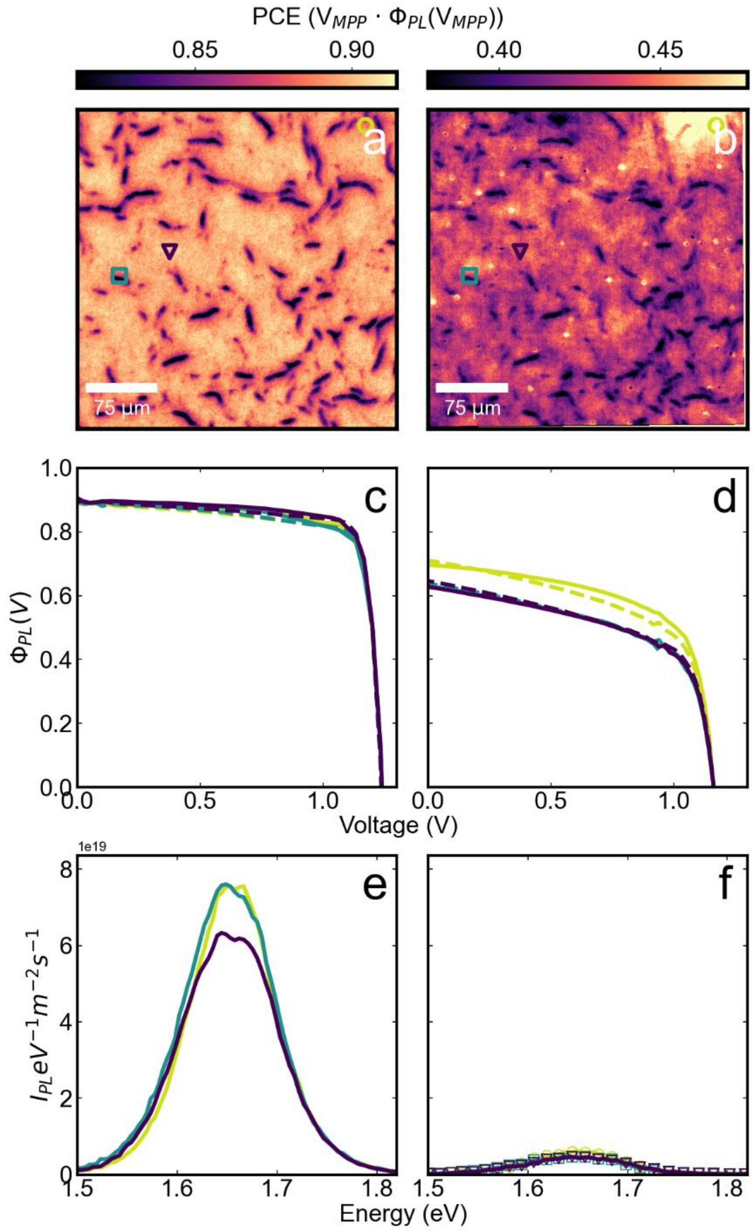

Supplementary Figure 71: Optical and electronic spectra of 2PACz/TCTH solar cell passivated with LiF before and after operational stress test. Optical PCE maps of the same area of a fresh a) and operated b) PI passivated TCTH solar cell after 100 hours at V_{OC}, 65 °C and 1 sun illumination. c) and d) Optical JV curves before and after ageing from the points marked in panels a and b. Solid lines are reverse scans, dashed lines are forward scans. e) and f) PL spectra from the same marked areas before and after ageing respectively.

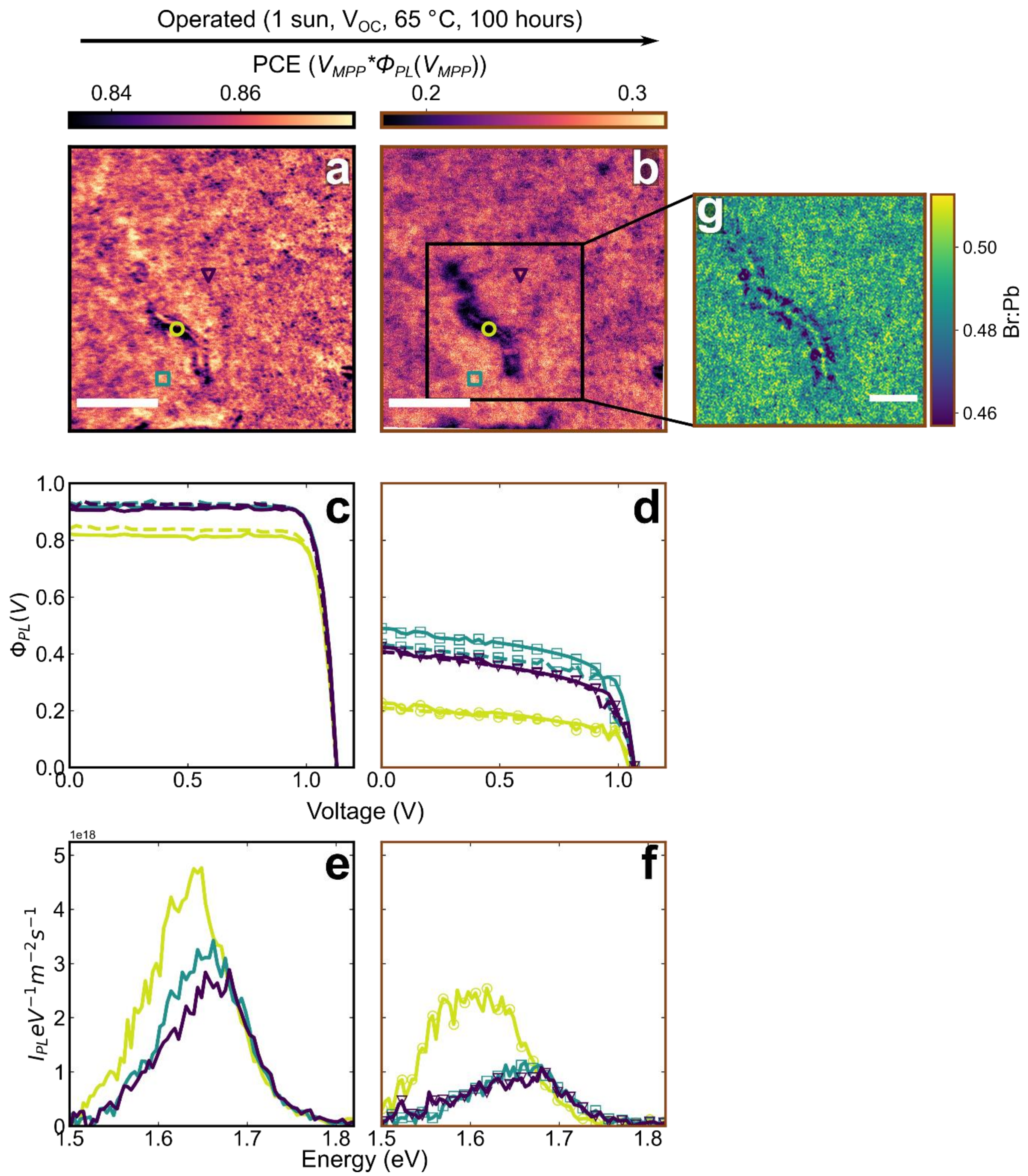

Supplementary Figure 72: Optical and electronic spectra of MeO-2PACz/TCTH solar cell before and after operational stress test. Optical PCE maps of the same area of a fresh a) and operated b) TCTH solar cell after 100 hours at V_{OC}, 65 °C and 1 sun illumination. c) and d) Optical JV curves before and after ageing from the points marked in panels a and b. Solid lines are reverse scans, dashed lines are forward scans. e) and f) PL spectra from the same marked areas before and after ageing respectively. g) Br:Pb map of region marked in panel b. Scalebars in a and b are 25 µm, scalebar in g is 10 µm.

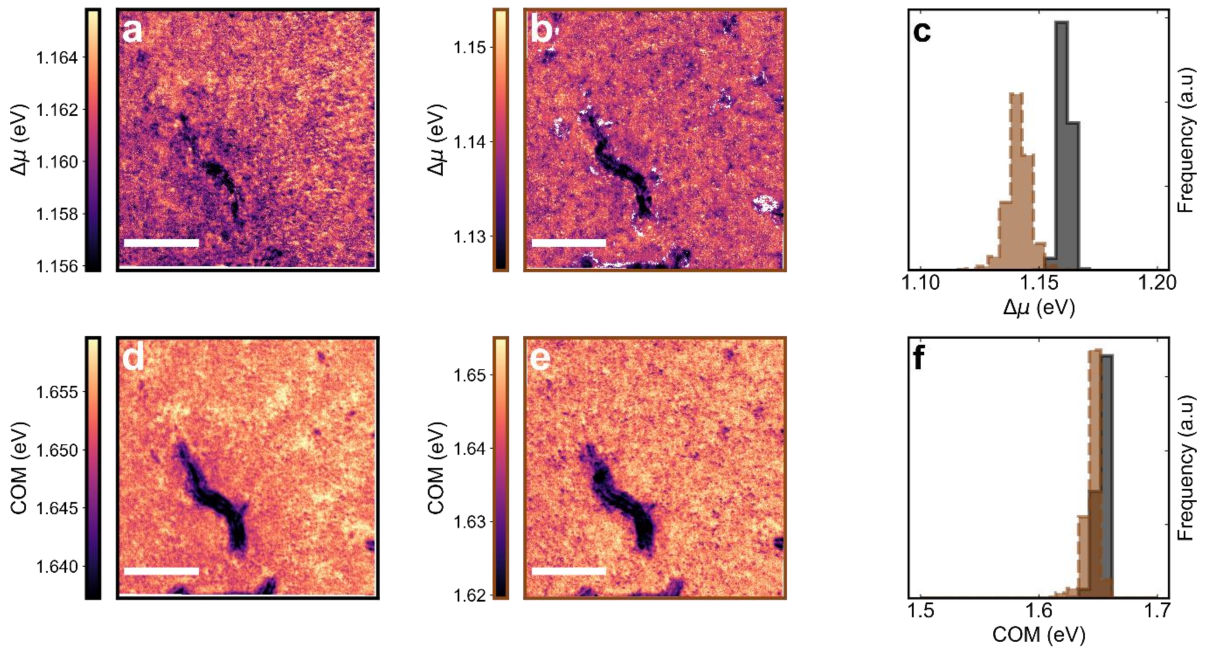

Supplementary Figure 73: Δμ and COM of MeO-2PACz/TCTH solar cell before and after operational stress test. Δμ a) before and b) after accelerated ageing, distributions summarised in panel c) where the black and brown histograms show before and after stress testing respectively. COM d) before and e) after accelerated ageing, distributions summarised in panel f). The scan area is the same as for the voltage dependent measurement shown in Supplementary Figure 65. Scalebars are 25 μm.

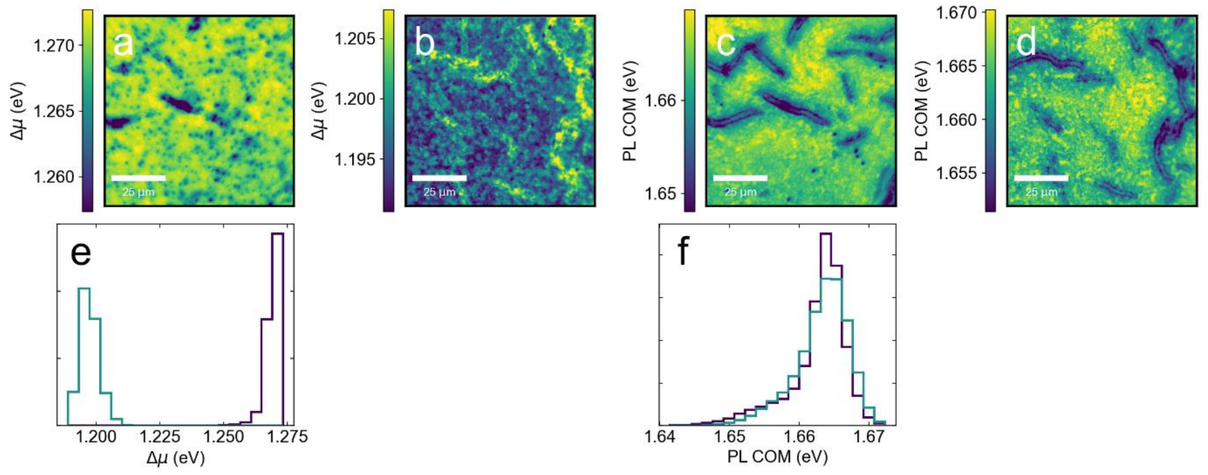

Supplementary Figure 74: Comparison of optical properties of reference and PI passivated 2PACz/TCTH devices. Δμ maps of the a) passivated and b) unpassivated 2PACz/DCTH devices. COM maps of the c) passivated and d) unpassivated devices. Histograms of the e) Δμ and f) COM distributions comparing passivated (blue) and unpassivated (purple).

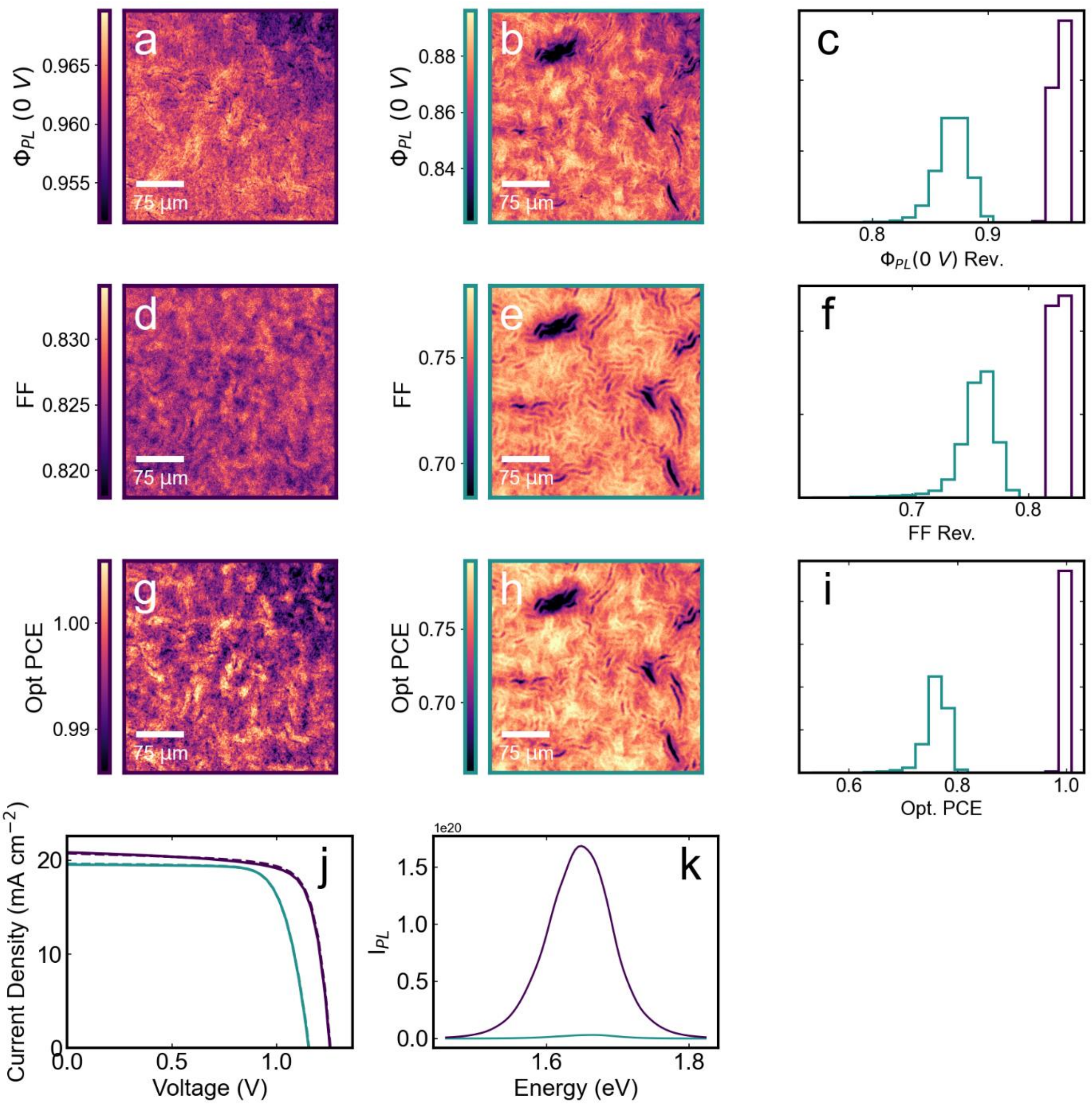

Supplementary Figure 75: **Microscopic comparison of reference and PI passivated 2PACz/TCTH devices.** Comparison of reference and PI passivated 2PACz/TCTH device. Maps of the optical extraction efficiency for a) PI passivated and b) reference devices. c) Histograms of the spatial distributions shown in a and b. Maps of the optical fill factor for d) PI passivated and e) reference devices. f) Histograms of the spatial distributions shown in d and e. Maps of the optical PCE for g) PI passivated and h) reference devices. i) Histograms of the spatial distributions shown in g and h. Comparisons of the j) electrical JV curves and k) spatially averaged PL spectra of the PI passivated (purple) and reference (blue) devices.

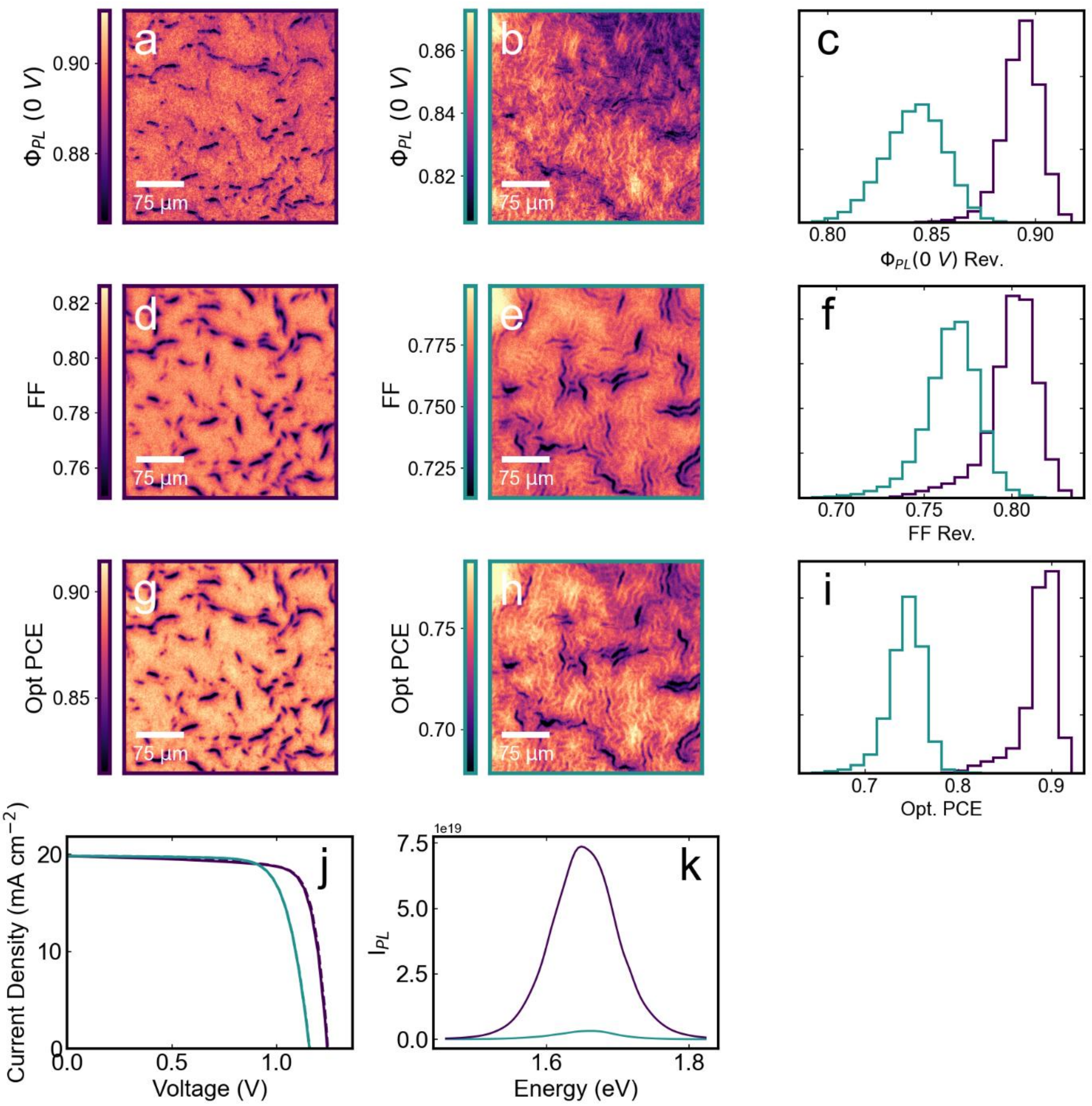

Supplementary Figure 76: **Microscopic comparison of reference and LiF passivated 2PACz/TCTH devices.** Comparison of reference and LiF passivated 2PACz/TCTH device. Maps of the optical extraction efficiency for a) LiF passivated and b) reference devices. c) Histograms of the spatial distributions shown in a and b. Maps of the optical fill factor for d) LiF passivated and e) reference devices. f) Histograms of the spatial distributions shown in d and e. Maps of the optical PCE for g) PI passivated and h) reference devices. i) Histograms of the spatial distributions shown in g and h. Comparisons of the j) electrical JV curves and k) spatially averaged PL spectra of the LiF passivated (purple) and reference (blue) devices. Solid lines are reverse voltage scans, dashed lines are forward scans.

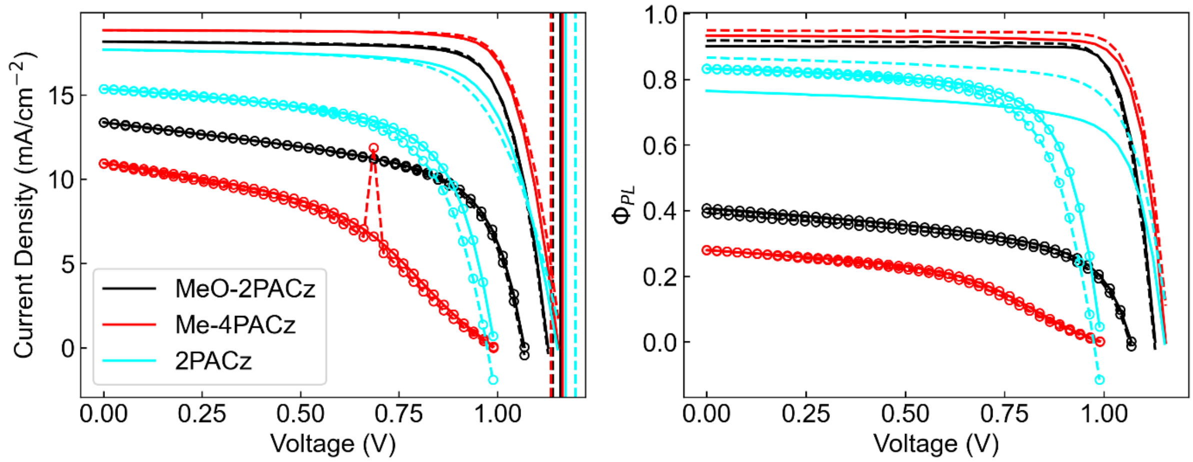

Supplementary Figure 77: Averaged electrical and optoelectronic properties of the HTL series before and after operation. a) Shows the average electronic JV curves for the HTL series before (no markers) and after (markers) operation. b) Shows the spatially averaged optical JV curves for the HTL series before (no markers) and after (markers) operation. Solid lines are reverse voltage scans, dashed lines are forward scans.

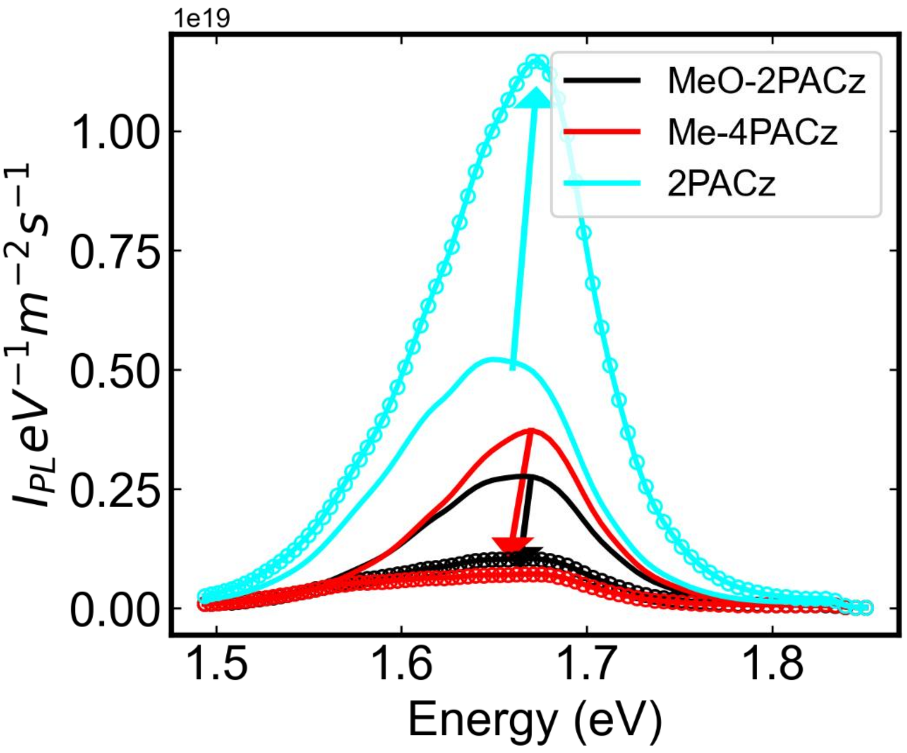

Supplementary Figure 78: Spatially averaged PL spectra at V_{OC} before (solid lines no markers), and after (solid lines with markers) accelerated ageing. All devices are TCTH with varying HTL layers.

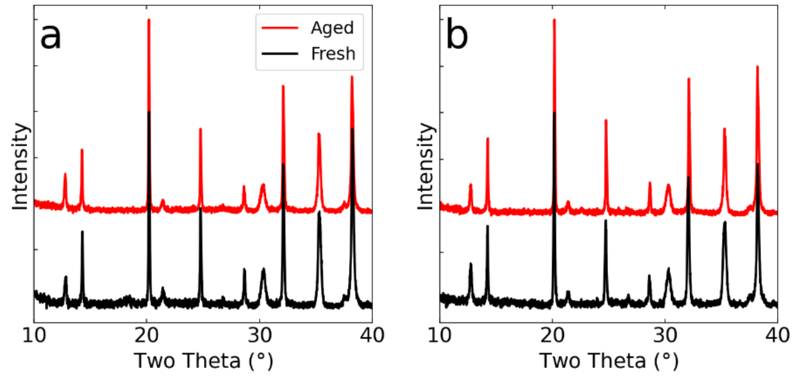

Supplementary Figure 79: Bulk XRD patterns of a) reference, b) PI passivated and c) LiF passivated 2PACz/TCTH devices before (black line) and after (red line) operational stress.

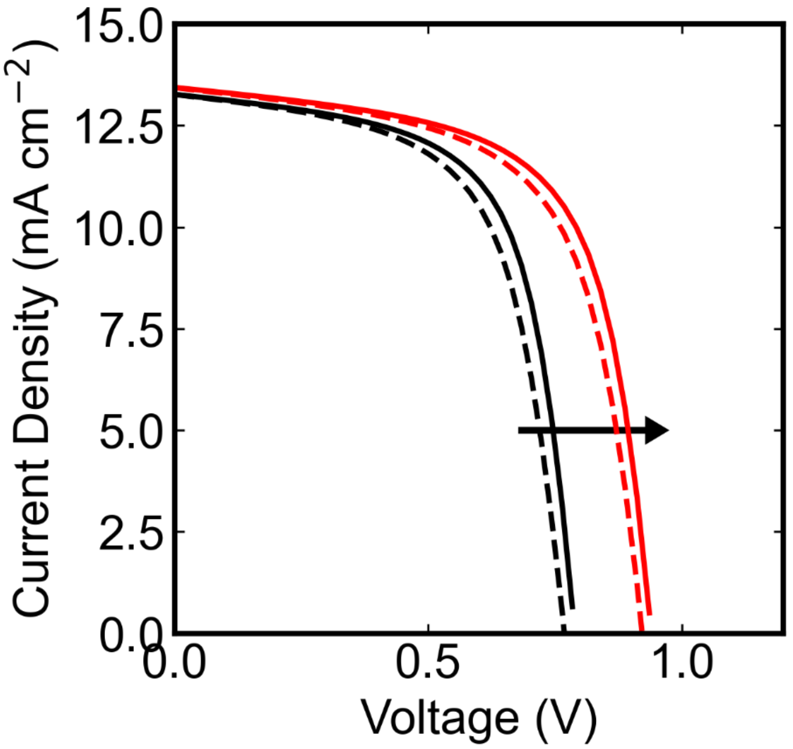

Supplementary Figure 80: Consecutive JV curves in DCTH perovskite solar cell. JV curves were measured ~1 hour apart with continuous light soaking showing that the V_{OC} is highly transient after accelerated ageing. Solid lines are reverse voltage scans, dashed lines are forward scans. Arrow is a guide to the eye indicating the direction in which time has moved forward.

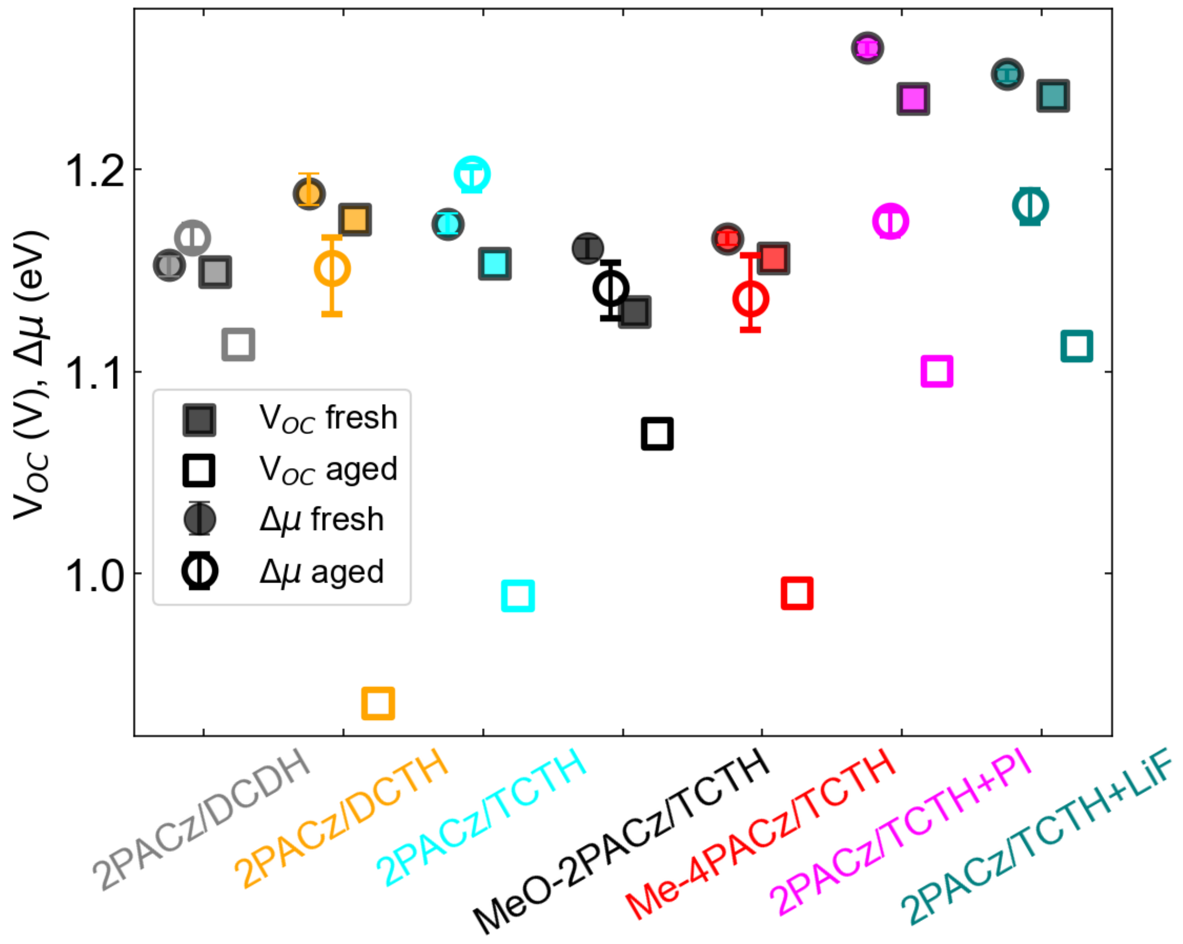

Supplementary Figure 81: Scatter plot of internal voltages (Δμ) and external voltages (V_{OC}) for representative devices of each type (n=1) before and after operational stress test. Error bars are standard deviations of values across the spatial area mapped in each case.

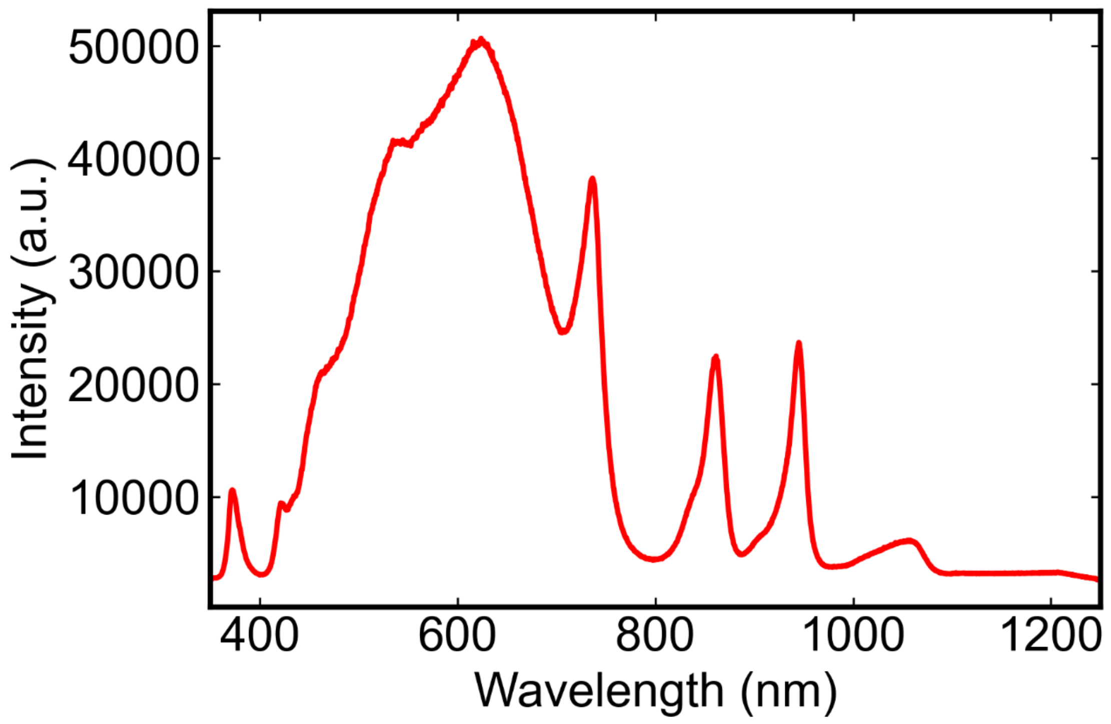

Supplementary Figure 82: Spectrum of solar simulator used for accelerated ageing.

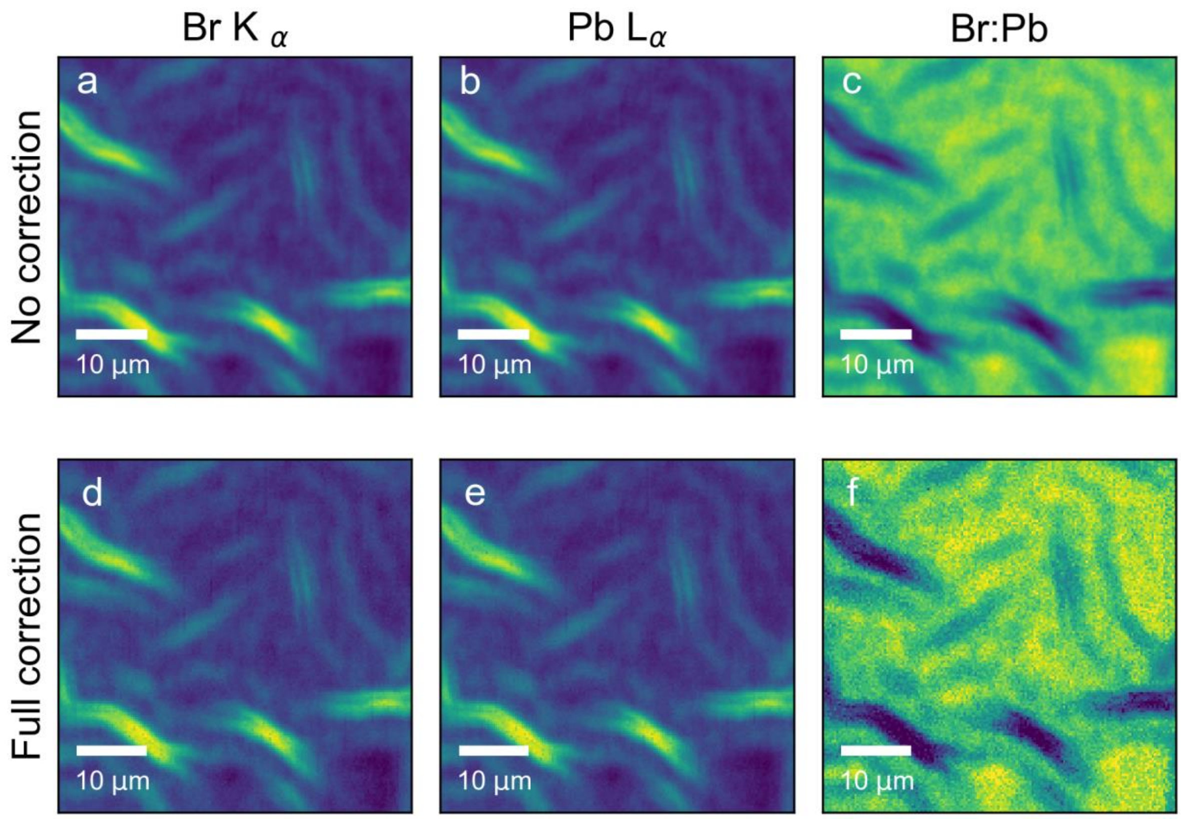

Supplementary Figure 83: **Self absorption corrected nXRF maps.** a) Br K_α, b) Pb L_α and c) Br:Pb ratio of a 2PACz/DCDH solar cell with uncorrected data. d) Br K_α, e) Pb L_α and f) Br:Pb ratio of a 2PACz/DCDH solar cell with fully corrected data.

Supplementary References

1 Lasher, G. & Stern, F. Spontaneous and Stimulated Recombination Radiation in Semiconductors. *Phys. Rev.* **133**, A553-A563, doi:10.1103/PhysRev.133.A553 (1964).

2 Wurfel, P. The chemical potential of radiation. *Journal of Physics C: Solid State Physics* **15**, 3967-3985, doi:10.1088/0022-3719/15/18/012 (1982).

3 Katahara, J. K. & Hillhouse, H. W. Quasi-Fermi level splitting and sub-bandgap absorptivity from semiconductor photoluminescence. *J. Appl. Phys.* **116**, 173504, doi:10.1063/1.4898346 (2014).

4 Braly, I. L., Stoddard, R. J., Rajagopal, A., Jen, A. K. Y. & Hillhouse, H. W. Photoluminescence and Photoconductivity to Assess Maximum Open-Circuit Voltage and Carrier Transport in Hybrid Perovskites and Other Photovoltaic Materials. *J. Phys. Chem. Lett.* **9**, 3779-3792, doi:10.1021/acs.jpclett.8b01152 (2018).

5 Frohna, K. *et al.* Nanoscale chemical heterogeneity dominates the optoelectronic response of alloyed perovskite solar cells. *Nat. Nanotechnol.* **17**, 190-196, doi:10.1038/s41565-021-01019-7 (2022).

6 Wagner, L. *et al.* Revealing fundamentals of charge extraction in photovoltaic devices through potentiostatic photoluminescence imaging. *Matter*, doi:10.1016/j.matt.2022.05.024 (2022).

7 Caprioglio, P. *et al.* On the Relation between the Open-Circuit Voltage and Quasi-Fermi Level Splitting in Efficient Perovskite Solar Cells. *Adv. Energy Mat.* **9**, 1901631, doi:10.1002/aenm.201901631 (2019).

8 Akel, S., Kulkarni, A., Rau, U. & Kirchartz, T. Relevance of Long Diffusion Lengths for Efficient Halide Perovskite Solar Cells. *PRX Energy* **2**, 013004, doi:10.1103/PRXEnergy.2.013004 (2023).

9 Caprioglio, P. *et al.* On the Origin of the Ideality Factor in Perovskite Solar Cells. *Adv. Energy Mat.* **10**, 2000502, doi:10.1002/aenm.202000502 (2020).

10 Burgelman, M., Nollet, P. & Degrave, S. Modelling polycrystalline semiconductor solar cells. *Thin Solid Films* **361-362**, 527-532, doi:10.1016/S0040-6090(99)00825-1 (2000).

11 Oliver, R. D. J. *et al.* Understanding and suppressing non-radiative losses in methylammonium-free wide-bandgap perovskite solar cells. *Energy Environ. Sci.* **15**, 714-726, doi:10.1039/D1EE02650J (2022).

12 Qin, D., Gu, P., Dhar, R. S., Razavipour, S. G. & Ban, D. Measuring the exciton diffusion length of C60 in organic planar heterojunction solar cells. *physica status solidi (a)* **208**, 1967-1971, doi:10.1002/pssa.201026724 (2011).

13 Dimitrov, S. D. *et al.* Singlet Exciton Lifetimes in Conjugated Polymer Films for Organic Solar Cells. *Polymers* **8**, doi:10.3390/polym8010014 (2016).

14 Diekmann, J. *et al.* Pathways toward 30% Efficient Single-Junction Perovskite Solar Cells and the Role of Mobile Ions. *Solar RRL* **5**, 2100219, doi:10.1002/solr.202100219 (2021).

15 Warby, J. *et al.* Understanding Performance Limiting Interfacial Recombination in pin Perovskite Solar Cells. *Adv. Energy Mat.* **12**, 2103567, doi:10.1002/aenm.202103567 (2022).

16 Stolterfoht, M. *et al.* The impact of energy alignment and interfacial recombination on the internal and external open-circuit voltage of perovskite solar cells. *Energy Environ. Sci.* **12**, 2778-2788, doi:10.1039/C9EE02020A (2019).

17 de la Peña, F. *et al.* Hyperspy 1.6.5. (2021).

18 Tustison, N. J. *et al.* Large-scale evaluation of ANTs and FreeSurfer cortical thickness measurements. *NeuroImage* **99**, 166-179, doi:10.1016/j.neuroimage.2014.05.044 (2014).

19 Avants, B. B. *et al.* A reproducible evaluation of ANTs similarity metric performance in brain image registration. *NeuroImage* **54**, 2033-2044, doi:10.1016/j.neuroimage.2010.09.025 (2011).

20 Bush, K. A. *et al.* Controlling Thin-Film Stress and Wrinkling during Perovskite Film Formation. *ACS Energy Lett.* **3**, 1225-1232, doi:10.1021/acsenergylett.8b00544 (2018).
