# Supplementary material for: The impact of interfacial quality and nanoscale performance disorder on the stability of alloyed perovskite solar cells
Source: Nat Energy. 2024 Oct 30;10(1):66–76. doi: 10.1038/s41560-024-01660-1 (PMC11774756; doi:10.1038/s41560-024-01660-1)
Supplement: Supplementary file 2 — Reporting Summary [file 41560_2024_1660_MOESM2_ESM.pdf]

nature portfolio

Corresponding author(s): Miguel Anaya, Samuel D. Stranks

Solar Cells Reporting Summary

Nature Portfolio wishes to improve the reproducibility of the work that we publish. This form is intended for publication with all accepted papers reporting the characterization of photovoltaic devices and provides structure for consistency and transparency in reporting. Some list items might not apply to an individual manuscript, but all fields must be completed for clarity.

For further information on Nature Research policies, including our data availability policy, see Authors & Referees.

Experimental design

Please check the following details are reported in the manuscript, and provide a brief description or explanation where applicable.

1. Dimensions

Area of the tested solar cells

☒ Yes — Methods section. 0.16 cm^2
☐ No — *Explain why this information is not reported/not relevant.*

Method used to determine the device area

☒ Yes — Methods section. Active area defined by overlap of rectangular patterned ITO substrate with rectangular. Area confirmed with calibrated optical microscope.
☐ No — *Explain why this information is not reported/not relevant.*

2. Current-voltage characterization

Current density-voltage (J-V) plots in both forward and backward direction

☒ Yes — All current voltage characteristics and optically extracted current voltage characteristics are reported with reverse and forward direction scans
☐ No

Voltage scan conditions

☒ Yes — Devices were scanned reverse then forward. Under solar sim, devices were scanned with 20 mV steps, 20 ms integration, 20 ms delay time between voltage step and measurement. During voltage dependent JV measurements, the scan rate was 0.01 V/s with a delay to take each image between voltage steps
☐ No — *Explain why this information is not reported/not relevant.*

Test environment

☒ Yes — Methods section. Devices were JV tested in ambient air, room temperature, ~50% relative humidity
☐ No — *Explain why this information is not reported/not relevant.*

Protocol for preconditioning of the device before its characterization

☐ Yes — *Provide a description of the protocol.*
☒ No — No intentional preconditioning

Stability of the J-V characteristic

☒ Yes — Each device was JV scanned very slowly during microscopy multiple times and for pristine devices before stress testing, consecutive JV curves over 1-2 hours illumination on the same scan are generally reproducible.
☐ No — *Explain why this information is not reported/not relevant.*

3. Hysteresis or any other unusual behaviour

Description of the unusual behaviour observed during the characterization

☒ Yes — Large hysteresis observed in some devices after stress testing. Slow transient rise in open circuit voltage observed in some device stacks after stress testing
☐ No — *Explain why this information is not reported/not relevant.*

Related experimental data

☒ Yes — Reverse and forward JV scans shown in JV data in main text and SI. Consecutive JV scans after stress testing shown in Supplementary Figure 80
☐ No — *Explain why this information is not reported/not relevant.*

4. Efficiency

External quantum efficiency (EQE) or incident photons to current efficiency (IPCE)

☐ Yes — *Provide a description of the technique used.*
☒ No — EQEs were not reported. Short circuit current values matched within experimental error when the same devices were measured under calibrated solar simulators in Helmholtz Zentrum Berlin and Cambridge

A comparison between the integrated response under the standard reference spectrum and the response measure under the simulator

☐ Yes
☒ No

State where this information can be found in the text.

Explain why this information is not reported/not relevant.

For tandem solar cells, the bias illumination and bias voltage used for each subcell

☐ Yes
☒ No

Provide a description of the measurement conditions.

Explain why this information is not reported/not relevant.

5. Calibration

Light source and reference cell or sensor used for the characterization

☒ Yes
☐ No

Methods section. The intensity was calibrated to AM1.5G 1-sun-equivalent with a KG3 filtered Silicon reference solar cell

Explain why this information is not reported/not relevant.

Confirmation that the reference cell was calibrated and certified

☒ Yes
☐ No

Methods section. Reference cell was calibrated by Fraunhofer ISE

Explain why this information is not reported/not relevant.

Calculation of spectral mismatch between the reference cell and the devices under test

☒ Yes
☐ No

Methods section. Spectral mismatch is ~0.997, within experimental error so no correction was applied.

Explain why this information is not reported/not relevant.

6. Mask/aperture

Size of the mask/aperture used during testing

☐ Yes
☒ No

Report the size of the mask/aperture.

Methods section. Shadow mask was not used. As per our previous reports, unmasked short circuit current matches EQE measured of these devices

Variation of the measured short-circuit current density with the mask/aperture area

☐ Yes
☒ No

Report the difference in the short-circuit current density values measured with the mask and aperture area.

Explain why this information is not reported/not relevant.

7. Performance certification

Identity of the independent certification laboratory that confirmed the photovoltaic performance

☐ Yes
☒ No

Identify the independent certification laboratory.

No certified devices

A copy of any certificate(s)

☐ Yes
☒ No

Certificate copies should be provided in the Supplementary information. Please state the supplementary item number.

Explain why this information is not reported/not relevant.

8. Statistics

Number of solar cells tested

☒ Yes
☐ No

Supplementary Figure 38, Main Text Figure 4

Explain why this information is not reported/not relevant.

Statistical analysis of the device performance

☒ Yes
☐ No

Supplementary Figure 38, Main Text Figure 4

Explain why this information is not reported/not relevant.

9. Long-term stability analysis

Type of analysis, bias conditions and environmental conditions

☒ Yes
☐ No

Figure 4, Methods section. ISOS-L-2I protocol, 1 sun illumination, 65 C, open circuit voltage with nitrogen flow

Explain why this information is not reported/not relevant.
